# PGC-1 alpha overexpression in the skeletal muscle results in a metabolically active microbiome which is independent of redox signaling

**DOI:** 10.1038/s41598-025-05594-w

**Published:** 2025-07-01

**Authors:** Erika Koltai, Soroosh Mozaffaritabar, Lei Zhou, Attila Kolonics, Atsuko Koike, Kumpei Tanisawa, Jonguk Park, Ferenc Torma, Zsolt Radak

**Affiliations:** 1 Research Institute of Sport Science, Hungarian University of Sport Science, Alkotas Street, 42-46, Budapest, 1123 Hungary; 2 Faculty of Sport Sciences, Waseda University, Tokorozawa, Japan; 3 Artificial Intelligence Center for Health and Biomedical Research, Osaka, Japan; 4 Institute of Sport Sciences and Physical Education, Faculty of Sciences, University of Pécs, Pécs, 247624 Hungary; 5 Department of Bioengineering, Sapientia Hungarian University of Transylvania, Piata 26 Libertatii, 530104 Miercurea Ciuc, Romania

**Keywords:** Mitochondria, Microbiota, PGC-1α, Physical exercise, Host–microbial interaction

## Abstract

In this study, we investigated the potential relationship between the mitochondrial network and the microbiome using wild-type and skeletal muscle-specific PGC-1α (Pparg coactivator 1 alpha) overexpressing mice, both with and without exercise training. Basal PGC-1α levels were significantly higher in the skeletal muscle (J Physiol Biochem 80:329–335, 2024. 10.1007/s13105-024-01006-1) and, notably, in the colon, which is anatomically proximal to the microbiome. However, no significant changes were observed in cell signaling or mitochondria-related proteins within the colon. On the other hand, mitochondrial H₂O₂ production in the colon decreased in the PGC-1α overexpressing group. The relative abundance of several bacterial taxa differed between wild-type and PGC-1α overexpressing groups at baseline condition, indicating a shift in the microbiome milieu probably to cope with the increased metabolism, enhanced short-chain fatty acid utilization, and improved endurance capacity. Ten weeks of exercise training differentially modulated the host microbiome in PGC-1α overexpressing and wild-type mice, facilitating adaptations to a broad range of exercise-induced challenges. The results of this study provide new insights into the possible cross-talk between mitochondria and the microbiome.

**Supplementary Information:**

The online version contains supplementary material available at 10.1038/s41598-025-05594-w.

## Introduction

The microbiota of the gut is crucial for breaking down dietary nutrients, regulating intestinal and systemic immune responses, producing small molecules critical for intestinal metabolism, and generating several gases that can modulate cellular function^1^. Due to the complex function of the gut microbiome, microbial diversity can be defined as the variety of different unicellular organisms, including bacteria, archaea, protists, and fungi^2^.

The evolutionary interactions between eukaryotes and bacteria have fostered mutual benefits, resulting in a dynamic yet safe system known as symbiosis^3^. It is suggested that mitochondria were once free-living bacteria, based on shared structural and functional features. Over time, they transitioned into an endosymbiotic state and became an integral organelle within early eukaryotic cells^3^. This highly probable endosymbiosis enabled unique characteristics of mitochondria, including their role in extracellular communication. It is suggested that one of the targets of this communication is the ancient family of eukaryote-hosted bacterial species^4^. Indeed, it has been shown that mutations in mtDNA and the mitochondrial genotype are associated with the diversity of bacterial species in the gut microbiome of mice^4^. One possible mode of communication could be via reactive oxygen species (ROS), as mitochondria-produced ROS play an important role in the innate immune response, which is often targeted by pathogenic bacteria, leading to altered regulation of the gut epithelial barrier^5^.

To better understand the potential interactions between the mitochondrial network and the gut microbiome, we employed transgenic mice with muscle-specific overexpression of PGC-1α In this model, we described the effects of PGC-1α overexpression on lipid metabolism of the skeletal muscle^6^. In the present paper, we explain the results of shotgun metagenomic analysis of the microbiome, the possible cross-talk of mitochondria and microbiome, and the associated cellular pathways in the intestine. Given that PGC-1α overexpression is thought to affect the motor activity of mice, we included trained groups to distinguish the effects of increased mitochondrial mass due to PGC-1α overexpression from those resulting from exercise-induced adaptive responses.

## Material and methods

### Animal model

Twenty 10-month-old C57BL/6-Tg(Ckm-Ppargc1a)31Brsp/J mice with skeletal muscle-specific PGC-1α overexpression and twenty age-matched wild-type littermates were randomly assigned to four experimental groups: Wild-type Control (Wt-C), PGC-1α Control (PGC-1α-C), with 11 animals per control group (n = 11), and two exercise groups: Wild-type Exercise (Wt-Ex) and PGC-1α Exercise (PGC-1α-Ex), each consisting of 9 animals (n = 9). Animals were purchased from The Jackson Laboratory (Bar Harbor, Maine, U.S https://www.jax.org/strain/008231 ) in this mice model PGC-1α overexpression is driven by the mouse muscle creatine kinase (MCK) promoter^7^. The mice were housed under a 12-h light/dark cycle with ad libitum access to standard laboratory chow and water. The study protocol was approved by the National Animal Research Ethical Committee of Hungary (PE/EA/62–2/2021), and all methods were performed in accordance with the relevant national and international guidelines and regulations. Additionally, all experiments and procedures were conducted in compliance with the ARRIVE guidelines.

### Training protocol

After familiarization with the treadmill, mice in the training groups underwent a fatigue endurance test to assess their maximal running capacity, following the protocol described by Dougherty et al.^8^. Briefly, the protocol consists of three days of running habituation, followed by one day of rest and a final test day. Based on the results, the training protocol was initiated at 60% of the animals’ average maximal running capacity, with the exercise intensity progressively increased on a weekly basis. The training regimen lasted for 10 weeks, consisting of 5 days of 30-min sessions per week as reported by Mozaffaritabar et al.^6^. Fecal samples were collected before the start and after the completion of the exercise intervention. After overnight fasting, animals were deeply anesthetized with intraperitoneal Ketamine (100 mg/kg) and Xylazine (10 mg/kg) injection, followed by euthanasia via cervical dislocation; subsequently, their intestines were harvested, flash‐frozen in liquid nitrogen, and stored at − 80 °C for further analysis.

### Western blots

Western blots were performed as previously described^9^ using the following antibodies: PGC-1α (nbp1-04676), CS (ab96600), Mfn1 (sc50330), GAPDH (9001-50-7), β-Actin (sc69879), p-MTOR/MTOR (cst5536, 2983), p-AKT/AKT (cst9271, 4691), p-CREB/CREB (cst9198, 9197s), p-AMPKα/AMPKα (2535, 2532), CBS (14782), TFAM (PA5-27865), and PCNA Antibody FL-261 (sc-7907).

### Mitochondrial, cytosolic, and nuclear fraction preparation

Cell fractionation was performed according to Scoranno et al.^10^ with minor modifications. Every step was performed at 4 °C. Briefly, the fresh, fat, and connective tissue-free colon tissue was immersed in ice-cold PBS supplemented with 10 mM EDTA and minced into small pieces. Samples were digested by 0.05% trypsin for 30 min with gentle shaking, then centrifuged at 1000 g for 5 min. The pellet was resuspended in a tenfold buffer volume of IB_m_1 (50 mM Tris–HCl, 50 mM KCl, 10 mM EDTA, 0.2% BSA and 0.067 M Sucrose pH 7.4) and homogenized by 3–4 times gentle stroke. The homogenate was centrifuged at 600 g for 10 min. Part of the nucleus including pellet and cytosolic supernatant was reserved for Western blot analysis. The supernatant was centrifuged at 8000 g for 10 min which resulted in the mitochondrial pellet. The centrifugation step was repeated after IB_m_1 buffer homogenization to gain high-quality intact mitochondria. The mitochondrial pellet was suspended in the least volume of possible IB_m_2 buffer (10 mM Tris–HCl, 3 mM Tris-EGTA, and 0.25 M Sucrose pH 7.4). Protein concentration was measured using the Bradford assay^11^.

### ROS production measurement

Mitochondria (0.3 mg/ml) were incubated in experimental buffer (10 mM Tris/HCl, 5 mM MgCl_2_, 2 mM KH_2_PO_4_, 20 mM EGTA/Tris, 250 mM Sucrose pH 7.4) supplemented with 1 μM Amplex Red (excitation: 560 nm; emission: 584 nm) and horseradish peroxidase (10 IU) to assess ROS production by monitoring H_2_O_2_-induced fluorescence according to Votyakova et al. with minor modifications^12^. After measuring basal ROS production, 10 mM succinate (Succ) and/or 1 μM (Rote) were sequentially added. With succinate as a substrate, ROS production is augmented due to reverse electron transport (RET) at complex I. This can be estimated by its sensitivity to inhibition by rotenone. Under these conditions, the addition of rotenone has two effects at complex I: it enhances ROS production linked to the forward electron flux while reducing ROS production associated with reverse electron flux^13^. Calibration of H_2_O_2_ production was obtained by the addition of a known amount of H_2_O_2_. Fluorimetric assays were performed at 30 °C with Fluorskan Ascent FL fluorimeter on 96 well plates. Each sample was measured in triplicate.

### Microbiome assay

Fecal samples were collected for analysis of gut microbiota in cryo tubes and stored at − 80 °C until subsequent analysis. A frozen aliquot (200 mg) of each fecal sample was suspended in 250 ml of guanidine thiocyanate solution, 0.1 M Tris, pH 7.5, and 40 ml of 10% N-lauroyl sarcosine. DNA extraction was then performed as previously described^14^ and the DNA concentration and molecular size were estimated using a nanodrop (Thermo Scientific) and agarose gel electrophoresis.

### Illumina sequencing

Extracted fecal DNA was used as input for the Illumina Nextera® XT DNA Library Preparation Kit to construct indexed paired-end libraries, following previously established protocols^15^. DNA library preparation followed the manufacturer’s instructions (Illumina). The workflow indicated by the provider was used for cluster generation, template hybridization, isothermal amplification, linearization, blocking and denaturing, and hybridization of the sequencing primers. The base-calling pipeline (version IlluminaPipeline-0.3) was used to process the raw fluorescent images and call sequences.

### Bioinformatics analysis

The quality of raw and trimmed reads was assessed using FastQC and MultiQC. Low-quality sequences were filtered and trimmed with Trimmomatic, removing sequences with a minimum length < 36 bp and low-quality base calls (Phred score < 30). Reads aligning to the human reference genome (GRCh38) were removed to eliminate host contamination using Bowtie2 (v2.4.2). Shotgun metagenomic sequencing data were analyzed for microbiome composition using Kraken2- Bracken, as previously described^16^ and functional genomic analysis as described by FMAP^17^. Taxa with an average relative abundance of less than 1% across all samples were excluded from further analysis.

### Statistical analysis

Data distribution was tested using the Shapiro–Wilk test to assess normality. After confirming normal distribution factorial ANOVA was conducted to assess timepoint and group differences with Tukey HDS to compare means. For microbiome data and other non-normally distributed variables, the Kruskal–Wallis test was used. Benjamini–Hochberg correction was used to adjust for multiple comparisons, with false discovery rate set at FDR < 0.05. Taxa with log₂ fold changes greater than 0.5 or less than − 0.5 were considered biologically relevant.

## Results

**Fig. 1:**
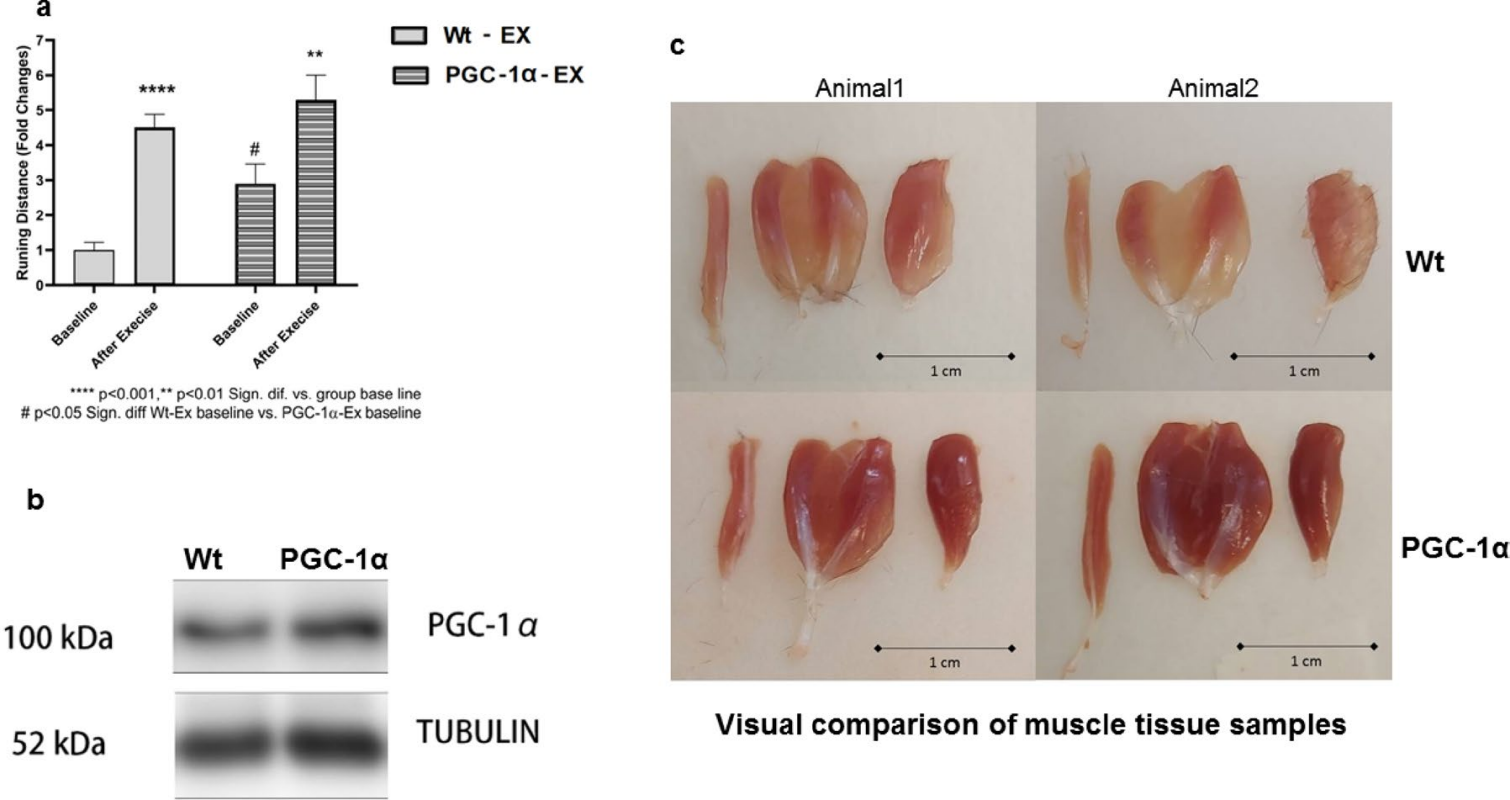
Phenotype and running distance of PGC-1 alpha and wild-type of mice. PGC-1 alpha overexpressed animals had significantly better endurance performance than that of wild-type mice. Ten weeks of exercise training increased the endurance performance in both groups (**a**). Representative plantaris, gastrocnemius and tibialis anterior muscles (left to right) of the PGC-1α transgenic and wt control mice (**c**). Representative PGC-1α the protein levels were showed higher tendencies in the quadriceps of transgenic mice (**b**). Bars depict mean ± SEM.

The baseline running distance of the PGC-1α-Ex group was 2.9-fold longer than that of the wt-Ex group, showing a significant improvement. After 10 weeks of exercise training, the running distance of the PGC-1α-Ex group increased significantly compared to both its baseline and the baseline of the wt-Ex group, with no statistical difference compared to the post-exercise values of the wt group. Figure 1 presents the results of the exhaustive running test (1a), representative PGC-1α levels from quadriceps muscle for control mice in both PGC-1α overexpressing and wt mice (1b), and skeletal muscle visuals of the hindlimb (1c) including the plantaris, gastrocnemius and tibialis anterior muscle representing the wt-C and PGC-1α-C groups. Our results show muscle-specific outcomes as published in detail by Mozaffaritabar et al.^6^ from the same investigation.

**Fig. 2:**
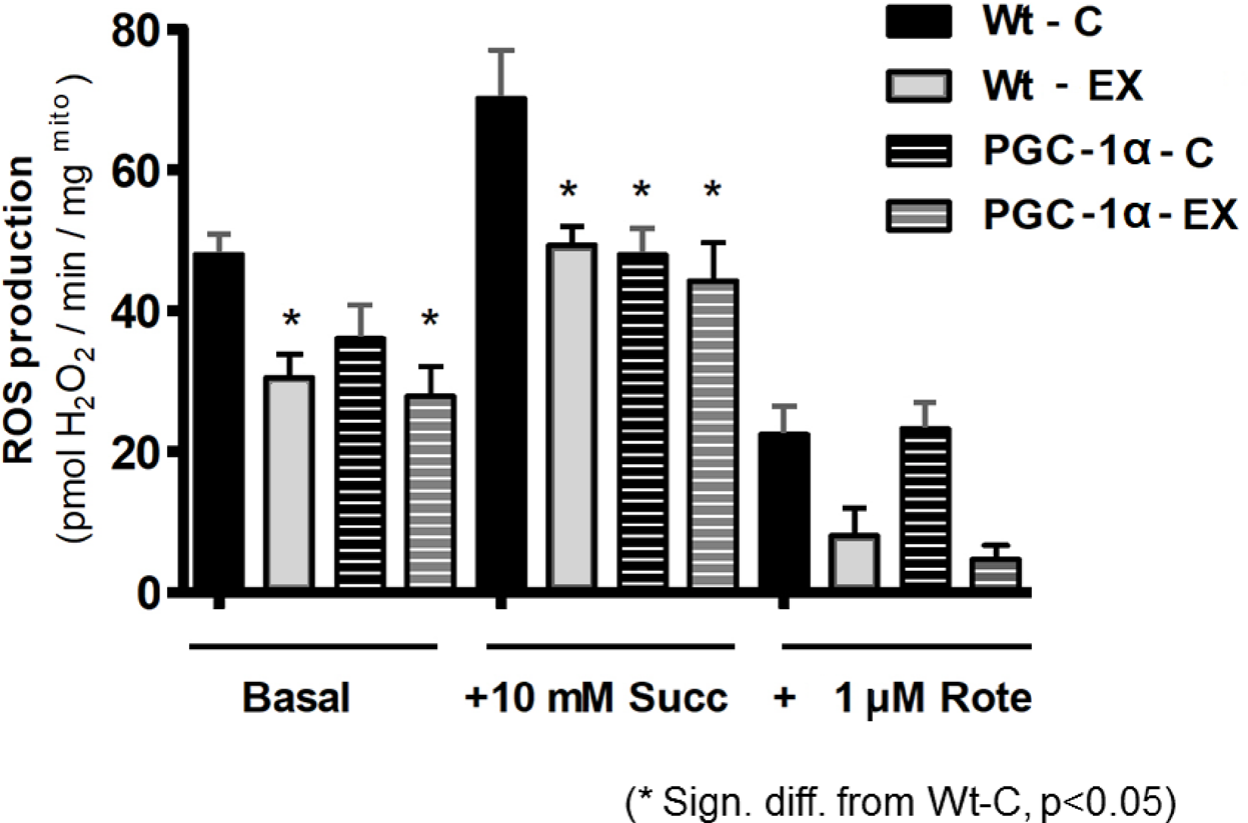
Effects of muscle-specific PGC-1α overexpression and exercise on mitochondrial ROS production in the intestine. Intact mitochondria were isolated from the intestine, (see Materials and Methods section) and incubated (0.1 mg protein) in 0.2 mL medium (30 °C) containing 250 mM sucrose, 0.1 mM EGTA, 20 mM Tris–HCl (pH 7.4), 2.5 mM Pi and 1 mM MgCl_2_. Basal and 10 mM succinate (Succ) induced as well as 1 μM rotenone (Rote) inhibited hydrogen peroxide (ROS) production was assessed by monitoring H_2_O_2_-induced fluorescence of 1 μM Amplex Red in the presence of horseradish peroxidase. Results are mean ± SEM (n = 4–5). Statistical significance was assessed using Oneway ANOVA .

We examined the microbiome in close proximity to the colon. The biochemical analysis revealed that exercise and PGC-1α overexpression significantly decreased basal and succinate-induced ROS production in colon mitochondria (Fig. 2).

**Fig. 3:**
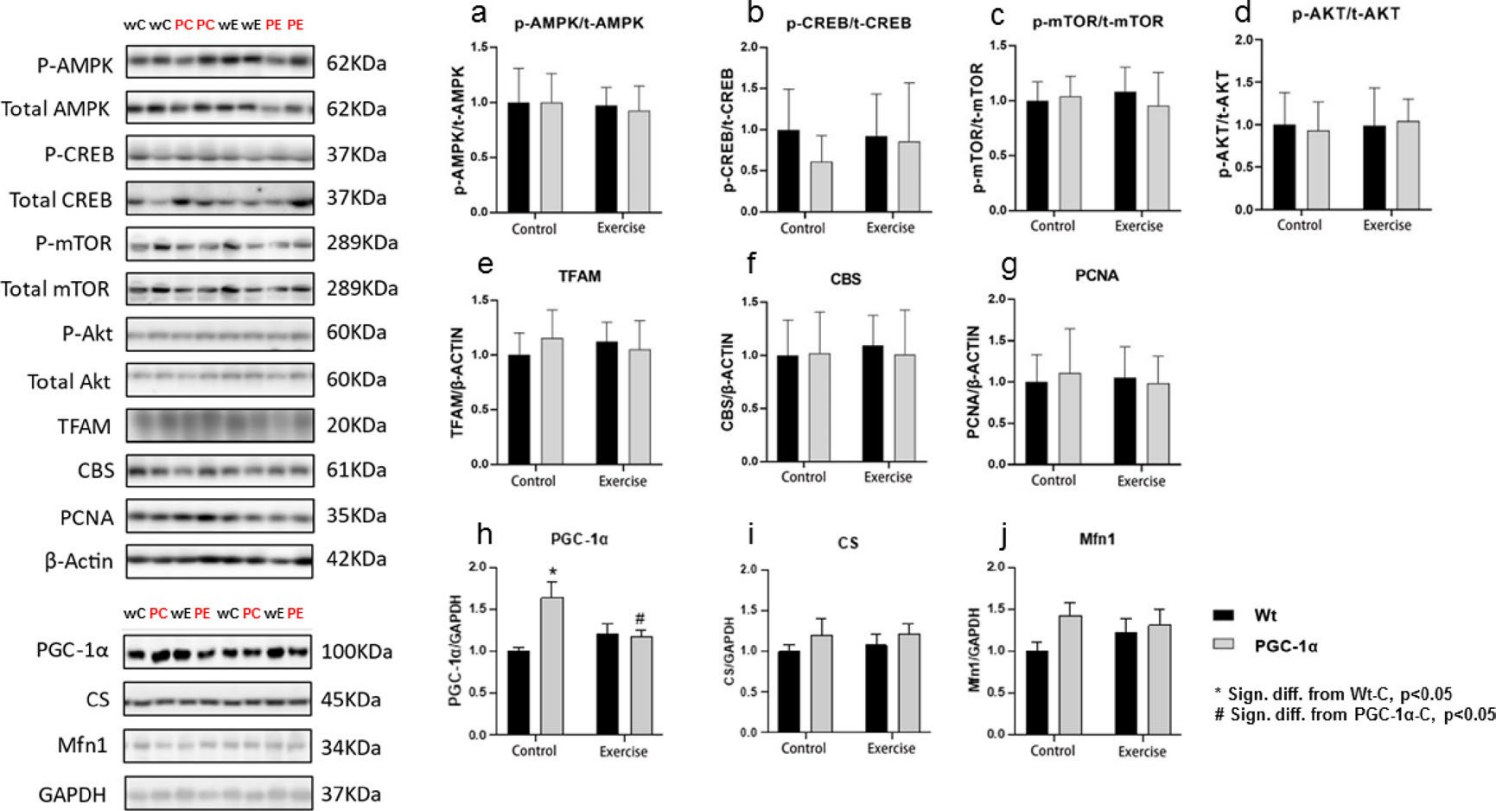
Effects of exercise and muscle-specific PGC-1α overexpression on protein levels and phosphorylation in the large intestine. Except for PGC-1α (h), protein and phosphorylation levels in colon samples did not show significant differences between the exercise (Control, Exercise) and genotype (Wt, PGC-1α) groups (**a**–**g**, **i**–**j**) after the exercise intervention period. wC: Wilt type control, wE: wild type exercise, PC: PGC-1α control, PE: PGC-1α exercise. Bars depict mean ± SEM.

Except for PGC-1α, no significant alterations were detected in the main exercise-associated adaptive proteins in the colon related to PGC-1α overexpression or exercise training, as measured after the exercise intervention (Fig. 3).

**Fig. 4:**
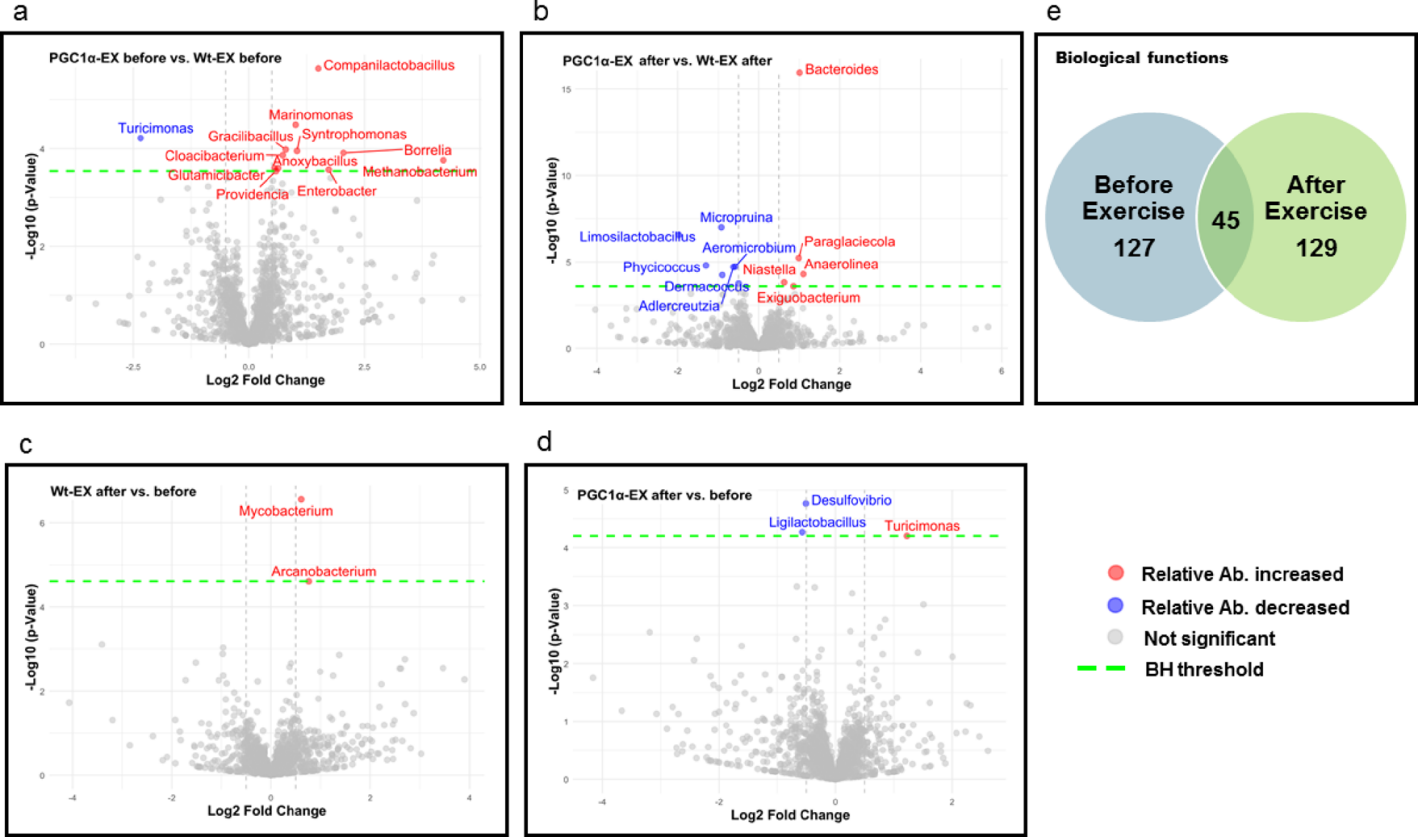
Effects of muscle specific PGC-1α over expression and exercise on gut microbiome composition. **a**–**d** panels show the changes in relative abundance density at the genus taxonomic level. Red dots indicate significantly increased relative abundance, while blue dots significantly decreased relative abundance between the groups depicted in the upper right corner of each panel. The green dashed line marks the Benjamini-Hochberg (BH) correction threshold, and gray lines indicate ± 0.5 Log2 fold change. Panel **e** depicts the number of significantly different biological pathways between the PGC-1α and Wt mice before and after the exercise intervention.

The intestine is in proximity to the gut microbiome. Therefore, we investigated the cell signaling pathways in the intestine and performed shotgun metagenomic analysis to investigate the effects of PGC-1α muscular overexpression and exercise training on microbiome plasticity. The observed differences in the relative abundance of several bacterial genera may reflect an influence of PGC-1α overexpression, the increased number of mitochondria in the skeletal muscle of the mice, as housing and animal chow were the same for the wild-type and PGC-1α overexpressed groups (Fig. 4, Supplementary Table S1). Based on this finding, one can speculate about a possible interaction between the mitochondrial network in the skeletal muscle and the microbiome of gut. The relative abundance of *Campanilactobacillus, Marinomonas, Gracilibacilus, Cloacibacterium, Glutamicibacter, Providencia, Anoxybacillus, Syntrohomonas, Borrelia, Enterobacter, Methonobacterium*, and *Turicimonas* differed at baseline between wild-type and PGC-1α overexpressing mice (Fig. 4 panel a). Moreover, we detected exercise-induced alterations in the microbiome, revealing distinct adaptability between wild-type and PGC-1α-overexpressing animals. Indeed, following exercise training, the relative abundance of *Micropruina, Limosilactobacillus, Aeromicrobium, Phycicoccus, Dermacoccus*, and *Adlercreutzia* was lower, while *Bacteroides*, *Paraglaciecola*, *Niastella*, *Anaerolinea*, and *Exiguobacterium* were increased in the PGC-1α overexpressing group compared to wild-type mice (Fig. 4, Panel B).This suggests that exercise training may enhance the dynamics of the microbiome in PGC-1α overexpressing animals compared to wild-type controls.

Exercise training increased the relative abundance of *Mycobacterium* and *Arcanobacterium* in the wild-type group (Fig. 4, Panel c), and *Turicimonas* in the PGC-1α overexpressing group, while the abundance of *Desulfovibrio* and *Ligilactobacillus* decreased in the PGC-1α overexpressing group (Fig. 4, Panel c, d).

Genotype and regular exercise also appear to influence molecular pathways, with a greater number of significantly altered microbiome-related pathways observed between the muscle-specific PGC-1α overexpression group and the control group, both before and after the intervention, compared to the differences observed within each group before and after exercise. (Supplementary FigS1, Table S2). In total, 127 pathways differed significantly at baseline, with 28 pathway-associated bacterial groups showing increased representation and 99 showing decreased representation in the PGC-1α overexpressing mice compared to wild-type controls. Following the exercise intervention, 129 pathways were significantly different, with 90 showing increased and 39 showing decreased representation. Notably, 45 pathways exhibited persistent differences across the exercise treatment period, the majority of which (41 pathways) showed elevated representation in the overexpression group, while only 4 showed lower representation. (Fig. 4 Panel e).

## Discussion

It is suggested, based on the evolutionary origins of the cell organelle, that communication exists between gut-hosted bacterial flora and the mitochondria. However, a definitive link has yet to be fully established. To the best of our knowledge, this study provides preliminary evidence that may support the existence of communication between mitochondria and the microbiome.

PGC-1α overexpression has been linked to significant changes in the gut microbiome, particularly in the relative abundance of specific microbial genera. This suggests that the host’s metabolic environment may undergo substantial shifts, as PGC-1α is crucial for regulating energy metabolism, mitochondrial biogenesis, and oxidative metabolism^18,19^. The physiological effects of PGC-1α overexpression in skeletal muscle appear to be reflected in the gut microbiota, suggesting a metabolic environment characterized by enhanced energy extraction, fermentation, and short-chain fatty acid (SCFA) production. These changes likely contribute to enhanced metabolic efficiency and improved gut health, which aligns with the significantly higher baseline endurance observed in PGC-1α overexpressing mice. Recent studies have highlighted that PGC-1α overexpression in skeletal muscle is linked to increased levels of GPR41, a receptor specialized for SCFA uptake, suggesting a direct relationship between enhanced muscle mitochondrial biogenesis and SCFA utilization^6^. Our observation of differences in the gut microbiome composition between PGC-1α overexpressed and wild-type mice aligns with the findings of an earlier study, which showed that AC5KO mice—known for their improved longevity, increased glucose metabolism, insulin sensitivity, and exercise tolerance—also exhibit distinct changes in their gut microbiome profile^20^. This supports the notion that metabolic adaptations—such as those mediated by PGC-1α overexpression—may influence the composition of the gut microbiome.

The differential response of the microbiome to exercise between wild-type and PGC-1α-overexpressing animals suggests that PGC-1α influences the way the microbiome responds to physical activity, potentially impacting host physiology. The decreased abundance of *Micropruina, Limosilactobacillus, Aeromicrobium, Phycicoccus, Dermacoccus,* and *Adlercreutzia* might indicate a reduction in certain metabolic activities or immune responses. For example, *Limosilactobacillus* is known for its probiotic properties and its role in maintaining gut health^21,22^. *Micropruina* has been suggested to were use to carbon from sugars and amino acids, under anaerobic conditions, to fermentation to lactic acid, acetate, propionate, and ethanol, and partly stored as glycogen for potential aerobic use^23^. Recent data revealed that *Aeromicrobium*, is important part of immune system since it acts effectively against H9N2 influenza virus in mice^24^. It has been reported that one of the metabolites of *Dermacoccus* bacterium, the demacozines play a role in the regulation of redox homeostasis^25^. A decrease in these bacteria could suggest a shift away from these functions, potentially impacting gut homeostasis and immune regulation.

The increased abundance of *Bacteroides, Paraglaciecola, Niastella, Anaerolinea,* and *Exiguabacterium* could be associated with enhanced metabolic activities related to energy production and nutrient absorption. *Bacteroides* are crucial for breaking down complex molecules in the gut, which can influence energy balance and metabolic health^26^. In deed, *Bacteroidetes* efficiently breaks down poly- and mono-saccharides into beneficial SCFAs^27^ like acetate and propionate that could play a role in the prevention of colon cancer^28^ and it could enhace endurance capacity^29,30^.

The fact that exercise training differently modulates the microbiome of wild-type and PGC-1α-overexpressing mice indicates that exercise training provides a different physiological signal to the microbiome than the increased levels of mitochondrial formation. Exercise training provides intermittent metabolic challenges, while higher mitochondrial content in the skeletal muscle could imply continuous cross talks between mitochondria and microbime.

Interestingly, it has been noted that different lipid metabolism-related pathways were influenced by PGC-1α overexpression and exercise training^6^. Exercise training increased the relative abundance of *Mycobacterium* and *Arcanobacterium* in wild, and *Turicimonas,* in PGC-1α overexpressed group while the *Desulfovibro* and *Ligalactobacillus* content decreased by training in this group. Mycobacterium and Arcanobacterium, which showed increased abundance with exercise training in wild-type mice, are not typically prominent in the gut microbiome but can be transiently present. Some species from these taxa may contribute to immune modulation, potentially supporting exercise-induced anti-inflammatory effects. Research suggests that *Mycobacterium* can influence T-cell responses and enhance immune resilience, potentially benefiting metabolic and immune adaptations to exercise^31^. *Arcanobacterium* is less well-studied in the intestinal tract, but its presence may indicate shifts in niche microbial dynamics due to exercise, possibly favoring microbes with versatile metabolic functions that respond positively to exercise-induced alterations in host physiology. The role of *Arcanobacterium* in gut health remains unclear, but its relative expansion may be linked to exercise-induced changes in nutrient availability and gut pH. *Turicimonas,* which showed increased abundance following exercise training, is associated with butyrate production—a beneficial factor for colonic health and energy metabolism*.*^32^. Butyrate has anti-inflammatory properties and contributes to maintaining gut barrier integrity. The increase in *Turicimonas* abundance could support the anti-inflammatory and metabolic benefits associated with PGC-1α overexpression, enhancing the host’s endurance capacity and energy regulation^32^. This may also align with findings that PGC-1α overexpression leads to greater mitochondrial biogenesis, complementing the energetic requirements of exercise adaptation. Concurrently, the decreased abundance of *Desulfovibrio*, which is known for producing hydrogen sulfide^33^ and *Ligilactobacillus*, involved in immune regulation^34^, in the transgenic group suggests a shift to a bacterial environment that maintains a more stable and less pro-inflammatory microbiome, especially under the high metabolic demands of PGC-1α overexpression. *Ligilactobacillus* species, previously classified as Lactobacillus, are generally regarded as beneficial probiotics. The relative decline in *Ligilactobacillus* due to exercise may suggest that PGC-1α overexpression alters gut ecology, potentially reducing the niche for these bacteria. This shift could be attributed to an altered metabolic environment, and there is a possibility that PGC-1α, due to its enhancing effect on fatty acid oxidation, may reduce the need for lactate-producing bacteria.

Our data suggest that PGC-1α overexpression decreases mitochondria-derived ROS production in the colon, possibly ruling out ROS-associated signaling pathways that could account for the different compositions of the microbiome between wild-type and PGC-1α overexpressed animals.

Overall, our data indicate that PGC-1α overexpression in skeletal muscle alone, without exercise treatment, could contribute to a physiological environment—such as improved oxygen utilization and reduced ROS production—that may lead to changes in the microbiome, potentially supporting metabolic activity, SCFA utilization, and improved endurance capacity. Exercise training seems to differentially modulate the host microbiome in PGC-1α overexpressing and wild-type mice, which may reflect coping mechanisms to exercise-induced physiological challenges. The results of the present investigation, together with recent advances in the field, suggest a potential cross-talk between mitochondria and the microbiome; however, further studies are required to further elucidate the underlying biological mechanisms.

## Electronic supplementary material

Below is the link to the electronic supplementary material.

Supplementary Material 1

Supplementary Material 2

Supplementary Material 3

## Data Availability

The datasets generated during the current study are available in the The European Nucleotide Archive (ENA) repository, with accession number ERP169114: https://www.ebi.ac.uk/ena/browser/view/PRJEB85739.

## Abbreviations

PGC-1α: Peroxisome proliferator-activated receptor gamma coactivator 1-alpha
ROS: Reactive oxygen species
mtDNA: Mitochondrial DNA
Wt: Wild type
SCFA: Short-chain fatty acid
GPR41: Free fatty acid receptor 3
FMAP: Functional mapping and analysis pipeline for metagenomics and metatranscriptomics studies
Succ: Succinate
Rote: Rotenone
CS: Citrate synthase
Mfn1: Mitofusin-1
GAPDH: Glyceraldehyde 3-phosphate dehydrogenase
MTOR: Mammalian target of rapamycin
AKT: Protein kinase B
CREB: CAMP response element-binding protein
AMPKα: 5′ AMP-activated protein kinase alpha
CBS: Cystathionine-β-synthase
TFAM: Mitochondrial transcription factor A
PCNA: Proliferating cell nuclear antigen
