# Supplementary material for: PGC-1 alpha overexpression in the skeletal muscle results in a metabolically active microbiome which is independent of redox signaling
Source: Sci Rep. 2025 Jul 1;15:20527. doi: 10.1038/s41598-025-05594-w (PMC12215982; doi:10.1038/s41598-025-05594-w)
Supplement: Supplementary file 1 — Supplementary Material 1 [file 41598_2025_5594_MOESM1_ESM.pdf]

Supplementary information

Table S1 Taxon comparison of treatment groups at the genus level
Columns depict taxon name, log 2 fold changes, the unmodified p value of group comparison, and Benjamini-Hochberg corrected p values

Table S2 Biological pathway comparison of treatment groups at the genus level
Columns depict biological pathway name, log 2 fold changes, the unmodified p value of group comparison, and Benjamini-Hochberg corrected p values

Figure S1 Effects of muscle specific PGC-1α over expression and exercise on gut microbiological biological pathways. a-d panels show the changes in the abundance of bacterial pathways in the gut microbiome at the genus level. Red dots represent significantly increased abundance, while blue dots indicate significantly decreased abundance between the groups depicted in the upper right corner of each panel. The green dashed line marks the Benjamini-Hochberg (BH) correction threshold, and gray lines indicate ±0.5 Log2 fold change.

Original western blot images. Red underlines indicate the bands selected and shown in Figure 3 a-j and Figure 1 b.

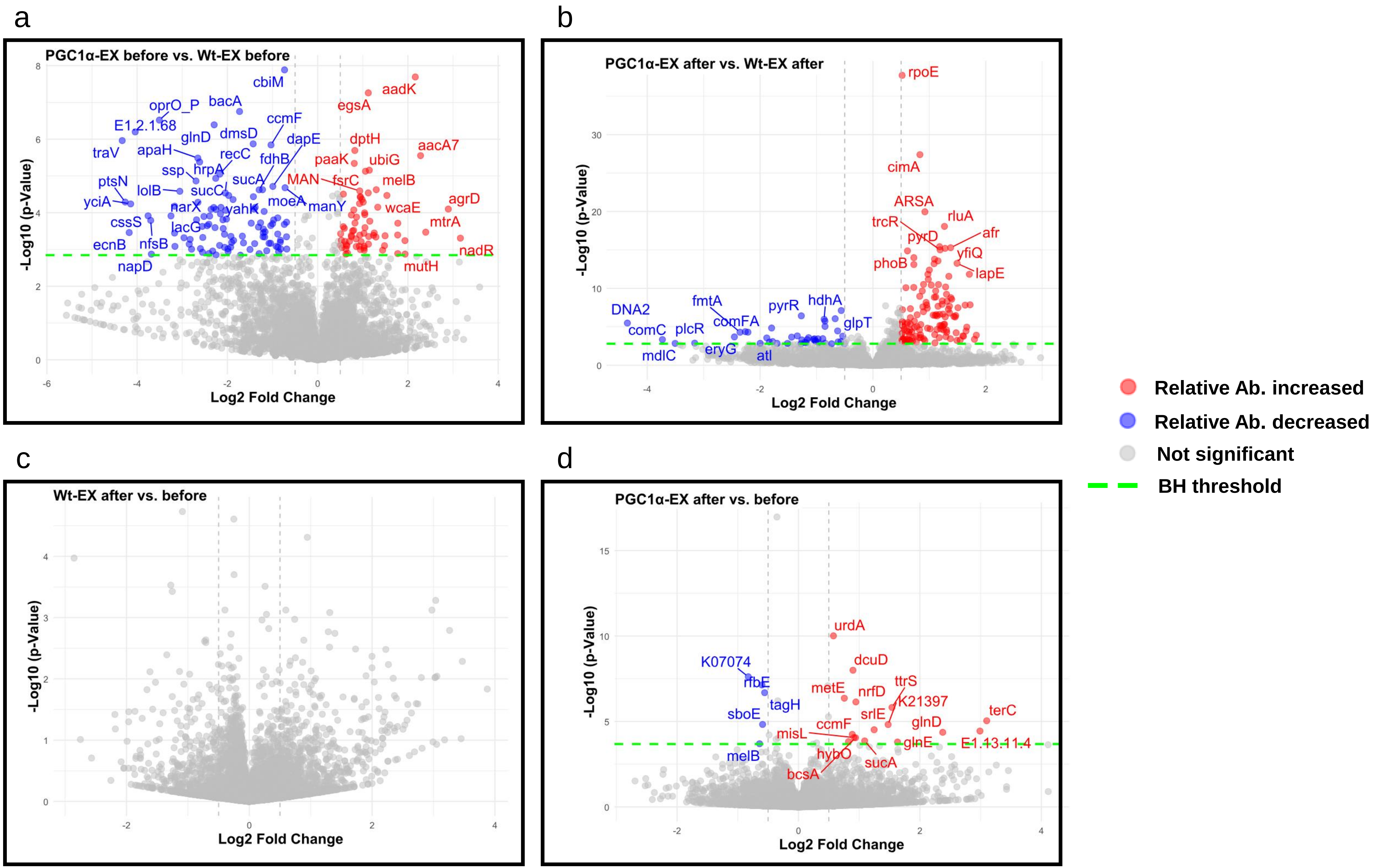

a
PGC1α-EX before vs. Wt-EX before
cbiM
aadK
egsA
oprO_P
bacA
E1.2.1.68
ccmF
dmsD
glnD
dapE
dptH
aacA7
traV
apaH
recC
fdhB
paaK
ubiG
ssp
hrpA
sucA
MAN
fsrC
melB
ptsN
lolB
sucC
agrD
yciA
narX
yahK
moeA
manY
wcaE
cssS
lacG
mtrA
ecnB
nfsB
nadR
napD
mutH
-Log10 (p-Value)
Log2 Fold Change
b
PGC1α-EX after vs. Wt-EX after
rpoE
cimA
ARSA
trcR
rluA
pyrD
afr
yfiQ
phoB
lapE
DNA2
fmtA
pyrR
hdhA
comC
plcR
comFA
glpT
mdlC
eryG
atl
-Log10 (p-Value)
Log2 Fold Change
Relative Ab. increased
Relative Ab. decreased
Not significant
BH threshold
c
Wt-EX after vs. before
-Log10 (p-Value)
Log2 Fold Change
d
PGC1α-EX after vs. before
urdA
K07074
dcuD
rfbE
metE
nrfD
ttrS
tagH
K21397
srlE
terC
sboE
ccmF
glnD
misL
glnE
E1.13.11.4
hybO
melB
sucA
bcsA
-Log10 (p-Value)
Log2 Fold Change

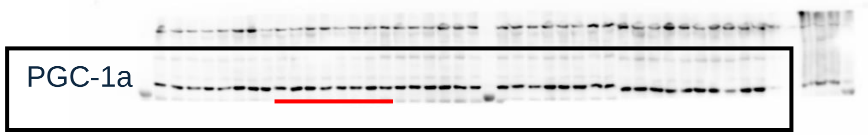

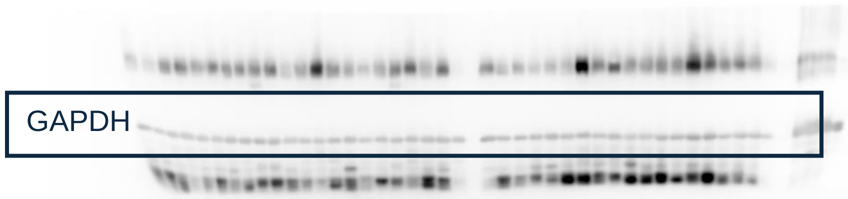

PGC-1α (figure 3 h)

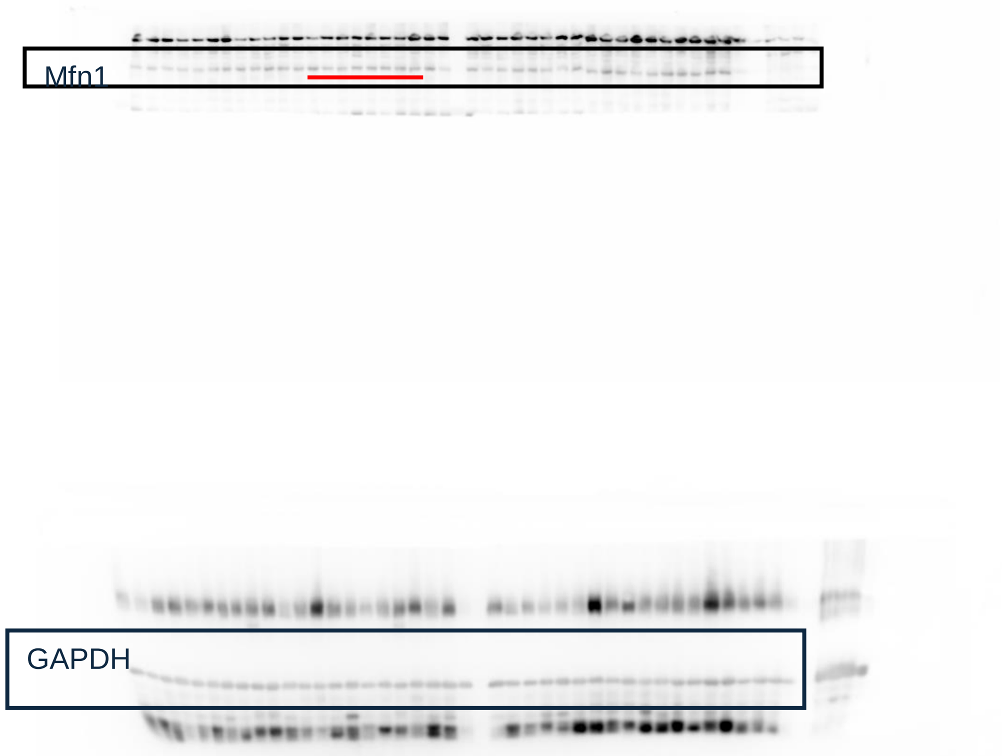

MFN1(figure 3 j)

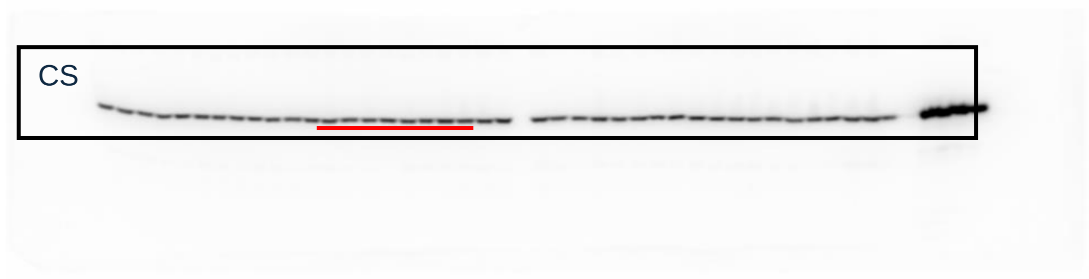

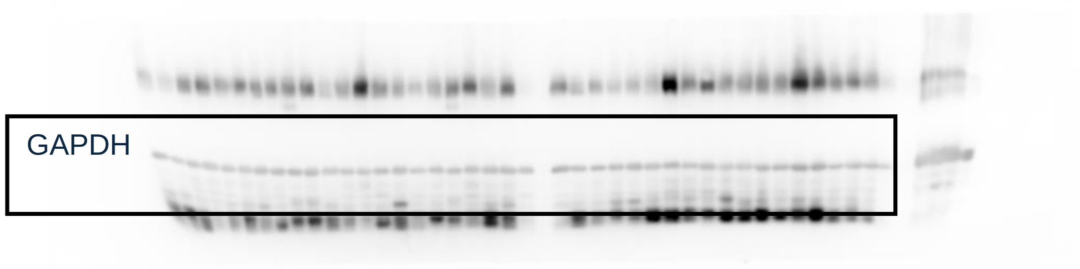

CS (figure 3 i)

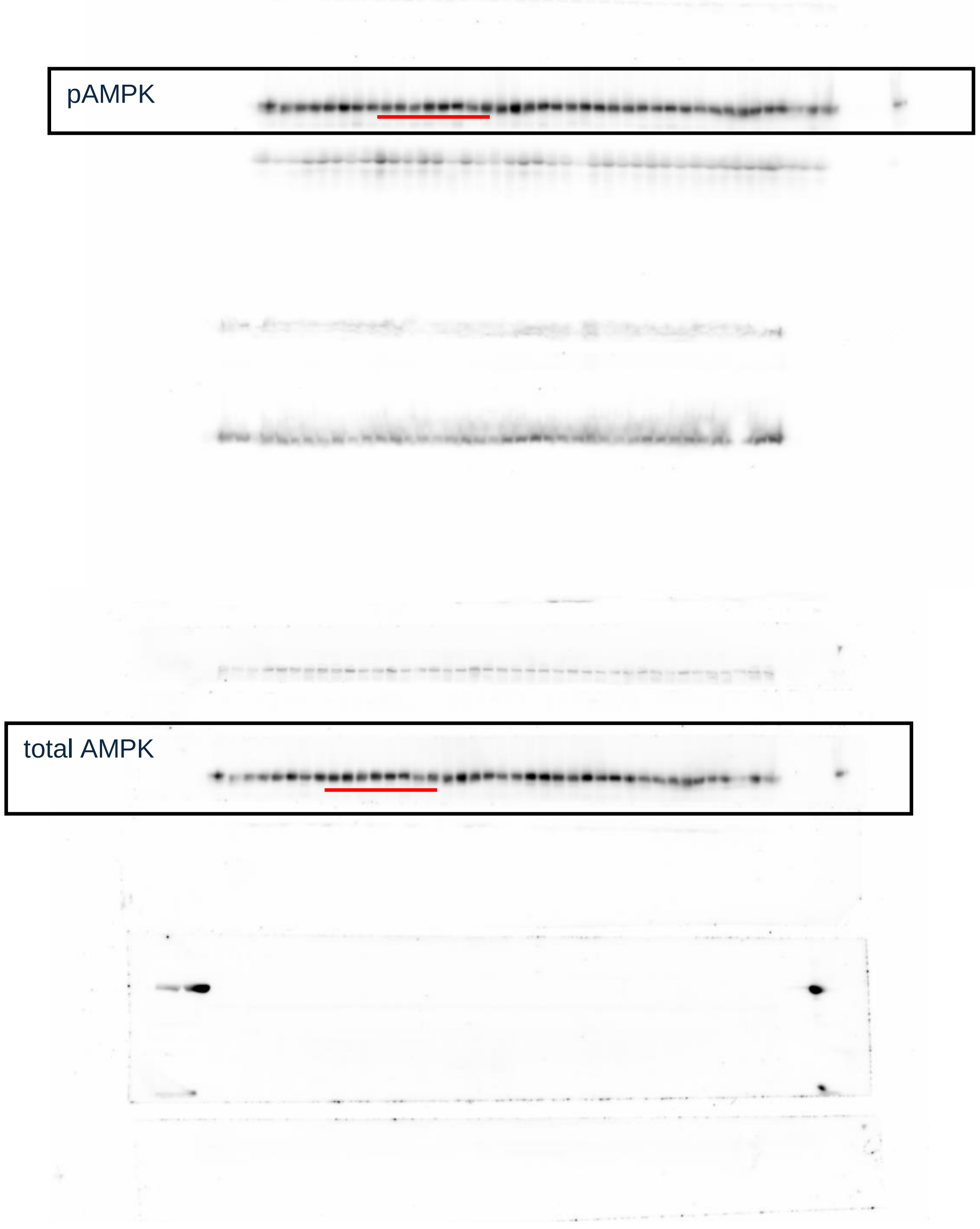

p/t AMPK (figure 3 a)

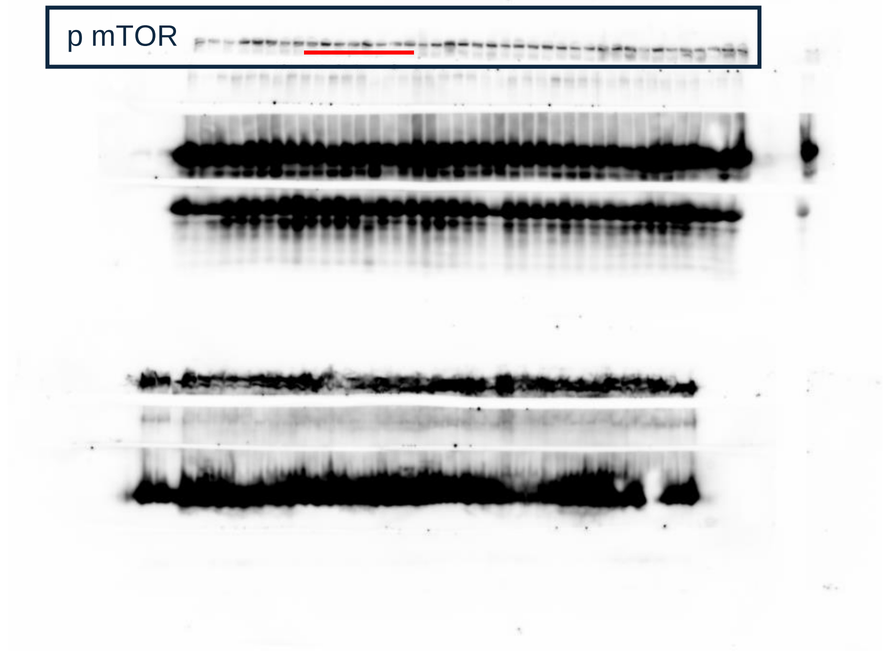

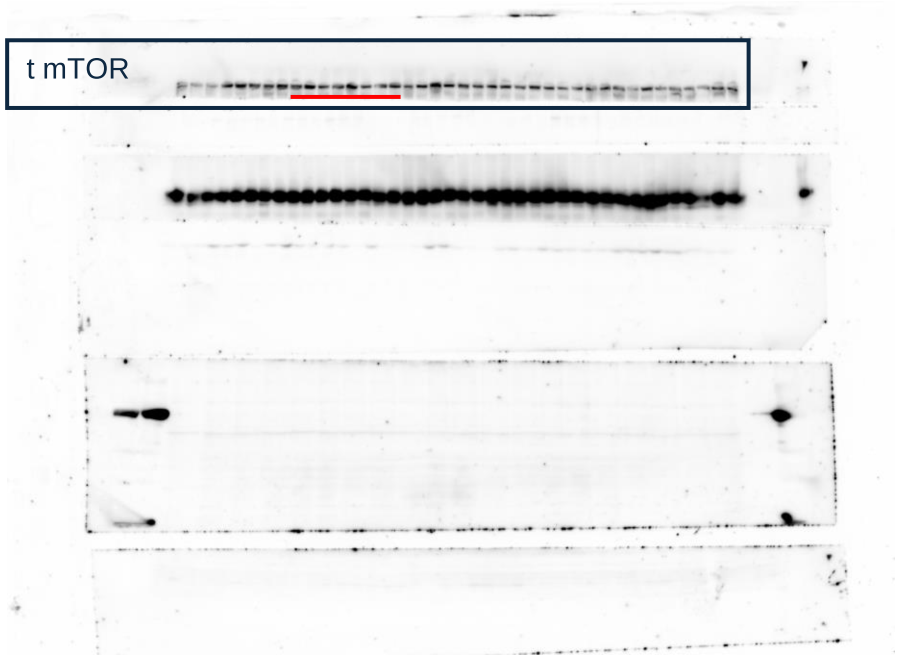

p/t mTOR (figure 3 c)

p/t CREB (figure 3 b)

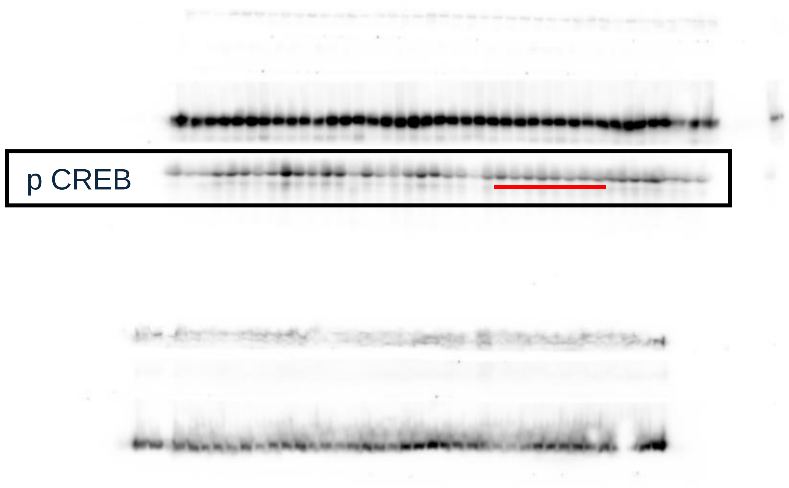

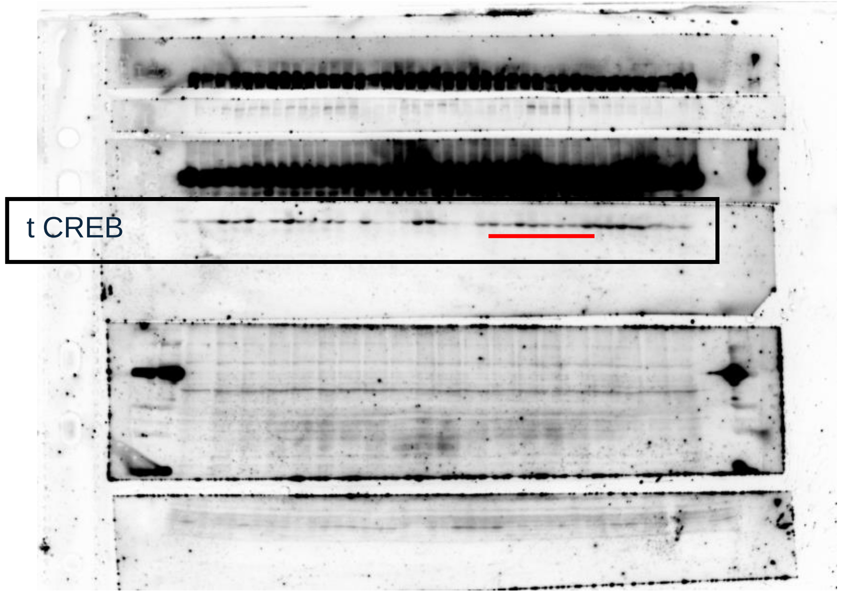

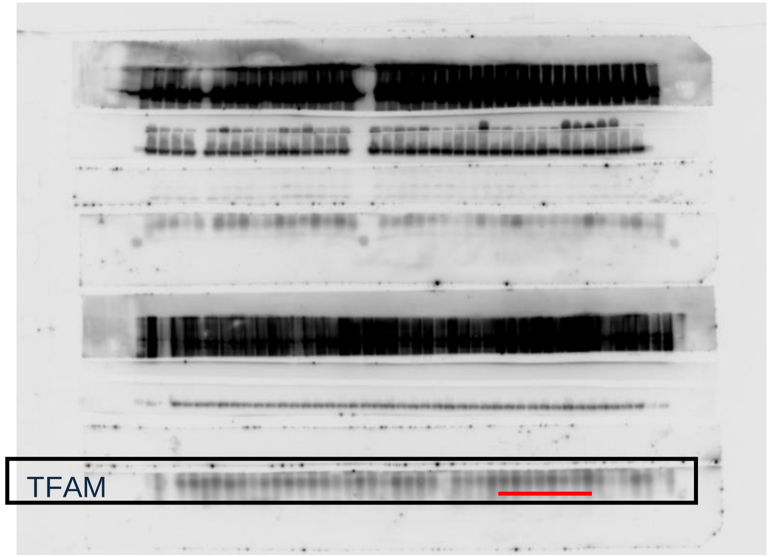

TFAM (figure 3 e)

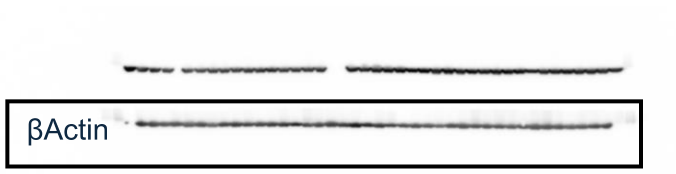

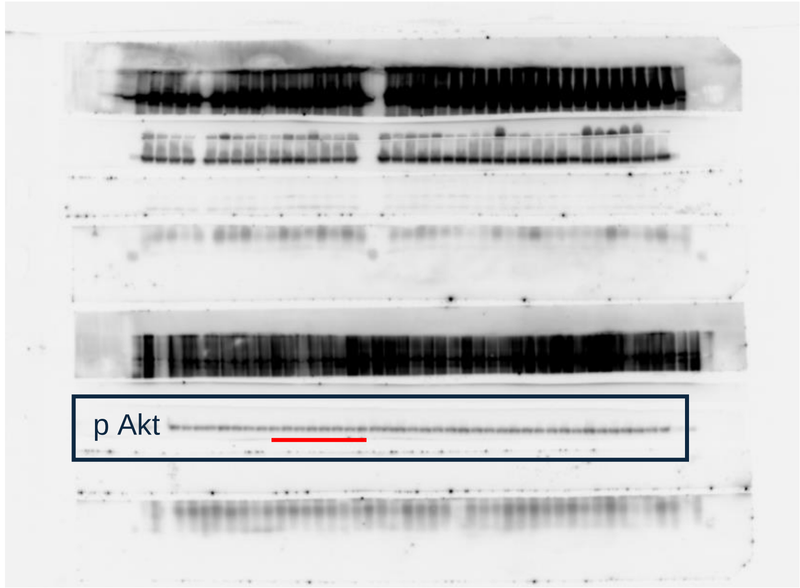

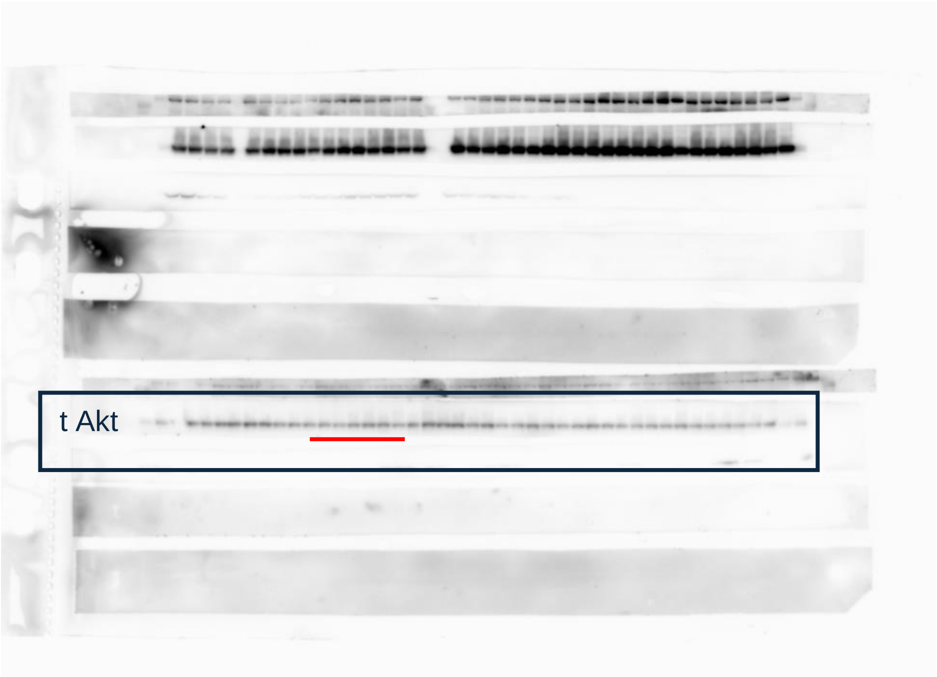

p/t AKT(figure 3 d)

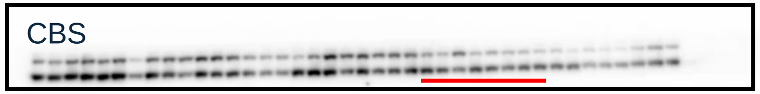

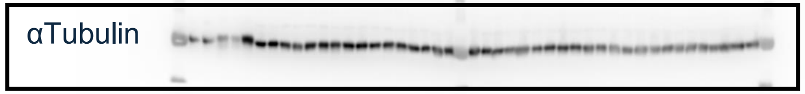

CBS (figure 3 f)

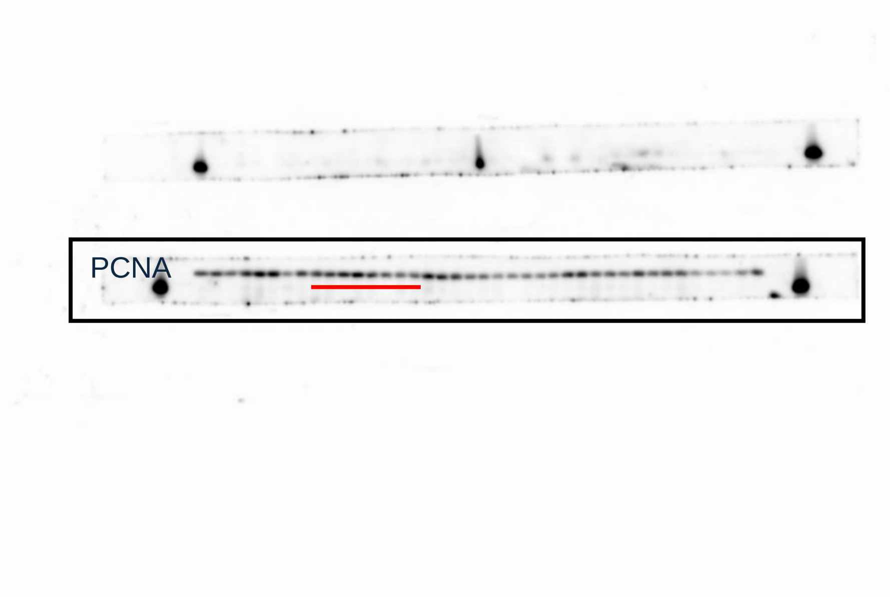

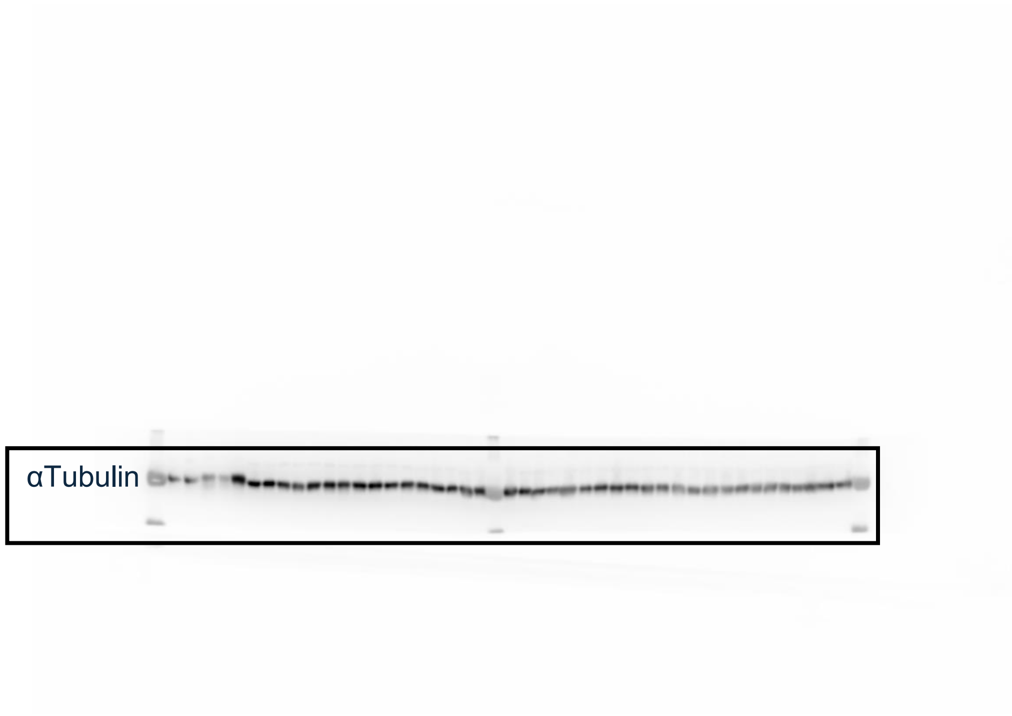

PCNA (figure 3 g)

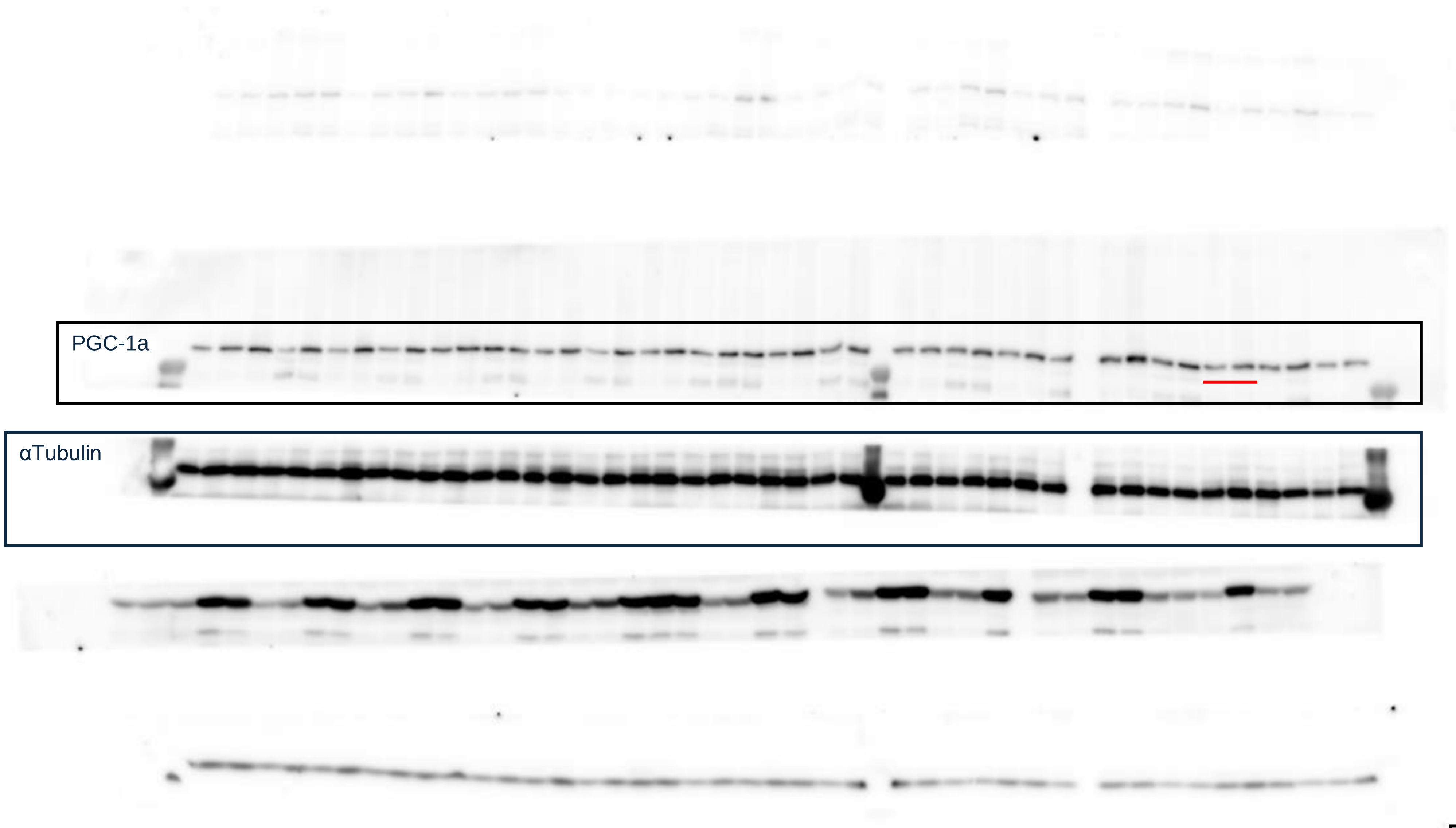

PGC-1α in skeletal muscle
