# Supplementary material for: PGC-1 alpha overexpression in the skeletal muscle results in a metabolically active microbiome which is independent of redox signaling
Source: Sci Rep. 2025 Jul 1;15:20527. doi: 10.1038/s41598-025-05594-w (PMC12215982; doi:10.1038/s41598-025-05594-w)
Supplement: Supplementary file 2 — Supplementary Material 2 [file 41598_2025_5594_MOESM2_ESM.html]

|  |  |  |  |
| --- | --- | --- | --- |
| Comparison groups | | | |
| at the Genus level | | | |
| Taxon | LogFC | P value | BH P |
| PGC-1α-Ex after vs. before | | | |
| --- | --- | --- | --- |
| Desulfovibrio | -0.505 | 1.73e-05 | 3.30e-02\* |
| Ligilactobacillus | -0.571 | 5.40e-05 | 3.98e-02\* |
| Turicimonas | 1.229 | 6.25e-05 | 3.98e-02\* |
| Macrococcus | -0.666 | 4.68e-04 | 1.85e-01 |
| Neptunomonas | 1.509 | 9.60e-04 | 2.62e-01 |
| Pasteurella | 0.854 | 1.74e-03 | 4.16e-01 |
| Pannonibacter | 0.762 | 2.39e-03 | 5.02e-01 |
| Bibersteinia | -3.190 | 2.89e-03 | 5.02e-01 |
| Salmonella | 0.662 | 3.56e-03 | 5.11e-01 |
| Borrelia | -2.384 | 3.71e-03 | 5.11e-01 |
| Francisella | -0.679 | 3.75e-03 | 5.11e-01 |
| Faecalibaculum | -1.611 | 5.00e-03 | 5.96e-01 |
| Candidatus Arthromitus | 1.414 | 6.51e-03 | 6.91e-01 |
| Alcanivorax | 0.812 | 6.94e-03 | 6.97e-01 |
| Tetrasphaera | 2.009 | 7.67e-03 | 6.98e-01 |
| Halobacterium | -2.430 | 8.79e-03 | 7.63e-01 |
| Halomonas | 0.700 | 1.28e-02 | 9.41e-01 |
| Cellulophaga | -0.563 | 1.32e-02 | 9.41e-01 |
| Dermatophilus | -1.207 | 1.55e-02 | 9.41e-01 |
| Candidatus Nucleicultrix | -1.626 | 1.59e-02 | 9.41e-01 |
| Tolypothrix | -2.144 | 1.66e-02 | 9.41e-01 |
| Geminocystis | -0.918 | 1.70e-02 | 9.41e-01 |
| Limosilactobacillus | -0.921 | 1.72e-02 | 9.41e-01 |
| Micrococcus | -0.667 | 1.73e-02 | 9.41e-01 |
| Methanoplanus | -4.164 | 1.77e-02 | 9.41e-01 |
| Ezakiella | 0.658 | 1.86e-02 | 9.61e-01 |
| Siansivirga | -1.727 | 2.13e-02 | 9.99e-01 |
| Demequina | -2.091 | 2.33e-02 | 9.99e-01 |
| Heyndrickxia | -0.634 | 2.37e-02 | 9.99e-01 |
| Blastochloris | 0.736 | 2.44e-02 | 9.99e-01 |
| Zhouia | -0.673 | 2.64e-02 | 9.99e-01 |
| Mycolicibacillus | -2.005 | 2.65e-02 | 9.99e-01 |
| Micropruina | -0.642 | 2.79e-02 | 9.99e-01 |
| Arcobacter | -0.536 | 2.80e-02 | 9.99e-01 |
| Synechocystis | -1.134 | 2.86e-02 | 9.99e-01 |
| Algibacter | -0.546 | 3.16e-02 | 9.99e-01 |
| Basfia | -1.557 | 3.24e-02 | 9.99e-01 |
| Mageeibacillus | -0.538 | 3.28e-02 | 9.99e-01 |
| Salinivibrio | -0.591 | 3.80e-02 | 9.99e-01 |
| Terribacillus | 0.608 | 3.89e-02 | 9.99e-01 |
| Natronobacterium | 1.054 | 3.90e-02 | 9.99e-01 |
| Coprococcus | -0.674 | 3.94e-02 | 9.99e-01 |
| Neotabrizicola | -0.783 | 4.05e-02 | 9.99e-01 |
| Exiguobacterium | 0.552 | 4.06e-02 | 9.99e-01 |
| Amphibacillus | -1.403 | 4.15e-02 | 9.99e-01 |
| Occallatibacter | 0.565 | 4.22e-02 | 9.99e-01 |
| Akkermansia | 0.970 | 4.80e-02 | 9.99e-01 |
| Bifidobacterium | 1.618 | 4.80e-02 | 9.99e-01 |
| Pontimonas | -1.869 | 4.84e-02 | 9.99e-01 |
| Amylolactobacillus | 2.256 | 4.91e-02 | 9.99e-01 |
| Thioclava | 0.581 | 4.95e-02 | 9.99e-01 |
| Wt-Ex after vs. before | | | |
| Mycobacterium | 0.610 | 2.77e-07 | 5.23e-04\* |
| Arcanobacterium | 0.761 | 2.46e-05 | 2.32e-02\* |
| Fluviispira | -3.410 | 7.79e-04 | 4.23e-01 |
| Exiguobacterium | -0.963 | 9.19e-04 | 4.23e-01 |
| Pasteurella | -0.972 | 1.31e-03 | 4.23e-01 |
| Bifidobacterium | 1.373 | 1.39e-03 | 4.23e-01 |
| Fructilactobacillus | 2.701 | 1.76e-03 | 4.23e-01 |
| Methanoplanus | -1.514 | 2.11e-03 | 4.23e-01 |
| Nodularia | 3.468 | 2.84e-03 | 4.23e-01 |
| Halogeometricum | 2.597 | 2.88e-03 | 4.23e-01 |
| Stanieria | 2.621 | 2.91e-03 | 4.23e-01 |
| Dermabacter | 0.992 | 3.47e-03 | 4.67e-01 |
| Halomonas | -0.962 | 4.32e-03 | 5.12e-01 |
| Pengzhenrongella | 1.251 | 4.34e-03 | 5.12e-01 |
| Mesoflavibacter | 3.899 | 5.35e-03 | 5.33e-01 |
| Mycolicibacillus | -1.725 | 5.57e-03 | 5.33e-01 |
| Alcanivorax | -1.053 | 5.58e-03 | 5.33e-01 |
| Paraglaciecola | -0.895 | 6.62e-03 | 5.65e-01 |
| Abiotrophia | 2.227 | 6.89e-03 | 5.65e-01 |
| Methylocella | -0.743 | 1.09e-02 | 8.57e-01 |
| Curtobacterium | 0.518 | 1.23e-02 | 9.26e-01 |
| Leptogranulimonas | 0.955 | 1.52e-02 | 1.00e+00 |
| Salmonella | -0.670 | 1.58e-02 | 1.00e+00 |
| Rugosibacter | -0.912 | 1.58e-02 | 1.00e+00 |
| Natrinema | -4.068 | 1.91e-02 | 1.00e+00 |
| Miniimonas | 2.705 | 2.07e-02 | 1.00e+00 |
| Akkermansia | 2.187 | 2.41e-02 | 1.00e+00 |
| Urechidicola | -0.748 | 2.61e-02 | 1.00e+00 |
| Phycicoccus | 0.575 | 2.84e-02 | 1.00e+00 |
| Acidianus | -0.913 | 3.14e-02 | 1.00e+00 |
| Ketogulonicigenium | -0.648 | 3.16e-02 | 1.00e+00 |
| Candidatus Westeberhardia | 2.875 | 3.35e-02 | 1.00e+00 |
| Candidatus Fukatsuia | 1.439 | 3.46e-02 | 1.00e+00 |
| Desulfolutivibrio | -0.570 | 3.76e-02 | 1.00e+00 |
| Anaerolinea | -0.748 | 3.86e-02 | 1.00e+00 |
| Sinomonas | 1.191 | 4.19e-02 | 1.00e+00 |
| Micropruina | 0.519 | 4.20e-02 | 1.00e+00 |
| Candidatus Hepatoplasma | 1.230 | 4.68e-02 | 1.00e+00 |
| Halobacteriovorax | -1.932 | 4.84e-02 | 1.00e+00 |
| Pacificitalea | -3.197 | 4.88e-02 | 1.00e+00 |
| PGC-1α-Ex before vs. Wt-Ex before | | | |
| Companilactobacillus | 1.503 | 2.31e-06 | 4.39e-03\* |
| Marinomonas | 1.014 | 3.26e-05 | 3.10e-02\* |
| Turicimonas | -2.342 | 6.12e-05 | 3.70e-02\* |
| Gracilibacillus | 0.795 | 1.04e-04 | 3.70e-02\* |
| Syntrophomonas | 1.045 | 1.11e-04 | 3.70e-02\* |
| Borrelia | 2.046 | 1.22e-04 | 3.70e-02\* |
| Cloacibacterium | 0.733 | 1.36e-04 | 3.70e-02\* |
| Methanobacterium | 4.206 | 1.72e-04 | 4.10e-02\* |
| Anoxybacillus | 0.634 | 2.53e-04 | 4.59e-02\* |
| Glutamicibacter | 0.557 | 2.53e-04 | 4.59e-02\* |
| Enterobacter | 1.729 | 2.71e-04 | 4.59e-02\* |
| Providencia | 0.588 | 2.90e-04 | 4.59e-02\* |
| Anabaena | 1.768 | 3.98e-04 | 5.44e-02 |
| Helicobacter | 0.521 | 4.53e-04 | 5.74e-02 |
| Aneurinibacillus | 0.724 | 5.22e-04 | 6.20e-02 |
| Methylocella | -0.881 | 5.97e-04 | 6.47e-02 |
| Muribaculum | -1.329 | 6.47e-04 | 6.47e-02 |
| Lachnoanaerobaculum | 0.620 | 7.53e-04 | 6.58e-02 |
| Jeotgalibaca | 0.721 | 7.85e-04 | 6.58e-02 |
| Mycobacterium | 0.924 | 7.96e-04 | 6.58e-02 |
| Leptogranulimonas | -1.907 | 1.11e-03 | 8.78e-02 |
| Neokomagataea | 3.635 | 1.15e-03 | 8.78e-02 |
| Hyphomonas | 0.543 | 1.28e-03 | 9.39e-02 |
| Syntrophothermus | -0.862 | 1.34e-03 | 9.40e-02 |
| Arcanobacterium | 0.715 | 1.56e-03 | 1.06e-01 |
| Methanococcoides | 1.871 | 1.75e-03 | 1.13e-01 |
| Croceicoccus | -0.920 | 1.82e-03 | 1.13e-01 |
| Ewingella | -1.212 | 1.85e-03 | 1.13e-01 |
| Rippkaea | 1.874 | 1.90e-03 | 1.13e-01 |
| Maridesulfovibrio | 0.542 | 1.97e-03 | 1.14e-01 |
| Acaryochloris | 0.909 | 2.20e-03 | 1.23e-01 |
| Brasilonema | 2.262 | 2.74e-03 | 1.49e-01 |
| Pseudomonas | -0.860 | 3.59e-03 | 1.74e-01 |
| Thiodictyon | -0.708 | 3.67e-03 | 1.74e-01 |
| Intestinibaculum | 0.730 | 3.68e-03 | 1.74e-01 |
| Sulfurivermis | 0.755 | 3.78e-03 | 1.74e-01 |
| Aerosticca | -0.813 | 3.85e-03 | 1.74e-01 |
| Macrococcus | 0.725 | 4.02e-03 | 1.77e-01 |
| Basfia | 1.184 | 4.15e-03 | 1.77e-01 |
| Gardnerella | 0.578 | 4.20e-03 | 1.77e-01 |
| Marinithermus | -1.140 | 4.68e-03 | 1.88e-01 |
| Pelosinus | 0.577 | 4.69e-03 | 1.88e-01 |
| Candidatus Saccharimonas | -1.351 | 4.85e-03 | 1.88e-01 |
| Vagococcus | 0.572 | 5.03e-03 | 1.89e-01 |
| Imtechella | 1.395 | 5.27e-03 | 1.91e-01 |
| Caldithrix | -0.753 | 5.33e-03 | 1.91e-01 |
| Paenisporosarcina | 2.385 | 5.84e-03 | 2.05e-01 |
| Laribacter | -0.682 | 5.99e-03 | 2.05e-01 |
| Urechidicola | -1.352 | 6.03e-03 | 2.05e-01 |
| Bacteroides | 0.782 | 6.31e-03 | 2.07e-01 |
| Synechocystis | 0.875 | 6.32e-03 | 2.07e-01 |
| Geminocystis | 1.031 | 6.74e-03 | 2.12e-01 |
| Singulisphaera | -0.838 | 6.84e-03 | 2.12e-01 |
| Limosilactobacillus | -1.061 | 7.26e-03 | 2.19e-01 |
| Methyloligella | -0.893 | 7.65e-03 | 2.19e-01 |
| Caminibacter | 1.454 | 7.67e-03 | 2.19e-01 |
| Pelagovum | -0.734 | 7.83e-03 | 2.19e-01 |
| Blastochloris | -0.680 | 7.84e-03 | 2.19e-01 |
| Thiopseudomonas | -1.236 | 8.22e-03 | 2.23e-01 |
| Stappia | -0.689 | 8.71e-03 | 2.33e-01 |
| Candidatus Vallotia | 0.949 | 9.25e-03 | 2.38e-01 |
| Ligilactobacillus | 0.544 | 9.45e-03 | 2.39e-01 |
| Algibacter | 0.650 | 9.61e-03 | 2.39e-01 |
| Dermabacter | 0.696 | 9.95e-03 | 2.39e-01 |
| Salinivibrio | 0.555 | 9.95e-03 | 2.39e-01 |
| Salmonella | -0.848 | 1.04e-02 | 2.46e-01 |
| Pasteurella | -0.730 | 1.12e-02 | 2.50e-01 |
| Faecalibaculum | 1.981 | 1.13e-02 | 2.50e-01 |
| Pannonibacter | -0.710 | 1.13e-02 | 2.50e-01 |
| Latilactobacillus | 0.773 | 1.15e-02 | 2.50e-01 |
| Exiguobacterium | -0.520 | 1.27e-02 | 2.71e-01 |
| Psychroflexus | 0.562 | 1.28e-02 | 2.71e-01 |
| Tetragenococcus | 0.711 | 1.33e-02 | 2.74e-01 |
| Verminephrobacter | -0.661 | 1.33e-02 | 2.74e-01 |
| Candidatus Nucleicultrix | 1.125 | 1.34e-02 | 2.74e-01 |
| Candidatus Deianiraea | 1.005 | 1.35e-02 | 2.74e-01 |
| Cobetia | 0.528 | 1.42e-02 | 2.81e-01 |
| Ureaplasma | 3.997 | 1.53e-02 | 2.90e-01 |
| Coprococcus | 1.082 | 1.55e-02 | 2.90e-01 |
| Meiothermus | -0.681 | 1.61e-02 | 2.90e-01 |
| Occallatibacter | -0.616 | 1.62e-02 | 2.90e-01 |
| Neotabrizicola | 0.748 | 1.63e-02 | 2.90e-01 |
| Limnobaculum | -2.406 | 1.64e-02 | 2.90e-01 |
| Thiomicrospira | 0.630 | 1.65e-02 | 2.90e-01 |
| Hyphococcus | 1.303 | 1.65e-02 | 2.90e-01 |
| Ferribacterium | -0.512 | 1.73e-02 | 2.96e-01 |
| Sulfidibacter | -0.528 | 1.85e-02 | 3.13e-01 |
| Gallibacterium | 0.708 | 1.87e-02 | 3.13e-01 |
| Lawsonella | 0.540 | 1.89e-02 | 3.13e-01 |
| Aggregatibacter | 0.592 | 1.89e-02 | 3.13e-01 |
| Croceibacterium | 2.188 | 1.92e-02 | 3.14e-01 |
| Litorivicinus | 0.778 | 2.00e-02 | 3.16e-01 |
| Fictibacillus | 0.524 | 2.00e-02 | 3.16e-01 |
| Porphyrobacter | -0.545 | 2.01e-02 | 3.16e-01 |
| Lacunisphaera | -0.582 | 2.08e-02 | 3.20e-01 |
| Sulfuriflexus | 2.654 | 2.11e-02 | 3.20e-01 |
| Pelobacter | 0.514 | 2.15e-02 | 3.20e-01 |
| Sulfuricella | 1.212 | 2.18e-02 | 3.20e-01 |
| Alcanivorax | -0.727 | 2.22e-02 | 3.20e-01 |
| Agrococcus | -0.747 | 2.25e-02 | 3.20e-01 |
| Cylindrospermopsis | 3.963 | 2.25e-02 | 3.20e-01 |
| Nitrobacter | -2.088 | 2.25e-02 | 3.20e-01 |
| Mesoflavibacter | 2.547 | 2.30e-02 | 3.24e-01 |
| Mycetohabitans | 2.686 | 2.33e-02 | 3.24e-01 |
| Thalassotalea | 1.067 | 2.36e-02 | 3.24e-01 |
| Acidiferrobacter | -0.551 | 2.38e-02 | 3.24e-01 |
| Amphibacillus | 1.181 | 2.40e-02 | 3.24e-01 |
| Thermococcus | 0.731 | 2.41e-02 | 3.24e-01 |
| Melioribacter | 0.690 | 2.42e-02 | 3.24e-01 |
| Halomonas | -0.641 | 2.47e-02 | 3.26e-01 |
| Halobaculum | -1.345 | 2.47e-02 | 3.26e-01 |
| Thioclava | -0.703 | 2.54e-02 | 3.26e-01 |
| Zhouia | 0.563 | 2.57e-02 | 3.26e-01 |
| Mageeibacillus | 0.746 | 2.62e-02 | 3.26e-01 |
| Dinoroseobacter | -0.535 | 2.69e-02 | 3.26e-01 |
| Chitinibacter | 1.179 | 2.69e-02 | 3.26e-01 |
| Yinghuangia | 0.552 | 2.70e-02 | 3.26e-01 |
| Loigolactobacillus | 0.553 | 2.73e-02 | 3.26e-01 |
| Enterococcus | 0.526 | 2.74e-02 | 3.26e-01 |
| Lonsdalea | -0.648 | 2.75e-02 | 3.26e-01 |
| Rhodovastum | 0.538 | 2.77e-02 | 3.26e-01 |
| Scytonema | 0.773 | 2.89e-02 | 3.31e-01 |
| Faecalitalea | -0.559 | 2.93e-02 | 3.31e-01 |
| Tichowtungia | -0.510 | 2.98e-02 | 3.35e-01 |
| Tahibacter | -0.647 | 3.05e-02 | 3.41e-01 |
| Saccharibacillus | 0.537 | 3.17e-02 | 3.46e-01 |
| Desulfomonile | 0.870 | 3.20e-02 | 3.46e-01 |
| Basilea | 1.509 | 3.22e-02 | 3.46e-01 |
| Candidatus Arthromitus | -1.349 | 3.24e-02 | 3.46e-01 |
| Candidatus Amoebophilus | 1.047 | 3.28e-02 | 3.47e-01 |
| Koinonema | 1.544 | 3.32e-02 | 3.47e-01 |
| Candidatus Mycosynbacter | -1.601 | 3.34e-02 | 3.47e-01 |
| Psychroserpens | 0.756 | 3.37e-02 | 3.47e-01 |
| Breoghania | -0.741 | 3.38e-02 | 3.47e-01 |
| Wujia | 0.546 | 3.38e-02 | 3.47e-01 |
| Solicola | 3.625 | 3.54e-02 | 3.57e-01 |
| Allobaculum | -0.989 | 3.55e-02 | 3.57e-01 |
| Rugosibacter | -0.947 | 3.60e-02 | 3.60e-01 |
| Prescottella | -0.830 | 3.65e-02 | 3.64e-01 |
| Heliorestis | 0.600 | 3.81e-02 | 3.75e-01 |
| Hydrogenobacter | -0.652 | 3.88e-02 | 3.80e-01 |
| Syntrophobotulus | 0.597 | 3.93e-02 | 3.83e-01 |
| Psychrobacter | 0.960 | 4.13e-02 | 3.97e-01 |
| Saccharomonospora | 0.543 | 4.23e-02 | 4.02e-01 |
| Catenovulum | 3.160 | 4.29e-02 | 4.02e-01 |
| Kalamiella | -0.793 | 4.30e-02 | 4.02e-01 |
| Cylindrospermum | 3.590 | 4.38e-02 | 4.07e-01 |
| Gulosibacter | 0.530 | 4.41e-02 | 4.07e-01 |
| Candidatus Contubernalis | 0.557 | 4.45e-02 | 4.07e-01 |
| Paradesulfovibrio (ex Waite et al. 2020) | -0.631 | 4.55e-02 | 4.12e-01 |
| Asaia | -0.511 | 4.63e-02 | 4.17e-01 |
| Bibersteinia | 1.622 | 4.74e-02 | 4.24e-01 |
| Amylolactobacillus | -1.614 | 4.79e-02 | 4.24e-01 |
| Halovivax | -1.497 | 4.95e-02 | 4.27e-01 |
| Oceanithermus | -0.543 | 4.97e-02 | 4.27e-01 |
| PGC-1α-Ex after vs. Wt-Ex after | | | |
| Bacteroides | 1.008 | 1.14e-16 | 2.17e-13\* |
| Micropruina | -0.928 | 9.95e-08 | 9.46e-05\* |
| Limosilactobacillus | -1.973 | 2.69e-07 | 1.70e-04\* |
| Paraglaciecola | 0.986 | 5.90e-06 | 2.80e-03\* |
| Phycicoccus | -1.302 | 1.60e-05 | 5.27e-03\* |
| Aeromicrobium | -0.578 | 1.86e-05 | 5.27e-03\* |
| Adlercreutzia | -0.623 | 1.94e-05 | 5.27e-03\* |
| Anaerolinea | 1.103 | 5.02e-05 | 1.19e-02\* |
| Dermacoccus | -0.904 | 5.65e-05 | 1.19e-02\* |
| Niastella | 0.629 | 1.50e-04 | 2.85e-02\* |
| Exiguobacterium | 0.856 | 2.50e-04 | 3.96e-02\* |
| Cellulomonas | -0.514 | 3.97e-04 | 5.80e-02 |
| Lactobacillus | -1.679 | 7.00e-04 | 7.94e-02 |
| Pasteurella | 0.889 | 7.43e-04 | 7.94e-02 |
| Muribaculum | -0.872 | 7.78e-04 | 7.94e-02 |
| Arsenicicoccus | -0.899 | 7.93e-04 | 7.94e-02 |
| Salmonella | 0.742 | 1.29e-03 | 1.22e-01 |
| Enterobacter | 0.562 | 1.56e-03 | 1.37e-01 |
| Ketogulonicigenium | 0.661 | 1.66e-03 | 1.37e-01 |
| Frateuria | -0.629 | 2.00e-03 | 1.52e-01 |
| Actinotignum | -0.961 | 2.99e-03 | 2.11e-01 |
| Halogeometricum | -2.210 | 3.62e-03 | 2.19e-01 |
| Aliarcobacter | -0.542 | 3.86e-03 | 2.19e-01 |
| Hyphomicrobium | -0.525 | 3.91e-03 | 2.19e-01 |
| Parabacteroides | 1.093 | 4.68e-03 | 2.54e-01 |
| Limnobaculum | -3.274 | 4.84e-03 | 2.56e-01 |
| Sulfurihydrogenibium | -3.014 | 5.28e-03 | 2.64e-01 |
| Halomonas | 0.796 | 5.58e-03 | 2.68e-01 |
| Candidatus Cardinium | -4.040 | 5.63e-03 | 2.68e-01 |
| Parazoarcus | 0.550 | 7.88e-03 | 3.36e-01 |
| Bifidobacterium | -2.122 | 8.22e-03 | 3.36e-01 |
| Colwellia | 0.734 | 8.58e-03 | 3.36e-01 |
| Alcanivorax | 0.828 | 9.16e-03 | 3.48e-01 |
| Parvibaculum | 0.675 | 9.78e-03 | 3.51e-01 |
| Afipia | -0.817 | 9.98e-03 | 3.51e-01 |
| Acholeplasma | -0.801 | 1.19e-02 | 3.82e-01 |
| Coprococcus | 0.726 | 1.39e-02 | 4.19e-01 |
| Kibdelosporangium | -0.605 | 1.48e-02 | 4.33e-01 |
| Sanguibacter | -1.665 | 1.51e-02 | 4.34e-01 |
| Chitinibacter | 1.746 | 1.63e-02 | 4.44e-01 |
| Syntrophothermus | -0.563 | 1.72e-02 | 4.44e-01 |
| Labrys | -1.137 | 1.77e-02 | 4.44e-01 |
| Aerosticca | -0.688 | 1.77e-02 | 4.44e-01 |
| Caldichromatium | -0.830 | 1.80e-02 | 4.45e-01 |
| Halosimplex | -1.599 | 1.88e-02 | 4.47e-01 |
| Micrococcus | -0.527 | 1.97e-02 | 4.51e-01 |
| Defluviicoccus | -0.795 | 1.99e-02 | 4.51e-01 |
| Flagellimonas | 0.640 | 2.10e-02 | 4.51e-01 |
| Propioniciclava | -0.509 | 2.10e-02 | 4.51e-01 |
| Verminephrobacter | -0.641 | 2.20e-02 | 4.51e-01 |
| Lapidilactobacillus | -3.268 | 2.20e-02 | 4.51e-01 |
| Lacinutrix | -0.585 | 2.26e-02 | 4.57e-01 |
| Escherichia | -0.689 | 2.38e-02 | 4.67e-01 |
| Staphylococcus | 0.567 | 2.44e-02 | 4.67e-01 |
| Haploplasma | -1.150 | 2.46e-02 | 4.67e-01 |
| Marinomonas | 0.552 | 2.65e-02 | 4.76e-01 |
| Gloeomargarita | 0.530 | 2.69e-02 | 4.76e-01 |
| Paraneptunicella | 2.349 | 2.71e-02 | 4.76e-01 |
| Paraprevotella | 0.553 | 2.73e-02 | 4.76e-01 |
| Parasaccharibacter | 0.596 | 2.79e-02 | 4.78e-01 |
| Methanoplanus | -2.599 | 2.92e-02 | 4.91e-01 |
| Aestuariispira | 0.745 | 2.97e-02 | 4.96e-01 |
| Mariniplasma | -1.006 | 3.18e-02 | 5.26e-01 |
| Methyloceanibacter | 0.621 | 3.35e-02 | 5.49e-01 |
| Candidatus Hydrogenosomobacter | 0.745 | 3.54e-02 | 5.65e-01 |
| Neptunomonas | 0.794 | 3.57e-02 | 5.66e-01 |
| Avibacterium | -0.943 | 3.63e-02 | 5.70e-01 |
| Urechidicola | -1.106 | 3.92e-02 | 5.93e-01 |
| Roseimaritima | -0.554 | 3.96e-02 | 5.93e-01 |
| Beutenbergia | -2.171 | 3.96e-02 | 5.93e-01 |
| Gephyromycinifex | -0.534 | 4.24e-02 | 5.99e-01 |
| Leeuwenhoekiella | -1.284 | 4.34e-02 | 5.99e-01 |
| Actibacterium | -0.941 | 4.40e-02 | 5.99e-01 |
| Paeniglutamicibacter | 0.950 | 4.41e-02 | 5.99e-01 |
| Methylacidiphilum | 0.599 | 4.51e-02 | 5.99e-01 |
| Microbacter | -0.604 | 4.57e-02 | 5.99e-01 |
| Natronobacterium | 0.732 | 4.58e-02 | 5.99e-01 |
| Cylindrospermum | 3.674 | 4.63e-02 | 5.99e-01 |
| Methylovorus | 4.077 | 4.66e-02 | 5.99e-01 |
| Symbiopectobacterium | -1.286 | 4.73e-02 | 5.99e-01 |
| Yimella | -2.047 | 4.80e-02 | 6.00e-01 |
| Citromicrobium | -3.652 | 4.91e-02 | 6.09e-01 |
| PGC-1α-C after vs. Wt-C after | | | |
| Exiguobacterium | 0.634 | 1.59e-15 | 3.06e-12\* |
| Halomonas | 0.674 | 3.53e-13 | 3.40e-10\* |
| Alcanivorax | 0.686 | 2.74e-10 | 1.76e-07\* |
| Lacinutrix | -1.079 | 7.07e-07 | 3.41e-04\* |
| Xiamenia | -0.669 | 1.55e-06 | 5.43e-04\* |
| Raoultibacter | -0.585 | 2.51e-06 | 6.77e-04\* |
| Sulfuritalea | 1.145 | 2.81e-06 | 6.77e-04\* |
| Arabiibacter | -0.714 | 4.41e-06 | 8.32e-04\* |
| Riemerella | 1.008 | 4.61e-06 | 8.32e-04\* |
| Gordonibacter | -0.680 | 4.75e-06 | 8.32e-04\* |
| Ligilactobacillus | -1.539 | 5.27e-06 | 8.46e-04\* |
| Eggerthella | -0.584 | 1.09e-05 | 1.62e-03\* |
| Paraglaciecola | 0.683 | 1.21e-05 | 1.67e-03\* |
| Berryella | -0.618 | 1.53e-05 | 1.89e-03\* |
| Chitinolyticbacter | 1.219 | 1.57e-05 | 1.89e-03\* |
| Francisella | -0.737 | 3.61e-05 | 4.09e-03\* |
| Arcanobacterium | -0.795 | 4.82e-05 | 5.16e-03\* |
| Pasteurella | 0.553 | 5.53e-05 | 5.61e-03\* |
| Leptogranulimonas | -2.660 | 1.50e-04 | 1.29e-02\* |
| Leptotrichia | -0.609 | 1.54e-04 | 1.29e-02\* |
| Frischella | -2.541 | 1.80e-04 | 1.39e-02\* |
| Belliella | 0.893 | 2.62e-04 | 1.94e-02\* |
| Acetoanaerobium | -0.803 | 3.06e-04 | 2.19e-02\* |
| Lonsdalea | 0.921 | 3.27e-04 | 2.24e-02\* |
| Pyruvatibacter | 0.705 | 3.37e-04 | 2.24e-02\* |
| Tyzzerella | -0.523 | 3.49e-04 | 2.24e-02\* |
| Adlercreutzia | -0.672 | 4.52e-04 | 2.67e-02\* |
| Sulfuriferula | 0.557 | 4.61e-04 | 2.67e-02\* |
| Yinghuangia | 0.924 | 4.84e-04 | 2.67e-02\* |
| Catenibacterium | -0.576 | 4.85e-04 | 2.67e-02\* |
| Paludibacter | 0.524 | 5.46e-04 | 2.84e-02\* |
| Halothermothrix | -0.937 | 6.19e-04 | 3.14e-02\* |
| Gloeocapsa | 1.025 | 6.48e-04 | 3.20e-02\* |
| Proteus | 0.700 | 6.90e-04 | 3.20e-02\* |
| Vagococcus | -0.607 | 6.98e-04 | 3.20e-02\* |
| Pseudocnuella | 0.564 | 7.04e-04 | 3.20e-02\* |
| Salmonella | 0.753 | 7.14e-04 | 3.20e-02\* |
| Paeniclostridium | -0.737 | 7.79e-04 | 3.36e-02\* |
| Sodalis | -0.662 | 7.85e-04 | 3.36e-02\* |
| Ornithobacterium | -1.559 | 9.16e-04 | 3.68e-02\* |
| Malaciobacter | -0.987 | 1.07e-03 | 4.06e-02\* |
| Cruoricaptor | 1.040 | 1.08e-03 | 4.06e-02\* |
| Aquibium | 0.760 | 1.14e-03 | 4.24e-02\* |
| Mycolicibacillus | -1.595 | 1.17e-03 | 4.24e-02\* |
| Macrococcus | -0.668 | 1.32e-03 | 4.63e-02\* |
| Thermophilibacter | -1.399 | 1.33e-03 | 4.63e-02\* |
| Stieleria | 0.623 | 1.38e-03 | 4.68e-02\* |
| Prosthecochloris | 0.544 | 1.43e-03 | 4.74e-02\* |
| Lysobacter | 0.524 | 1.54e-03 | 4.96e-02\* |
| Tellurirhabdus | 0.568 | 1.62e-03 | 5.03e-02 |
| Methanohalophilus | -3.131 | 1.77e-03 | 5.17e-02 |
| Undibacterium | 0.968 | 1.78e-03 | 5.17e-02 |
| Superficieibacter | 0.959 | 1.80e-03 | 5.17e-02 |
| Parolsenella | -1.522 | 1.84e-03 | 5.22e-02 |
| Candidatus Azobacteroides | 0.573 | 1.87e-03 | 5.24e-02 |
| Enterobacter | 0.607 | 1.93e-03 | 5.30e-02 |
| Dehalobacter | -0.580 | 2.06e-03 | 5.60e-02 |
| Serpentinicella | -0.657 | 2.29e-03 | 6.03e-02 |
| Olsenella | -1.156 | 2.60e-03 | 6.77e-02 |
| Tepidiforma | 0.667 | 2.78e-03 | 7.04e-02 |
| Kingella | 0.671 | 2.83e-03 | 7.04e-02 |
| Mariniflexile | 0.714 | 2.93e-03 | 7.07e-02 |
| Lawsonia | -0.916 | 2.97e-03 | 7.07e-02 |
| Mongoliitalea | -0.775 | 3.29e-03 | 7.61e-02 |
| Paraburkholderia | 0.816 | 3.32e-03 | 7.61e-02 |
| Intestinibaculum | -0.595 | 3.49e-03 | 7.73e-02 |
| Parvularcula | -0.808 | 3.59e-03 | 7.76e-02 |
| Sarcina | -0.689 | 3.61e-03 | 7.76e-02 |
| Paracoccus | 0.547 | 3.63e-03 | 7.76e-02 |
| Caloranaerobacter | -0.769 | 3.81e-03 | 7.92e-02 |
| Thermovirga | -2.121 | 3.83e-03 | 7.92e-02 |
| Aminobacter | 0.835 | 3.86e-03 | 7.92e-02 |
| Kurthia | -0.900 | 4.01e-03 | 8.03e-02 |
| Ancylobacter | 0.594 | 4.06e-03 | 8.03e-02 |
| Acididesulfobacillus | -0.581 | 4.18e-03 | 8.14e-02 |
| Rufibacter | 0.535 | 4.44e-03 | 8.41e-02 |
| Melissococcus | -1.273 | 4.53e-03 | 8.41e-02 |
| Candidatus Saccharimonas | -1.716 | 4.55e-03 | 8.41e-02 |
| Paraclostridium | -0.644 | 4.67e-03 | 8.49e-02 |
| Oenococcus | -0.744 | 4.76e-03 | 8.50e-02 |
| Burkholderia | 0.741 | 4.79e-03 | 8.50e-02 |
| Glycocaulis | -0.648 | 4.90e-03 | 8.50e-02 |
| Maribellus | 0.741 | 5.16e-03 | 8.88e-02 |
| Phoenicibacter | -0.800 | 5.40e-03 | 9.18e-02 |
| Acholeplasma | -0.852 | 5.93e-03 | 9.60e-02 |
| Parafannyhessea | -1.060 | 6.16e-03 | 9.73e-02 |
| Carboxydocella | -0.508 | 6.32e-03 | 9.83e-02 |
| Aquiflexum | 0.807 | 6.33e-03 | 9.83e-02 |
| Terrisporobacter | -0.727 | 6.43e-03 | 9.84e-02 |
| Sphingosinithalassobacter | 0.547 | 6.49e-03 | 9.84e-02 |
| Mammaliicoccus | -0.530 | 6.54e-03 | 9.84e-02 |
| Caldimicrobium | -2.433 | 6.63e-03 | 9.90e-02 |
| Coxiella | 0.762 | 6.76e-03 | 9.92e-02 |
| Salinivibrio | -0.596 | 6.87e-03 | 9.92e-02 |
| Emticicia | -0.637 | 6.90e-03 | 9.92e-02 |
| Pseudopedobacter | -0.722 | 7.11e-03 | 1.02e-01 |
| Companilactobacillus | -0.973 | 7.16e-03 | 1.02e-01 |
| Mycetocola | 0.566 | 7.25e-03 | 1.02e-01 |
| Muribaculum | 0.768 | 7.57e-03 | 1.05e-01 |
| Congregibacter | 0.614 | 7.61e-03 | 1.05e-01 |
| Chloroflexus | 0.786 | 8.06e-03 | 1.10e-01 |
| Natronobacterium | 1.615 | 8.17e-03 | 1.10e-01 |
| Methylomicrobium | 0.751 | 8.49e-03 | 1.13e-01 |
| Algibacter | -0.592 | 8.71e-03 | 1.15e-01 |
| Oscillatoria | 0.587 | 8.93e-03 | 1.17e-01 |
| Synechocystis | -0.786 | 9.05e-03 | 1.17e-01 |
| Collinsella | -0.629 | 9.09e-03 | 1.17e-01 |
| Heyndrickxia | -0.585 | 9.17e-03 | 1.17e-01 |
| Zophobihabitans | -3.853 | 9.20e-03 | 1.17e-01 |
| Chelatococcus | 0.513 | 9.80e-03 | 1.22e-01 |
| Fluviispira | -2.176 | 1.00e-02 | 1.23e-01 |
| Marinagarivorans | -1.300 | 1.01e-02 | 1.23e-01 |
| Paenisporosarcina | -1.234 | 1.02e-02 | 1.23e-01 |
| Candidatus Hydrogenosomobacter | 1.473 | 1.07e-02 | 1.26e-01 |
| Haliscomenobacter | 0.686 | 1.08e-02 | 1.27e-01 |
| Alkalitalea | 0.603 | 1.16e-02 | 1.33e-01 |
| Ciceribacter | 0.803 | 1.17e-02 | 1.33e-01 |
| Kaistia | 0.838 | 1.20e-02 | 1.36e-01 |
| Candidatus Blochmannia | 0.767 | 1.22e-02 | 1.38e-01 |
| Frigoriglobus | 0.512 | 1.25e-02 | 1.39e-01 |
| Chryseolinea | 0.565 | 1.27e-02 | 1.39e-01 |
| Cohaesibacter | 0.819 | 1.33e-02 | 1.42e-01 |
| Tepidibacter | -0.564 | 1.37e-02 | 1.46e-01 |
| Sediminicoccus | 0.707 | 1.40e-02 | 1.46e-01 |
| Methylosinus | 0.782 | 1.41e-02 | 1.46e-01 |
| Methylocella | 0.604 | 1.44e-02 | 1.48e-01 |
| Methylovirgula | 0.786 | 1.53e-02 | 1.54e-01 |
| Paraprevotella | 0.573 | 1.55e-02 | 1.55e-01 |
| Peptacetobacter | -0.556 | 1.57e-02 | 1.56e-01 |
| Candidatus Amoebophilus | -1.290 | 1.67e-02 | 1.62e-01 |
| Hahella | -0.525 | 1.70e-02 | 1.63e-01 |
| Faecalibacter | -1.709 | 1.70e-02 | 1.63e-01 |
| Halobacteriovorax | -1.612 | 1.79e-02 | 1.67e-01 |
| Tannockella | -0.565 | 1.81e-02 | 1.68e-01 |
| Demequina | -0.903 | 1.83e-02 | 1.69e-01 |
| Nautilia | 1.009 | 1.89e-02 | 1.73e-01 |
| Solicola | -4.023 | 2.05e-02 | 1.83e-01 |
| Butyricimonas | 0.593 | 2.06e-02 | 1.83e-01 |
| Fuscovulum | 0.543 | 2.07e-02 | 1.83e-01 |
| Serpentinimonas | 0.582 | 2.09e-02 | 1.83e-01 |
| Parasedimentitalea | -1.374 | 2.10e-02 | 1.83e-01 |
| Fluviicola | 0.754 | 2.13e-02 | 1.84e-01 |
| Petrotoga | -0.710 | 2.18e-02 | 1.87e-01 |
| Apilactobacillus | -0.932 | 2.25e-02 | 1.89e-01 |
| Ideonella | -1.866 | 2.34e-02 | 1.95e-01 |
| Ferriphaselus | -1.043 | 2.44e-02 | 2.01e-01 |
| Wolinella | 0.620 | 2.44e-02 | 2.01e-01 |
| Halorhabdus | 1.026 | 2.50e-02 | 2.03e-01 |
| Segniliparus | -2.666 | 2.52e-02 | 2.03e-01 |
| Variibacter | 0.684 | 2.52e-02 | 2.03e-01 |
| Psychroflexus | -0.561 | 2.53e-02 | 2.03e-01 |
| Ureaplasma | -1.814 | 2.56e-02 | 2.04e-01 |
| Candidatus Contubernalis | -0.584 | 2.67e-02 | 2.11e-01 |
| Richelia | -2.292 | 2.70e-02 | 2.12e-01 |
| Vaginimicrobium | -0.934 | 2.79e-02 | 2.15e-01 |
| Ureibacillus | -0.534 | 2.82e-02 | 2.15e-01 |
| Desulfurobacterium | -1.488 | 2.84e-02 | 2.15e-01 |
| Agarilytica | 0.663 | 2.84e-02 | 2.15e-01 |
| Cetobacterium | -0.705 | 2.86e-02 | 2.15e-01 |
| Halarcobacter | -0.768 | 3.15e-02 | 2.26e-01 |
| Rhodovastum | -0.557 | 3.19e-02 | 2.27e-01 |
| Methylophaga | -1.039 | 3.21e-02 | 2.27e-01 |
| Georhizobium | 0.566 | 3.37e-02 | 2.36e-01 |
| Caldisericum | -2.549 | 3.42e-02 | 2.38e-01 |
| Methanomicrobium | 1.040 | 3.49e-02 | 2.41e-01 |
| Bacterioplanes | -1.642 | 3.58e-02 | 2.44e-01 |
| Alistipes | 0.947 | 3.89e-02 | 2.57e-01 |
| Photorhabdus | -0.648 | 3.91e-02 | 2.58e-01 |
| Rodentibacter | -0.745 | 4.05e-02 | 2.63e-01 |
| Mycoplasma | -1.201 | 4.06e-02 | 2.63e-01 |
| Gilliamella | -0.790 | 4.09e-02 | 2.64e-01 |
| Austwickia | 0.537 | 4.15e-02 | 2.68e-01 |
| Candidatus Portiera | -3.261 | 4.27e-02 | 2.70e-01 |
| Mycoplasmopsis | -0.524 | 4.28e-02 | 2.70e-01 |
| Candidatus Kuenenia | -0.716 | 4.28e-02 | 2.70e-01 |
| Kosmotoga | -1.063 | 4.30e-02 | 2.70e-01 |
| Amylolactobacillus | 1.004 | 4.31e-02 | 2.70e-01 |
| Thiospirochaeta | -0.712 | 4.33e-02 | 2.70e-01 |
| Sideroxyarcus | 0.817 | 4.35e-02 | 2.70e-01 |
| Terasakiella | 0.561 | 4.36e-02 | 2.70e-01 |
| Anaplasma | -1.346 | 4.53e-02 | 2.76e-01 |
| Glaciecola | 0.646 | 4.65e-02 | 2.82e-01 |
| Coriobacterium | -0.521 | 4.69e-02 | 2.82e-01 |
| Alkalilimnicola | 0.617 | 4.70e-02 | 2.82e-01 |
| Peptostreptococcus | -0.550 | 4.72e-02 | 2.82e-01 |
