# Supplementary material for: PGC-1 alpha overexpression in the skeletal muscle results in a metabolically active microbiome which is independent of redox signaling
Source: Sci Rep. 2025 Jul 1;15:20527. doi: 10.1038/s41598-025-05594-w (PMC12215982; doi:10.1038/s41598-025-05594-w)
Supplement: Supplementary file 3 — Supplementary Material 3 [file 41598_2025_5594_MOESM3_ESM.html]

|  |  |  |  |
| --- | --- | --- | --- |
| Comparison groups | | | |
| by biological function | | | |
| ID | logFC | P value | BH P |
| PGC-1α-Ex after vs. before | | | |
| --- | --- | --- | --- |
| urdA; urocanate reductase [EC:1.3.99.33] | 0.576 | 9.73e-11 | 3.33e-07\* |
| TC.DCUC, dcuC, dcuD; C4-dicarboxylate transporter, DcuC family | 0.897 | 9.99e-09 | 2.28e-05\* |
| K07074; uncharacterized protein | -0.827 | 2.41e-08 | 4.13e-05\* |
| per, rfbE; perosamine synthetase [EC:2.6.1.102] | -0.602 | 7.27e-08 | 9.21e-05\* |
| tagH; teichoic acid transport system ATP-binding protein [EC:7.5.2.4] | -0.557 | 2.06e-07 | 2.02e-04\* |
| metE; 5-methyltetrahydropteroyltriglutamate--homocysteine methyltransferase [EC:2.1.1.14] | 0.755 | 4.35e-07 | 3.72e-04\* |
| nrfD; protein NrfD | 0.945 | 7.16e-07 | 4.90e-04\* |
| ttrS; two-component system, LuxR family, sensor histidine kinase TtrS [EC:2.7.13.3] | 1.540 | 1.51e-06 | 9.40e-04\* |
| terC; tellurite resistance protein TerC | 3.100 | 9.05e-06 | 4.77e-03\* |
| nukE, mcdE, sboE; lantibiotic transport system permease protein | -0.591 | 1.49e-05 | 6.90e-03\* |
| K21397; ABC transport system ATP-binding/permease protein | 1.476 | 1.51e-05 | 6.90e-03\* |
| srlE; glucitol/sorbitol PTS system EIIB component [EC:2.7.1.198] | 1.246 | 3.03e-05 | 1.30e-02\* |
| E1.13.11.4; gentisate 1,2-dioxygenase [EC:1.13.11.4] | 2.989 | 3.63e-05 | 1.46e-02\* |
| glnD; [protein-PII] uridylyltransferase [EC:2.7.7.59] | 2.376 | 4.30e-05 | 1.64e-02\* |
| ccmF; cytochrome c-type biogenesis protein CcmF | 0.886 | 5.70e-05 | 2.05e-02\* |
| aidA-I, misL; autotransporter family porin | 0.914 | 8.67e-05 | 2.66e-02\* |
| bcsA; cellulose synthase (UDP-forming) [EC:2.4.1.12] | 0.940 | 8.91e-05 | 2.66e-02\* |
| OGDH, sucA; 2-oxoglutarate dehydrogenase E1 component [EC:1.2.4.2] | 1.090 | 1.37e-04 | 3.75e-02\* |
| hyaA, hybO; hydrogenase small subunit [EC:1.12.99.6] | 0.823 | 1.52e-04 | 4.01e-02\* |
| glnE; [glutamine synthetase] adenylyltransferase / [glutamine synthetase]-adenylyl-L-tyrosine phosphorylase [EC:2.7.7.42 2.7.7.89] | 1.631 | 1.59e-04 | 4.04e-02\* |
| melB; melibiose permease | -0.641 | 2.00e-04 | 4.73e-02\* |
| ybbN; putative thioredoxin | 4.112 | 2.31e-04 | 5.11e-02 |
| algD; GDP-mannose 6-dehydrogenase [EC:1.1.1.132] | 1.010 | 2.51e-04 | 5.37e-02 |
| add, ADA; adenosine deaminase [EC:3.5.4.4] | 1.335 | 2.68e-04 | 5.57e-02 |
| ssuA; sulfonate transport system substrate-binding protein | 1.166 | 3.10e-04 | 6.18e-02 |
| yejB; microcin C transport system permease protein | 2.322 | 3.16e-04 | 6.18e-02 |
| hrpA; ATP-dependent helicase HrpA [EC:3.6.4.13] | 1.636 | 4.30e-04 | 7.73e-02 |
| ydhQ; GntR family transcriptional regulator | -1.009 | 4.51e-04 | 7.73e-02 |
| lgaB; L-galactono-1,5-lactonase [EC:3.1.1.-] | -0.533 | 5.99e-04 | 9.88e-02 |
| fctD; glutamate formiminotransferase / 5-formyltetrahydrofolate cyclo-ligase [EC:2.1.2.5 6.3.3.2] | -0.611 | 6.17e-04 | 9.88e-02 |
| bmaC; fibronectin-binding autotransporter adhesin | 1.643 | 6.20e-04 | 9.88e-02 |
| pepE; dipeptidase E [EC:3.4.13.21] | 0.699 | 8.61e-04 | 1.28e-01 |
| NIT1, ybeM; deaminated glutathione amidase [EC:3.5.1.128] | 2.189 | 1.05e-03 | 1.49e-01 |
| ripA; peptidoglycan DL-endopeptidase RipA [EC:3.4.-.-] | 2.522 | 1.14e-03 | 1.53e-01 |
| susR; HTH-type transcriptional regulator, polysaccharide utilization system transcription regulator | -1.362 | 1.19e-03 | 1.57e-01 |
| E1.1.1.67, mtlK; mannitol 2-dehydrogenase [EC:1.1.1.67] | -1.004 | 1.35e-03 | 1.71e-01 |
| GMPP; mannose-1-phosphate guanylyltransferase [EC:2.7.7.13] | 0.921 | 1.38e-03 | 1.71e-01 |
| nrfC; protein NrfC | 0.827 | 1.53e-03 | 1.87e-01 |
| cpg; glutamate carboxypeptidase [EC:3.4.17.11] | 0.732 | 1.74e-03 | 1.95e-01 |
| ppk2; polyphosphate kinase [EC:2.7.4.1] | 0.987 | 1.75e-03 | 1.95e-01 |
| yqgT; g-D-glutamyl-meso-diaminopimelate peptidase [EC:3.4.19.11] | -0.871 | 1.76e-03 | 1.95e-01 |
| slyB; outer membrane lipoprotein SlyB | 1.393 | 1.83e-03 | 1.99e-01 |
| traN; conjugal transfer mating pair stabilization protein TraN | 1.270 | 1.87e-03 | 2.00e-01 |
| srlD; sorbitol-6-phosphate 2-dehydrogenase [EC:1.1.1.140] | 0.813 | 1.94e-03 | 2.01e-01 |
| phsA, psrA; thiosulfate reductase / polysulfide reductase chain A [EC:1.8.5.5] | 0.665 | 2.25e-03 | 2.20e-01 |
| yaeR; glyoxylase I family protein | -0.923 | 2.31e-03 | 2.23e-01 |
| recC; exodeoxyribonuclease V gamma subunit [EC:3.1.11.5] | 1.441 | 2.43e-03 | 2.31e-01 |
| baiH; 7beta-hydroxy-3-oxochol-24-oyl-CoA 4-desaturase [EC:1.3.1.116] | 2.282 | 2.50e-03 | 2.31e-01 |
| agrA, blpR, fsrA; two-component system, LytTR family, response regulator AgrA | -1.136 | 2.72e-03 | 2.48e-01 |
| ureI; acid-activated urea channel | -1.044 | 2.86e-03 | 2.53e-01 |
| K09787; uncharacterized protein | -0.600 | 3.04e-03 | 2.56e-01 |
| caiA; crotonobetainyl-CoA dehydrogenase [EC:1.3.8.13] | -1.250 | 3.21e-03 | 2.56e-01 |
| pilD, pppA; leader peptidase (prepilin peptidase) / N-methyltransferase [EC:3.4.23.43 2.1.1.-] | 1.932 | 3.25e-03 | 2.56e-01 |
| rne; ribonuclease E [EC:3.1.26.12] | 1.210 | 3.27e-03 | 2.56e-01 |
| mscK, kefA, aefA; potassium-dependent mechanosensitive channel | 0.921 | 3.32e-03 | 2.56e-01 |
| flhB2; flagellar biosynthesis protein | -0.582 | 3.34e-03 | 2.56e-01 |
| amgK; N-acetylmuramate 1-kinase [EC:2.7.1.221] | 0.568 | 3.37e-03 | 2.56e-01 |
| bacC; dihydroanticapsin dehydrogenase [EC:1.1.1.385] | 0.773 | 3.43e-03 | 2.56e-01 |
| pabA; para-aminobenzoate synthetase component II [EC:2.6.1.85] | 2.570 | 3.44e-03 | 2.56e-01 |
| gatB, sgcB; galactitol PTS system EIIB component [EC:2.7.1.200] | 1.376 | 3.51e-03 | 2.56e-01 |
| metB; cystathionine gamma-synthase [EC:2.5.1.48] | 1.448 | 3.54e-03 | 2.56e-01 |
| pldB; lysophospholipase [EC:3.1.1.5] | 3.258 | 3.55e-03 | 2.56e-01 |
| abgA; aminobenzoyl-glutamate utilization protein A | 0.724 | 3.73e-03 | 2.66e-01 |
| peb1A, glnH; aspartate/glutamate/glutamine transport system substrate-binding protein | -0.933 | 3.81e-03 | 2.68e-01 |
| IAL; isopenicillin-N N-acyltransferase like protein | 2.522 | 4.45e-03 | 3.02e-01 |
| xtmA; phage terminase small subunit | -0.661 | 4.78e-03 | 3.18e-01 |
| sucD; succinyl-CoA synthetase alpha subunit [EC:6.2.1.5] | 1.010 | 4.97e-03 | 3.20e-01 |
| pdxS, pdx1; pyridoxal 5'-phosphate synthase pdxS subunit [EC:4.3.3.6] | 0.627 | 5.02e-03 | 3.20e-01 |
| hepA; heparin lyase [EC:4.2.2.7] | -1.120 | 5.09e-03 | 3.20e-01 |
| licR; lichenan operon transcriptional antiterminator | -1.251 | 5.46e-03 | 3.37e-01 |
| peb1B, glnP, glnM; aspartate/glutamate/glutamine transport system permease protein | -0.854 | 5.84e-03 | 3.55e-01 |
| prlC; oligopeptidase A [EC:3.4.24.70] | 0.975 | 5.85e-03 | 3.55e-01 |
| bcr, tcaB; MFS transporter, DHA1 family, multidrug resistance protein | 1.041 | 6.27e-03 | 3.63e-01 |
| hcxA; hydroxycarboxylate dehydrogenase A [EC:1.1.1.-] | 2.349 | 6.31e-03 | 3.63e-01 |
| narL; two-component system, NarL family, nitrate/nitrite response regulator NarL | 2.580 | 6.34e-03 | 3.63e-01 |
| mpl; UDP-N-acetylmuramate: L-alanyl-gamma-D-glutamyl-meso-diaminopimelate ligase [EC:6.3.2.45] | 0.534 | 6.50e-03 | 3.63e-01 |
| moeA; molybdopterin molybdotransferase [EC:2.10.1.1] | 0.773 | 6.53e-03 | 3.63e-01 |
| mleA, mleS; malolactic enzyme [EC:4.1.1.101] | -0.889 | 6.53e-03 | 3.63e-01 |
| dotB, traJ; defect in organelle trafficking protein DotB [EC:7.2.4.8] | -2.221 | 6.54e-03 | 3.63e-01 |
| recB; exodeoxyribonuclease V beta subunit [EC:3.1.11.5] | 1.269 | 6.58e-03 | 3.63e-01 |
| dapD; 2,3,4,5-tetrahydropyridine-2,6-dicarboxylate N-succinyltransferase [EC:2.3.1.117] | 0.672 | 6.66e-03 | 3.63e-01 |
| yciA; acyl-CoA thioesterase YciA [EC:3.1.2.-] | 2.824 | 6.75e-03 | 3.63e-01 |
| ada; AraC family transcriptional regulator, regulatory protein of adaptative response / methylated-DNA-[protein]-cysteine methyltransferase [EC:2.1.1.63] | 1.375 | 6.83e-03 | 3.63e-01 |
| sat, met3; sulfate adenylyltransferase [EC:2.7.7.4] | -1.503 | 7.42e-03 | 3.79e-01 |
| gluA; glutamate transport system ATP-binding protein [EC:7.4.2.1] | 3.457 | 7.47e-03 | 3.79e-01 |
| srlA; glucitol/sorbitol PTS system EIIC component | 0.903 | 7.65e-03 | 3.85e-01 |
| cysI; sulfite reductase (NADPH) hemoprotein beta-component [EC:1.8.1.2] | 2.271 | 7.88e-03 | 3.86e-01 |
| fdxA; ferredoxin | 0.516 | 7.92e-03 | 3.86e-01 |
| rfbF; glucose-1-phosphate cytidylyltransferase [EC:2.7.7.33] | -0.533 | 7.93e-03 | 3.86e-01 |
| ydjE; MFS transporter, putative metabolite:H+ symporter | 1.756 | 7.94e-03 | 3.86e-01 |
| phnE; phosphonate transport system permease protein | -0.992 | 8.30e-03 | 3.95e-01 |
| srtA; sortase A [EC:3.4.22.70] | 0.791 | 8.31e-03 | 3.95e-01 |
| glgM; alpha-maltose-1-phosphate synthase [EC:2.4.1.342] | 3.135 | 8.94e-03 | 4.22e-01 |
| dgaC; D-glucosaminate PTS system EIIC component | 2.057 | 9.17e-03 | 4.23e-01 |
| spoVFB; dipicolinate synthase subunit B | -0.519 | 9.17e-03 | 4.23e-01 |
| SLC9B1\_2; solute carrier family 9B (sodium/hydrogen exchanger), member 1/2 | 1.245 | 9.33e-03 | 4.23e-01 |
| prpC, phpP; PPM family protein phosphatase [EC:3.1.3.16] | 0.793 | 9.36e-03 | 4.23e-01 |
| thiF; sulfur carrier protein ThiS adenylyltransferase [EC:2.7.7.73] | 1.061 | 9.39e-03 | 4.23e-01 |
| TRM61, GCD14; tRNA (adenine57-N1/adenine58-N1)-methyltransferase catalytic subunit [EC:2.1.1.219 2.1.1.220] | 2.788 | 9.64e-03 | 4.25e-01 |
| folE2; GTP cyclohydrolase IB [EC:3.5.4.16] | 0.934 | 9.81e-03 | 4.25e-01 |
| resE; two-component system, OmpR family, sensor histidine kinase ResE [EC:2.7.13.3] | 1.664 | 9.89e-03 | 4.25e-01 |
| patA, rscA, lmrC, satA; ATP-binding cassette, subfamily B, multidrug efflux pump | -1.029 | 9.96e-03 | 4.25e-01 |
| PDHB, pdhB; pyruvate dehydrogenase E1 component beta subunit [EC:1.2.4.1] | -0.830 | 9.99e-03 | 4.25e-01 |
| TREH, treA, treF; alpha,alpha-trehalase [EC:3.2.1.28] | -1.082 | 9.99e-03 | 4.25e-01 |
| levG; fructose PTS system EIID component | 0.688 | 1.03e-02 | 4.33e-01 |
| dadA; D-amino-acid dehydrogenase [EC:1.4.5.1] | 2.492 | 1.04e-02 | 4.33e-01 |
| kdgT; 2-keto-3-deoxygluconate permease | -1.443 | 1.04e-02 | 4.33e-01 |
| K06885; uncharacterized protein | -0.548 | 1.05e-02 | 4.33e-01 |
| fdhB; formate dehydrogenase (NADP+) beta subunit [EC:1.17.1.10] | 1.023 | 1.05e-02 | 4.33e-01 |
| toa; taurine---2-oxoglutarate transaminase [EC:2.6.1.55] | 1.324 | 1.06e-02 | 4.35e-01 |
| yajO; 1-deoxyxylulose-5-phosphate synthase [EC:1.1.-.-] | 0.504 | 1.07e-02 | 4.36e-01 |
| ulaE, sgaU, sgbU; L-ribulose-5-phosphate 3-epimerase [EC:5.1.3.22] | -0.585 | 1.11e-02 | 4.47e-01 |
| E1.2.1.68; coniferyl-aldehyde dehydrogenase [EC:1.2.1.68] | 2.663 | 1.15e-02 | 4.60e-01 |
| sbcB, exoI; exodeoxyribonuclease I [EC:3.1.11.1] | 1.501 | 1.16e-02 | 4.63e-01 |
| ttrR; two-component system, LuxR family, response regulator TtrR | 1.176 | 1.21e-02 | 4.77e-01 |
| pbuX; xanthine permease | -0.697 | 1.23e-02 | 4.77e-01 |
| TC.DASS; divalent anion:Na+ symporter, DASS family | 0.725 | 1.23e-02 | 4.77e-01 |
| tsr; methyl-accepting chemotaxis protein I, serine sensor receptor | -2.232 | 1.24e-02 | 4.77e-01 |
| tpa; taurine-pyruvate aminotransferase [EC:2.6.1.77] | -1.478 | 1.24e-02 | 4.77e-01 |
| lptD, imp, ostA; LPS-assembly protein | 1.129 | 1.32e-02 | 5.02e-01 |
| E1.11.1.5; cytochrome c peroxidase [EC:1.11.1.5] | 1.127 | 1.33e-02 | 5.02e-01 |
| fdhF; formate dehydrogenase (acceptor) [EC:1.17.99.7] | -0.912 | 1.35e-02 | 5.02e-01 |
| lplT; MFS transporter, LPLT family, lysophospholipid transporter | 1.040 | 1.36e-02 | 5.02e-01 |
| ecfA; energy-coupling factor transport system ATP-binding protein [EC:3.6.3.-] | 0.764 | 1.36e-02 | 5.02e-01 |
| torZ; trimethylamine-N-oxide reductase (cytochrome c) [EC:1.7.2.3] | 0.635 | 1.41e-02 | 5.14e-01 |
| dcuB; anaerobic C4-dicarboxylate transporter DcuB | 0.531 | 1.42e-02 | 5.14e-01 |
| mtnE, mtnV; L-glutamine---4-(methylsulfanyl)-2-oxobutanoate aminotransferase [EC:2.6.1.117] | 2.526 | 1.48e-02 | 5.25e-01 |
| bcsB; cellulose synthase operon protein B | 0.878 | 1.48e-02 | 5.25e-01 |
| lgaC; L-galactonate 5-dehydrogenase [EC:1.1.1.414] | -0.518 | 1.50e-02 | 5.30e-01 |
| K06946; uncharacterized protein | -1.378 | 1.51e-02 | 5.31e-01 |
| TC.GBP; general bacterial porin, GBP family | 0.716 | 1.58e-02 | 5.40e-01 |
| sucC; succinyl-CoA synthetase beta subunit [EC:6.2.1.5] | 1.334 | 1.60e-02 | 5.40e-01 |
| fwdE, fmdE; formylmethanofuran dehydrogenase subunit E [EC:1.2.7.12] | 1.370 | 1.62e-02 | 5.40e-01 |
| acoB; acetoin:2,6-dichlorophenolindophenol oxidoreductase subunit beta [EC:1.1.1.-] | 1.298 | 1.63e-02 | 5.40e-01 |
| ssp; subtilase-type serine protease [EC:3.4.21.-] | 1.992 | 1.64e-02 | 5.40e-01 |
| K07496; putative transposase | -0.819 | 1.65e-02 | 5.40e-01 |
| mop; aldehyde oxidoreductase [EC:1.2.99.7] | 0.533 | 1.67e-02 | 5.40e-01 |
| menC; O-succinylbenzoate synthase [EC:4.2.1.113] | 0.674 | 1.67e-02 | 5.40e-01 |
| SGSH; N-sulfoglucosamine sulfohydrolase [EC:3.10.1.1] | -1.194 | 1.69e-02 | 5.40e-01 |
| rlmE, rrmJ, ftsJ; 23S rRNA (uridine2552-2'-O)-methyltransferase [EC:2.1.1.166] | 1.032 | 1.82e-02 | 5.67e-01 |
| orn, REX2, REXO2; oligoribonuclease [EC:3.1.-.-] | 1.196 | 1.83e-02 | 5.67e-01 |
| E1.1.1.65; pyridoxine 4-dehydrogenase [EC:1.1.1.65] | 2.792 | 1.84e-02 | 5.67e-01 |
| tarL; CDP-ribitol ribitolphosphotransferase / teichoic acid ribitol-phosphate polymerase [EC:2.7.8.14 2.7.8.47] | -1.107 | 1.89e-02 | 5.78e-01 |
| mdcB; triphosphoribosyl-dephospho-CoA synthase [EC:2.4.2.52] | 1.762 | 1.92e-02 | 5.80e-01 |
| ssuC; sulfonate transport system permease protein | 1.351 | 1.93e-02 | 5.80e-01 |
| odh; opine dehydrogenase [EC:1.5.1.28] | 0.857 | 1.98e-02 | 5.90e-01 |
| ctpE; cation-transporting P-type ATPase E [EC:7.2.2.-] | 0.743 | 2.01e-02 | 5.94e-01 |
| acpS; holo-[acyl-carrier protein] synthase [EC:2.7.8.7] | 1.589 | 2.01e-02 | 5.94e-01 |
| narX; two-component system, NarL family, nitrate/nitrite sensor histidine kinase NarX [EC:2.7.13.3] | 2.396 | 2.04e-02 | 5.95e-01 |
| ccrM; modification methylase [EC:2.1.1.72] | 2.072 | 2.07e-02 | 5.98e-01 |
| ispA; farnesyl diphosphate synthase [EC:2.5.1.1 2.5.1.10] | 1.031 | 2.07e-02 | 5.98e-01 |
| ereA\_B; erythromycin esterase [EC:3.1.1.-] | -1.245 | 2.11e-02 | 6.07e-01 |
| iolC; 5-dehydro-2-deoxygluconokinase [EC:2.7.1.92] | 0.674 | 2.12e-02 | 6.07e-01 |
| hyaB, hybC; hydrogenase large subunit [EC:1.12.99.6] | 0.553 | 2.13e-02 | 6.07e-01 |
| psuK; pseudouridine kinase [EC:2.7.1.83] | -0.580 | 2.13e-02 | 6.07e-01 |
| alsE; D-allulose-6-phosphate 3-epimerase [EC:5.1.3.-] | 1.737 | 2.16e-02 | 6.11e-01 |
| gfrC; fructoselysine/glucoselysine PTS system EIIC component | 1.110 | 2.19e-02 | 6.14e-01 |
| mapP; maltose 6'-phosphate phosphatase [EC:3.1.3.90] | -2.515 | 2.20e-02 | 6.14e-01 |
| oprB; porin | 2.933 | 2.24e-02 | 6.23e-01 |
| nfnB, nfsB; nitroreductase / dihydropteridine reductase [EC:1.-.-.- 1.5.1.34] | 1.970 | 2.26e-02 | 6.23e-01 |
| tam; trans-aconitate 2-methyltransferase [EC:2.1.1.144] | -1.237 | 2.27e-02 | 6.23e-01 |
| patA; aminotransferase [EC:2.6.1.-] | -0.958 | 2.28e-02 | 6.24e-01 |
| K07177; Lon-like protease | 1.918 | 2.31e-02 | 6.28e-01 |
| zraS, hydH; two-component system, NtrC family, sensor histidine kinase HydH [EC:2.7.13.3] | -1.904 | 2.36e-02 | 6.37e-01 |
| degQ, hhoA; serine protease DegQ [EC:3.4.21.-] | -0.724 | 2.39e-02 | 6.40e-01 |
| dsrB; dissimilatory sulfite reductase beta subunit [EC:1.8.99.5] | -1.292 | 2.42e-02 | 6.42e-01 |
| pdxT, pdx2; 5'-phosphate synthase pdxT subunit [EC:4.3.3.6] | 1.126 | 2.43e-02 | 6.42e-01 |
| npr; NADH peroxidase [EC:1.11.1.1] | -0.724 | 2.45e-02 | 6.44e-01 |
| aphA; kanamycin kinase [EC:2.7.1.95] | 2.369 | 2.56e-02 | 6.63e-01 |
| rstA1; phage replication initiation protein | 0.562 | 2.59e-02 | 6.63e-01 |
| ompU; outer membrane protein OmpU | 2.556 | 2.59e-02 | 6.63e-01 |
| flhF; flagellar biosynthesis protein FlhF | -0.524 | 2.64e-02 | 6.70e-01 |
| K07097; uncharacterized protein | -0.853 | 2.65e-02 | 6.70e-01 |
| adiA; arginine decarboxylase [EC:4.1.1.19] | 1.330 | 2.67e-02 | 6.72e-01 |
| lysP; lysine-specific permease | -0.620 | 2.71e-02 | 6.79e-01 |
| DLST, sucB; 2-oxoglutarate dehydrogenase E2 component (dihydrolipoamide succinyltransferase) [EC:2.3.1.61] | 0.612 | 2.74e-02 | 6.80e-01 |
| gmhC, hldE, waaE, rfaE; D-beta-D-heptose 7-phosphate kinase / D-beta-D-heptose 1-phosphate adenosyltransferase [EC:2.7.1.167 2.7.7.70] | 0.739 | 2.76e-02 | 6.81e-01 |
| gntU; Gnt-I system low-affinity gluconate transporter | 0.617 | 2.76e-02 | 6.81e-01 |
| gatD; lipid II isoglutaminyl synthase (glutamine-hydrolysing) [EC:6.3.5.13] | 0.879 | 2.77e-02 | 6.81e-01 |
| sulD; dihydroneopterin aldolase / 2-amino-4-hydroxy-6-hydroxymethyldihydropteridine diphosphokinase [EC:4.1.2.25 2.7.6.3] | 1.490 | 2.89e-02 | 7.00e-01 |
| dgaD; D-glucosaminate PTS system EIID component | 1.457 | 2.90e-02 | 7.00e-01 |
| tetA; MFS transporter, DHA1 family, tetracycline resistance protein | 1.672 | 2.91e-02 | 7.00e-01 |
| panF; sodium/pantothenate symporter | -0.865 | 2.91e-02 | 7.00e-01 |
| rnmV; ribonuclease M5 [EC:3.1.26.8] | -0.677 | 2.93e-02 | 7.00e-01 |
| PIF1; ATP-dependent DNA helicase PIF1 [EC:3.6.4.12] | 0.529 | 2.95e-02 | 7.00e-01 |
| ABC.PE.S; peptide/nickel transport system substrate-binding protein | 0.556 | 2.95e-02 | 7.00e-01 |
| pepD; putative serine protease PepD [EC:3.4.21.-] | 1.539 | 2.97e-02 | 7.02e-01 |
| ogl; oligogalacturonide lyase [EC:4.2.2.6] | -1.613 | 3.00e-02 | 7.04e-01 |
| K06951; uncharacterized protein | -2.692 | 3.03e-02 | 7.04e-01 |
| NAMPT; nicotinamide phosphoribosyltransferase [EC:2.4.2.12] | -0.526 | 3.03e-02 | 7.04e-01 |
| gluQ; glutamyl-Q tRNA(Asp) synthetase [EC:6.1.1.-] | 0.994 | 3.07e-02 | 7.06e-01 |
| thiDE; hydroxymethylpyrimidine kinase / phosphomethylpyrimidine kinase / thiamine-phosphate diphosphorylase [EC:2.7.1.49 2.7.4.7 2.5.1.3] | 1.879 | 3.09e-02 | 7.07e-01 |
| CBS; cystathionine beta-synthase [EC:4.2.1.22] | 1.877 | 3.11e-02 | 7.07e-01 |
| cshB; ATP-dependent RNA helicase CshB [EC:3.6.4.13] | -0.636 | 3.18e-02 | 7.13e-01 |
| ABC.SP.S; putative spermidine/putrescine transport system substrate-binding protein | -0.991 | 3.27e-02 | 7.21e-01 |
| dck; deoxyadenosine/deoxycytidine kinase [EC:2.7.1.76 2.7.1.74] | 0.516 | 3.27e-02 | 7.21e-01 |
| mtsT; energy-coupling factor transport system substrate-specific component | -0.551 | 3.28e-02 | 7.21e-01 |
| sixA; phosphohistidine phosphatase [EC:3.1.3.-] | 2.732 | 3.28e-02 | 7.21e-01 |
| uctC; CoA:oxalate CoA-transferase [EC:2.8.3.19] | -0.701 | 3.32e-02 | 7.26e-01 |
| adhP; alcohol dehydrogenase, propanol-preferring [EC:1.1.1.1] | -0.629 | 3.40e-02 | 7.35e-01 |
| bioG; pimeloyl-[acyl-carrier protein] methyl ester esterase [EC:3.1.1.85] | -0.703 | 3.46e-02 | 7.39e-01 |
| hypF; hydrogenase maturation protein HypF | 0.523 | 3.46e-02 | 7.39e-01 |
| tmk, DTYMK; dTMP kinase [EC:2.7.4.9] | 0.553 | 3.51e-02 | 7.44e-01 |
| rarD; chloramphenicol-sensitive protein RarD | 1.865 | 3.54e-02 | 7.44e-01 |
| K09949; uncharacterized protein | 1.528 | 3.55e-02 | 7.44e-01 |
| K17202, eryG; erythritol transport system substrate-binding protein | -1.744 | 3.55e-02 | 7.44e-01 |
| eexD; ATP-binding cassette, subfamily C, bacterial EexD | -1.418 | 3.60e-02 | 7.49e-01 |
| ugpE; sn-glycerol 3-phosphate transport system permease protein | -0.890 | 3.67e-02 | 7.61e-01 |
| rutG; putative pyrimidine permease RutG | 0.678 | 3.69e-02 | 7.61e-01 |
| purR; purine operon repressor | -0.738 | 3.69e-02 | 7.61e-01 |
| lapB; ATP-binding cassette, subfamily C, bacterial LapB | -1.090 | 3.70e-02 | 7.61e-01 |
| comD; two-component system, LytTR family, sensor histidine kinase ComD [EC:2.7.13.3] | -1.752 | 3.83e-02 | 7.85e-01 |
| pbp2A; penicillin-binding protein 2A [EC:2.4.1.129 3.4.16.4] | -0.797 | 3.84e-02 | 7.85e-01 |
| ulaB, sgaB; ascorbate PTS system EIIB component [EC:2.7.1.194] | -0.893 | 3.89e-02 | 7.90e-01 |
| K11145; ribonuclease III family protein [EC:3.1.26.-] | -0.558 | 3.92e-02 | 7.92e-01 |
| E4.2.2.17; inulin fructotransferase (DFA-I-forming) [EC:4.2.2.17] | 1.259 | 3.99e-02 | 8.03e-01 |
| rph; ribonuclease PH [EC:2.7.7.56] | 0.798 | 4.00e-02 | 8.03e-01 |
| STE24; STE24 endopeptidase [EC:3.4.24.84] | 0.666 | 4.01e-02 | 8.04e-01 |
| ynjE; molybdopterin synthase sulfurtransferase [EC:2.8.1.11] | -1.862 | 4.07e-02 | 8.09e-01 |
| icmB, dotO; intracellular multiplication protein IcmB [EC:7.2.4.8] | -0.769 | 4.11e-02 | 8.09e-01 |
| dusC; tRNA-dihydrouridine synthase C [EC:1.-.-.-] | 1.813 | 4.12e-02 | 8.09e-01 |
| ygaC; uncharacterized protein | -0.673 | 4.12e-02 | 8.09e-01 |
| wcaL, amsK; colanic acid/amylovoran biosynthesis glycosyltransferase [EC:2.4.-.-] | -1.677 | 4.14e-02 | 8.09e-01 |
| gfrB; fructoselysine/glucoselysine PTS system EIIB component [EC:2.7.1.-] | 1.031 | 4.14e-02 | 8.09e-01 |
| VIT; vacuolar iron transporter family protein | -0.594 | 4.16e-02 | 8.09e-01 |
| tctB; putative tricarboxylic transport membrane protein | 1.014 | 4.20e-02 | 8.13e-01 |
| K07041; uncharacterized protein | -0.912 | 4.20e-02 | 8.13e-01 |
| dfx; superoxide reductase [EC:1.15.1.2] | -0.508 | 4.27e-02 | 8.22e-01 |
| pdaD; arginine decarboxylase [EC:4.1.1.19] | 1.161 | 4.27e-02 | 8.22e-01 |
| RP-L3e, RPL3; large subunit ribosomal protein L3e | 2.367 | 4.29e-02 | 8.23e-01 |
| glgE; starch synthase (maltosyl-transferring) [EC:2.4.99.16] | 1.294 | 4.37e-02 | 8.27e-01 |
| eptC; heptose-I-phosphate ethanolaminephosphotransferase [EC:2.7.8.-] | 1.443 | 4.44e-02 | 8.36e-01 |
| cusR, copR, silR; two-component system, OmpR family, copper resistance phosphate regulon response regulator CusR | -0.823 | 4.51e-02 | 8.39e-01 |
| rfaE2; D-glycero-beta-D-manno-heptose 1-phosphate adenylyltransferase [EC:2.7.7.70] | 0.615 | 4.51e-02 | 8.39e-01 |
| traD; conjugal transfer pilus assembly protein TraD | 1.279 | 4.53e-02 | 8.39e-01 |
| pilB; type IV pilus assembly protein PilB | 0.849 | 4.60e-02 | 8.50e-01 |
| waaC, rfaC; heptosyltransferase I [EC:2.4.-.-] | 1.616 | 4.69e-02 | 8.58e-01 |
| PEO1; twinkle protein [EC:3.6.4.12] | -0.767 | 4.75e-02 | 8.58e-01 |
| menH; 2-succinyl-6-hydroxy-2,4-cyclohexadiene-1-carboxylate synthase [EC:4.2.99.20] | 1.581 | 4.79e-02 | 8.58e-01 |
| rlmL, rlmK; 23S rRNA (guanine2445-N2)-methyltransferase / 23S rRNA (guanine2069-N7)-methyltransferase [EC:2.1.1.173 2.1.1.264] | 1.040 | 4.82e-02 | 8.59e-01 |
| E2.3.3.10; hydroxymethylglutaryl-CoA synthase [EC:2.3.3.10] | -0.805 | 4.99e-02 | 8.82e-01 |
| argAB; amino-acid N-acetyltransferase [EC:2.3.1.1] | 0.756 | 5.00e-02 | 8.82e-01 |
| Wt-Ex after vs. before | | | |
| DPEP; membrane dipeptidase [EC:3.4.13.19] | -1.089 | 1.86e-05 | 8.38e-02 |
| lmrB; MFS transporter, DHA2 family, lincomycin resistance protein | 0.945 | 4.90e-05 | 1.11e-01 |
| dnr; CRP/FNR family transcriptional regulator, dissimilatory nitrate respiration regulator | -2.854 | 1.07e-04 | 1.81e-01 |
| czcA, cusA, cnrA; heavy metal efflux system protein | -1.279 | 2.96e-04 | 3.00e-01 |
| aacC; aminoglycoside 3-N-acetyltransferase [EC:2.3.1.81] | -1.254 | 3.74e-04 | 3.18e-01 |
| cysI; sulfite reductase (NADPH) hemoprotein beta-component [EC:1.8.1.2] | 3.035 | 5.24e-04 | 3.95e-01 |
| cysJ; sulfite reductase (NADPH) flavoprotein alpha-component [EC:1.8.1.2] | 2.975 | 7.54e-04 | 4.27e-01 |
| TC.AAT; amino acid transporter, AAT family | 0.595 | 7.54e-04 | 4.27e-01 |
| ppc; phosphoenolpyruvate carboxylase [EC:4.1.1.31] | 1.311 | 8.28e-04 | 4.29e-01 |
| hslU; ATP-dependent HslUV protease ATP-binding subunit HslU | 0.739 | 1.07e-03 | 4.64e-01 |
| avtA; valine--pyruvate aminotransferase [EC:2.6.1.66] | 3.262 | 1.62e-03 | 5.99e-01 |
| K08884; serine/threonine protein kinase, bacterial [EC:2.7.11.1] | 1.394 | 1.81e-03 | 5.99e-01 |
| tadB; tight adherence protein B | 1.287 | 1.71e-03 | 5.99e-01 |
| E4.1.1.32, pckA, PCK; phosphoenolpyruvate carboxykinase (GTP) [EC:4.1.1.32] | 0.888 | 2.34e-03 | 6.86e-01 |
| hemE, UROD; uroporphyrinogen decarboxylase [EC:4.1.1.37] | -0.708 | 2.31e-03 | 6.86e-01 |
| tyrA; chorismate mutase / prephenate dehydrogenase [EC:5.4.99.5 1.3.1.12] | -0.729 | 2.43e-03 | 6.86e-01 |
| ata, sadA, emaA; trimeric autotransporter adhesin | -0.708 | 2.56e-03 | 6.94e-01 |
| aadK; aminoglycoside 6-adenylyltransferase [EC:2.7.7.-] | 1.307 | 3.04e-03 | 7.65e-01 |
| K09706; uncharacterized protein | 2.223 | 3.31e-03 | 7.74e-01 |
| bsdA; LysR family transcriptional regulator, salicylic acid-responsive activator of bsdBCD | 1.731 | 3.26e-03 | 7.74e-01 |
| thi4; sulfide-dependent adenosine diphosphate thiazole synthase [EC:2.4.2.59] | -1.344 | 4.05e-03 | 8.88e-01 |
| DNA2; DNA replication ATP-dependent helicase Dna2 [EC:3.6.4.12] | 1.999 | 4.29e-03 | 9.09e-01 |
| ganQ; arabinogalactan oligomer / maltooligosaccharide transport system permease protein | 0.751 | 4.52e-03 | 9.24e-01 |
| gspD; general secretion pathway protein D | 3.470 | 5.17e-03 | 9.75e-01 |
| bglB; beta-glucosidase [EC:3.2.1.21] | 0.669 | 5.14e-03 | 9.75e-01 |
| mmsA, iolA, ALDH6A1; malonate-semialdehyde dehydrogenase (acetylating) / methylmalonate-semialdehyde dehydrogenase [EC:1.2.1.18 1.2.1.27] | 0.870 | 5.37e-03 | 9.85e-01 |
| araM, egsA; glycerol-1-phosphate dehydrogenase [NAD(P)+] [EC:1.1.1.261] | 0.657 | 6.01e-03 | 9.95e-01 |
| amn; AMP nucleosidase [EC:3.2.2.4] | -0.504 | 5.82e-03 | 9.95e-01 |
| bccA, pccA; acetyl-CoA/propionyl-CoA carboxylase, biotin carboxylase, biotin carboxyl carrier protein [EC:6.4.1.2 6.4.1.3 6.3.4.14] | 2.266 | 5.84e-03 | 9.95e-01 |
| fabV, ter; enoyl-[acyl-carrier protein] reductase / trans-2-enoyl-CoA reductase (NAD+) [EC:1.3.1.9 1.3.1.44] | -1.172 | 4.85e-02 | 9.99e-01 |
| nuoC; NADH-quinone oxidoreductase subunit C [EC:7.1.1.2] | 2.582 | 2.62e-02 | 9.99e-01 |
| qor, CRYZ; NADPH:quinone reductase [EC:1.6.5.5] | 0.791 | 4.62e-02 | 9.99e-01 |
| E2.1.1.113; site-specific DNA-methyltransferase (cytosine-N4-specific) [EC:2.1.1.113] | -1.862 | 3.39e-02 | 9.99e-01 |
| fctD; glutamate formiminotransferase / 5-formyltetrahydrofolate cyclo-ligase [EC:2.1.2.5 6.3.3.2] | -0.651 | 1.25e-02 | 9.99e-01 |
| dapD; 2,3,4,5-tetrahydropyridine-2,6-dicarboxylate N-succinyltransferase [EC:2.3.1.117] | 0.568 | 4.67e-02 | 9.99e-01 |
| POMT, pmt; dolichyl-phosphate-mannose-protein mannosyltransferase [EC:2.4.1.109] | 0.991 | 1.64e-02 | 9.99e-01 |
| FDFT1; farnesyl-diphosphate farnesyltransferase [EC:2.5.1.21] | 3.025 | 9.55e-03 | 9.99e-01 |
| fucK; L-fuculokinase [EC:2.7.1.51] | 2.803 | 9.52e-03 | 9.99e-01 |
| pdtaS; two-component system, sensor histidine kinase PdtaS [EC:2.7.13.3] | 1.709 | 2.16e-02 | 9.99e-01 |
| UGP2, galU, galF; UTP--glucose-1-phosphate uridylyltransferase [EC:2.7.7.9] | 0.605 | 2.71e-02 | 9.99e-01 |
| yqgT; g-D-glutamyl-meso-diaminopimelate peptidase [EC:3.4.19.11] | 1.252 | 2.64e-02 | 9.99e-01 |
| ptrB; oligopeptidase B [EC:3.4.21.83] | 1.999 | 6.60e-03 | 9.99e-01 |
| hslV, clpQ; ATP-dependent HslUV protease, peptidase subunit HslV [EC:3.4.25.2] | 0.758 | 1.63e-02 | 9.99e-01 |
| atzF; allophanate hydrolase [EC:3.5.1.54] | -1.573 | 3.12e-02 | 9.99e-01 |
| speB; agmatinase [EC:3.5.3.11] | 0.503 | 1.16e-02 | 9.99e-01 |
| add, ADA; adenosine deaminase [EC:3.5.4.4] | 0.617 | 3.39e-02 | 9.99e-01 |
| kdpC; potassium-transporting ATPase KdpC subunit | -1.002 | 2.99e-02 | 9.99e-01 |
| arsA, ASNA1, GET3; arsenite/tail-anchored protein-transporting ATPase [EC:7.3.2.7 7.3.-.-] | -1.016 | 4.24e-02 | 9.99e-01 |
| metB; cystathionine gamma-synthase [EC:2.5.1.48] | 1.177 | 1.86e-02 | 9.99e-01 |
| metC; cysteine-S-conjugate beta-lyase [EC:4.4.1.13] | 0.524 | 3.47e-02 | 9.99e-01 |
| E5.1.3.15; glucose-6-phosphate 1-epimerase [EC:5.1.3.15] | 1.942 | 4.15e-02 | 9.99e-01 |
| ABC.ZM.A; zinc/manganese transport system ATP-binding protein | 1.006 | 7.41e-03 | 9.99e-01 |
| ABC.ZM.S; zinc/manganese transport system substrate-binding protein | 1.041 | 1.80e-02 | 9.99e-01 |
| comGC; competence protein ComGC | -1.744 | 4.41e-02 | 9.99e-01 |
| crtB; 15-cis-phytoene synthase [EC:2.5.1.32] | 2.176 | 4.97e-02 | 9.99e-01 |
| lacR; DeoR family transcriptional regulator, lactose phosphotransferase system repressor | -2.189 | 3.81e-02 | 9.99e-01 |
| nagC; N-acetylglucosamine repressor | 0.994 | 3.72e-02 | 9.99e-01 |
| pilB; type IV pilus assembly protein PilB | 0.887 | 1.24e-02 | 9.99e-01 |
| celC, chbA; cellobiose PTS system EIIA component [EC:2.7.1.196 2.7.1.205] | -1.066 | 1.22e-02 | 9.99e-01 |
| celB, chbC; cellobiose PTS system EIIC component | -0.655 | 4.13e-02 | 9.99e-01 |
| RP-S2e, RPS2; small subunit ribosomal protein S2e | -1.964 | 3.44e-02 | 9.99e-01 |
| thiF; sulfur carrier protein ThiS adenylyltransferase [EC:2.7.7.73] | 1.071 | 2.91e-02 | 9.99e-01 |
| ACR3, arsB; arsenite transporter | -0.771 | 2.53e-02 | 9.99e-01 |
| fruR2, fruR; DeoR family transcriptional regulator, fructose operon transcriptional repressor | -0.829 | 2.99e-02 | 9.99e-01 |
| TC.NCS2; nucleobase:cation symporter-2, NCS2 family | 0.637 | 4.73e-02 | 9.99e-01 |
| licR; lichenan operon transcriptional antiterminator | -1.618 | 7.66e-03 | 9.99e-01 |
| hrpA; ATP-dependent helicase HrpA [EC:3.6.4.13] | 0.742 | 1.23e-02 | 9.99e-01 |
| hrpB; ATP-dependent helicase HrpB [EC:3.6.4.13] | 2.035 | 1.13e-02 | 9.99e-01 |
| sspB; stringent starvation protein B | 2.115 | 3.07e-02 | 9.99e-01 |
| rnd; ribonuclease D [EC:3.1.13.5] | 1.526 | 1.97e-02 | 9.99e-01 |
| splB; spore photoproduct lyase [EC:4.1.99.14] | 1.010 | 1.27e-02 | 9.99e-01 |
| helY; ATP-dependent RNA helicase HelY [EC:3.6.4.-] | 1.834 | 2.85e-02 | 9.99e-01 |
| eutB; ethanolamine ammonia-lyase large subunit [EC:4.3.1.7] | -0.971 | 3.56e-02 | 9.99e-01 |
| murJ, mviN; putative peptidoglycan lipid II flippase | 0.657 | 1.53e-02 | 9.99e-01 |
| E1.1.1.65; pyridoxine 4-dehydrogenase [EC:1.1.1.65] | 1.828 | 4.64e-02 | 9.99e-01 |
| cytR; LacI family transcriptional regulator, repressor for deo operon, udp, cdd, tsx, nupC, and nupG | 0.527 | 3.28e-02 | 9.99e-01 |
| dusC; tRNA-dihydrouridine synthase C [EC:1.-.-.-] | 2.211 | 3.49e-02 | 9.99e-01 |
| pdhR; GntR family transcriptional regulator, transcriptional repressor for pyruvate dehydrogenase complex | -0.505 | 1.71e-02 | 9.99e-01 |
| mscK, kefA, aefA; potassium-dependent mechanosensitive channel | 0.706 | 4.69e-02 | 9.99e-01 |
| aas; acyl-[acyl-carrier-protein]-phospholipid O-acyltransferase / long-chain-fatty-acid--[acyl-carrier-protein] ligase [EC:2.3.1.40 6.2.1.20] | 2.194 | 4.16e-02 | 9.99e-01 |
| shc; squalene-hopene/tetraprenyl-beta-curcumene cyclase [EC:5.4.99.17 4.2.1.129] | 2.344 | 9.77e-03 | 9.99e-01 |
| ccdA; cytochrome c-type biogenesis protein | -0.610 | 1.47e-02 | 9.99e-01 |
| pgi1; glucose-6-phosphate isomerase, archaeal [EC:5.3.1.9] | -1.254 | 1.94e-02 | 9.99e-01 |
| K07177; Lon-like protease | 1.080 | 9.84e-03 | 9.99e-01 |
| ALG14; beta-1,4-N-acetylglucosaminyltransferase [EC:2.4.1.141] | -0.972 | 8.41e-03 | 9.99e-01 |
| rstA1; phage replication initiation protein | 0.843 | 9.38e-03 | 9.99e-01 |
| nreB; two-component system, NarL family, sensor histidine kinase NreB [EC:2.7.13.3] | 0.759 | 4.74e-02 | 9.99e-01 |
| ycbA, glnK; two-component system, sensor histidine kinase YcbA [EC:2.7.13.3] | -1.216 | 3.29e-02 | 9.99e-01 |
| ycbB, glnL; two-component system, response regulator YcbB | 2.568 | 4.73e-02 | 9.99e-01 |
| yqhD; NADP-dependent alcohol dehydrogenase [EC:1.1.-.-] | 0.609 | 4.80e-02 | 9.99e-01 |
| pepD; putative serine protease PepD [EC:3.4.21.-] | 0.968 | 1.45e-02 | 9.99e-01 |
| mtnE, mtnV; L-glutamine---4-(methylsulfanyl)-2-oxobutanoate aminotransferase [EC:2.6.1.117] | 1.141 | 1.93e-02 | 9.99e-01 |
| K08972; putative membrane protein | 1.709 | 2.08e-02 | 9.99e-01 |
| rutG; putative pyrimidine permease RutG | 0.855 | 3.02e-02 | 9.99e-01 |
| K09155; uncharacterized protein | -1.404 | 3.58e-02 | 9.99e-01 |
| K09163; uncharacterized protein | 1.062 | 2.76e-02 | 9.99e-01 |
| tagF; CDP-glycerol glycerophosphotransferase [EC:2.7.8.12] | -0.825 | 1.83e-02 | 9.99e-01 |
| ABC.MN.A; manganese/iron transport system ATP-binding protein | 2.745 | 2.56e-02 | 9.99e-01 |
| K09954; uncharacterized protein | 2.641 | 3.60e-02 | 9.99e-01 |
| K10121, msmF; fructooligosaccharide transport system permease protein | -1.142 | 2.54e-02 | 9.99e-01 |
| ABC-2.CYL.A, cylA; multidrug/hemolysin transport system ATP-binding protein | 1.061 | 1.07e-02 | 9.99e-01 |
| tqsA; AI-2 transport protein TqsA | 2.395 | 2.07e-02 | 9.99e-01 |
| yejA; microcin C transport system substrate-binding protein | 2.719 | 2.53e-02 | 9.99e-01 |
| yejE; microcin C transport system permease protein | 3.056 | 9.05e-03 | 9.99e-01 |
| thiDE; hydroxymethylpyrimidine kinase / phosphomethylpyrimidine kinase / thiamine-phosphate diphosphorylase [EC:2.7.1.49 2.7.4.7 2.5.1.3] | 1.155 | 2.06e-02 | 9.99e-01 |
| SLC10A7, P7; solute carrier family 10 (sodium/bile acid cotransporter), member 7 | 3.031 | 9.38e-03 | 9.99e-01 |
| SMARCAL1, HARP; SWI/SNF-related matrix-associated actin-dependent regulator of chromatin subfamily A-like protein 1 [EC:3.6.4.12] | 0.591 | 2.34e-02 | 9.99e-01 |
| ssuB; sulfonate transport system ATP-binding protein [EC:3.6.3.-] | 0.761 | 1.83e-02 | 9.99e-01 |
| nikC, cntC; nickel transport system permease protein | -1.528 | 1.14e-02 | 9.99e-01 |
| srfAA, lchAA; surfactin family lipopeptide synthetase A | -0.968 | 3.08e-02 | 9.99e-01 |
| glgM; alpha-maltose-1-phosphate synthase [EC:2.4.1.342] | 1.906 | 2.39e-02 | 9.99e-01 |
| K16149; 1,4-alpha-glucan branching enzyme [EC:2.4.1.18] | 0.768 | 3.93e-02 | 9.99e-01 |
| lacS, galP, rafP; lactose/raffinose/galactose permease | 0.524 | 3.08e-02 | 9.99e-01 |
| K17076, lysY; putative lysine transport system ATP-binding protein [EC:3.6.3.-] | -0.915 | 2.17e-02 | 9.99e-01 |
| chiF; putative chitobiose transport system permease protein | 1.398 | 3.53e-02 | 9.99e-01 |
| cwlK; peptidoglycan LD-endopeptidase CwlK [EC:3.4.-.-] | 1.189 | 3.77e-02 | 9.99e-01 |
| E2.7.8.39; archaetidylinositol phosphate synthase [EC:2.7.8.39] | -0.850 | 2.11e-02 | 9.99e-01 |
| wbiB; dTDP-L-rhamnose 4-epimerase [EC:5.1.3.25] | -1.135 | 7.57e-03 | 9.99e-01 |
| bmaC; fibronectin-binding autotransporter adhesin | 2.103 | 2.01e-02 | 9.99e-01 |
| tabA; biofilm protein TabA | -0.888 | 4.51e-02 | 9.99e-01 |
| mcsB; protein arginine kinase [EC:2.7.14.1] | 1.439 | 4.73e-02 | 9.99e-01 |
| ywlE; protein arginine phosphatase [EC:3.9.1.2] | 1.890 | 4.42e-02 | 9.99e-01 |
| bcsC; cellulose synthase operon protein C | 3.876 | 1.47e-02 | 9.99e-01 |
| fdtB; dTDP-3-amino-3,6-dideoxy-alpha-D-galactopyranose transaminase [EC:2.6.1.90] | -1.667 | 1.50e-02 | 9.99e-01 |
| rpfB; resuscitation-promoting factor RpfB | 1.901 | 8.91e-03 | 9.99e-01 |
| ybiO; moderate conductance mechanosensitive channel | 0.682 | 2.71e-02 | 9.99e-01 |
| mnxG; manganese oxidase [EC:1.16.3.3] | 2.365 | 1.88e-02 | 9.99e-01 |
| MACROD, ymdB; O-acetyl-ADP-ribose deacetylase [EC:3.1.1.106] | -0.526 | 1.37e-02 | 9.99e-01 |
| acdAB; acetate---CoA ligase (ADP-forming) [EC:6.2.1.13] | 0.512 | 4.49e-02 | 9.99e-01 |
| ecfTA; energy-coupling factor transport system permease/ATP-binding protein [EC:3.6.3.-] | 1.858 | 4.43e-02 | 9.99e-01 |
| PGC-1α-Ex before vs. Wt-Ex before | | | |
| cbiM; cobalt/nickel transport system permease protein | -0.734 | 1.30e-08 | 6.79e-05\* |
| aadK; aminoglycoside 6-adenylyltransferase [EC:2.7.7.-] | 2.163 | 2.03e-08 | 6.79e-05\* |
| araM, egsA; glycerol-1-phosphate dehydrogenase [NAD(P)+] [EC:1.1.1.261] | 1.122 | 5.49e-08 | 1.22e-04\* |
| bacA; vitamin B12/bleomycin/antimicrobial peptide transport system ATP-binding/permease protein | -1.731 | 1.76e-07 | 2.94e-04\* |
| oprO\_P; phosphate-selective porin OprO and OprP | -3.509 | 3.02e-07 | 4.04e-04\* |
| glnD; [protein-PII] uridylyltransferase [EC:2.7.7.59] | -2.293 | 4.07e-07 | 4.54e-04\* |
| E1.2.1.68; coniferyl-aldehyde dehydrogenase [EC:1.2.1.68] | -4.043 | 6.33e-07 | 6.05e-04\* |
| traV; conjugal transfer pilus assembly protein TraV | -4.331 | 1.09e-06 | 9.11e-04\* |
| dmsD; putative dimethyl sulfoxide reductase chaperone | -1.429 | 1.34e-06 | 9.56e-04\* |
| ccmF; cytochrome c-type biogenesis protein CcmF | -1.034 | 1.43e-06 | 9.56e-04\* |
| dptH; DNA phosphorothioation-dependent restriction protein DptH | 0.823 | 2.03e-06 | 1.23e-03\* |
| aac6-I, aacA7; aminoglycoside 6'-N-acetyltransferase I [EC:2.3.1.82] | 2.280 | 2.80e-06 | 1.56e-03\* |
| apaH; bis(5'-nucleosyl)-tetraphosphatase (symmetrical) [EC:3.6.1.41] | -2.655 | 3.26e-06 | 1.68e-03\* |
| hrpA; ATP-dependent helicase HrpA [EC:3.6.4.13] | -2.616 | 4.17e-06 | 1.99e-03\* |
| paaK; phenylacetate-CoA ligase [EC:6.2.1.30] | 0.811 | 4.56e-06 | 2.03e-03\* |
| ubiG; 2-polyprenyl-6-hydroxyphenyl methylase / 3-demethylubiquinone-9 3-methyltransferase [EC:2.1.1.222 2.1.1.64] | 1.144 | 6.97e-06 | 2.91e-03\* |
| agrC, blpH, fsrC; two-component system, LytTR family, sensor histidine kinase AgrC [EC:2.7.13.3] | 1.060 | 7.44e-06 | 2.93e-03\* |
| recC; exodeoxyribonuclease V gamma subunit [EC:3.1.11.5] | -2.168 | 8.91e-06 | 3.31e-03\* |
| sucC; succinyl-CoA synthetase beta subunit [EC:6.2.1.5] | -2.259 | 1.16e-05 | 4.08e-03\* |
| ssp; subtilase-type serine protease [EC:3.4.21.-] | -2.696 | 1.37e-05 | 4.58e-03\* |
| dapE; succinyl-diaminopimelate desuccinylase [EC:3.5.1.18] | -0.993 | 1.93e-05 | 6.15e-03\* |
| manY; mannose PTS system EIIC component | -0.722 | 2.09e-05 | 6.35e-03\* |
| moeA; molybdopterin molybdotransferase [EC:2.10.1.1] | -1.221 | 2.37e-05 | 6.37e-03\* |
| melB; melibiose permease | 1.299 | 2.37e-05 | 6.37e-03\* |
| fdhB; formate dehydrogenase (NADP+) beta subunit [EC:1.17.1.10] | -1.297 | 2.38e-05 | 6.37e-03\* |
| MAN; mannan endo-1,4-beta-mannosidase [EC:3.2.1.78] | 0.933 | 2.55e-05 | 6.47e-03\* |
| lolB; outer membrane lipoprotein LolB | -3.054 | 2.61e-05 | 6.47e-03\* |
| abgT; aminobenzoyl-glutamate transport protein | 1.121 | 2.89e-05 | 6.71e-03\* |
| OGDH, sucA; 2-oxoglutarate dehydrogenase E1 component [EC:1.2.4.2] | -2.048 | 2.91e-05 | 6.71e-03\* |
| TC.BAT1; bacterial/archaeal transporter family protein | 0.570 | 3.12e-05 | 6.73e-03\* |
| wcaE; putative colanic acid biosynthesis glycosyltransferase [EC:2.4.-.-] | 1.534 | 3.39e-05 | 6.76e-03\* |
| yahK; alcohol dehydrogenase (NADP+) [EC:1.1.1.2] | -1.972 | 3.39e-05 | 6.76e-03\* |
| blpB; membrane fusion protein, peptide pheromone/bacteriocin exporter | 0.945 | 3.65e-05 | 6.76e-03\* |
| glnE; [glutamine synthetase] adenylyltransferase / [glutamine synthetase]-adenylyl-L-tyrosine phosphorylase [EC:2.7.7.42 2.7.7.89] | -1.424 | 3.67e-05 | 6.76e-03\* |
| K09973; uncharacterized protein | 0.946 | 3.83e-05 | 6.76e-03\* |
| anmK; anhydro-N-acetylmuramic acid kinase [EC:2.7.1.170] | 0.984 | 4.33e-05 | 7.14e-03\* |
| inuJ; inulosucrase [EC:2.4.1.9] | -1.875 | 4.38e-05 | 7.14e-03\* |
| ptsN; nitrogen PTS system EIIA component [EC:2.7.1.-] | -4.265 | 5.13e-05 | 7.89e-03\* |
| E3.2.1.14; chitinase [EC:3.2.1.14] | 1.010 | 5.17e-05 | 7.89e-03\* |
| secB; preprotein translocase subunit SecB | -2.652 | 5.19e-05 | 7.89e-03\* |
| fepA, pfeA, iroN, pirA; ferric enterobactin receptor | 0.928 | 5.66e-05 | 8.37e-03\* |
| yciA; acyl-CoA thioesterase YciA [EC:3.1.2.-] | -4.144 | 5.76e-05 | 8.37e-03\* |
| narX; two-component system, NarL family, nitrate/nitrite sensor histidine kinase NarX [EC:2.7.13.3] | -3.170 | 6.63e-05 | 9.43e-03\* |
| mepR; MarR family transcriptional regulator, repressor for mepA | 1.332 | 7.09e-05 | 9.45e-03\* |
| ssuC; sulfonate transport system permease protein | -1.384 | 7.14e-05 | 9.45e-03\* |
| eptC; heptose-I-phosphate ethanolaminephosphotransferase [EC:2.7.8.-] | -2.143 | 7.17e-05 | 9.45e-03\* |
| lptD, imp, ostA; LPS-assembly protein | -2.302 | 7.21e-05 | 9.45e-03\* |
| agrD; AgrD protein | 2.895 | 7.95e-05 | 9.89e-03\* |
| rnb; exoribonuclease II [EC:3.1.13.1] | -1.408 | 7.97e-05 | 9.89e-03\* |
| K20975; two-component system, sensor histidine kinase [EC:2.7.13.3] | -2.362 | 8.08e-05 | 9.89e-03\* |
| lplT; MFS transporter, LPLT family, lysophospholipid transporter | -2.256 | 8.13e-05 | 9.89e-03\* |
| mapA; maltose phosphorylase [EC:2.4.1.8] | 0.801 | 8.43e-05 | 1.01e-02\* |
| E1.12.7.2G; ferredoxin hydrogenase gamma subunit [EC:1.12.7.2] | -2.039 | 8.61e-05 | 1.01e-02\* |
| cbiN; cobalt/nickel transport protein | -1.186 | 9.23e-05 | 1.06e-02\* |
| radD; DNA repair protein RadD | 1.040 | 1.01e-04 | 1.12e-02\* |
| ttrS; two-component system, LuxR family, sensor histidine kinase TtrS [EC:2.7.13.3] | -2.154 | 1.07e-04 | 1.16e-02\* |
| cah; cephalosporin-C deacetylase [EC:3.1.1.41] | 1.044 | 1.13e-04 | 1.20e-02\* |
| pgpA; phosphatidylglycerophosphatase A [EC:3.1.3.27] | 0.747 | 1.15e-04 | 1.21e-02\* |
| cssS; two-component system, OmpR family, sensor histidine kinase CssS [EC:2.7.13.3] | -3.757 | 1.21e-04 | 1.21e-02\* |
| E3.2.1.85, lacG; 6-phospho-beta-galactosidase [EC:3.2.1.85] | -3.250 | 1.22e-04 | 1.21e-02\* |
| oprN; outer membrane protein, multidrug efflux system | -2.393 | 1.23e-04 | 1.21e-02\* |
| SLC9B1\_2; solute carrier family 9B (sodium/hydrogen exchanger), member 1/2 | -2.520 | 1.27e-04 | 1.21e-02\* |
| zapA; cell division protein ZapA | 0.861 | 1.32e-04 | 1.22e-02\* |
| tolA; colicin import membrane protein | -0.885 | 1.38e-04 | 1.26e-02\* |
| resE; two-component system, OmpR family, sensor histidine kinase ResE [EC:2.7.13.3] | -2.021 | 1.49e-04 | 1.35e-02\* |
| mdcD; malonate decarboxylase beta subunit [EC:4.1.1.87] | -2.684 | 1.56e-04 | 1.35e-02\* |
| lapB; lipopolysaccharide assembly protein B | -2.100 | 1.56e-04 | 1.35e-02\* |
| phsA, psrA; thiosulfate reductase / polysulfide reductase chain A [EC:1.8.5.5] | -0.975 | 1.57e-04 | 1.35e-02\* |
| nfnB, nfsB; nitroreductase / dihydropteridine reductase [EC:1.-.-.- 1.5.1.34] | -3.699 | 1.62e-04 | 1.37e-02\* |
| wzxC; lipopolysaccharide exporter | 1.155 | 1.70e-04 | 1.42e-02\* |
| dmsA; anaerobic dimethyl sulfoxide reductase subunit A [EC:1.8.5.3] | -0.689 | 1.81e-04 | 1.50e-02\* |
| nrfC; protein NrfC | -1.265 | 1.89e-04 | 1.52e-02\* |
| AUR1; inositol phosphorylceramide synthase catalytic subunit [EC:2.7.1.227] | 0.826 | 1.91e-04 | 1.52e-02\* |
| ybjI; FMN hydrolase / 5-amino-6-(5-phospho-D-ribitylamino)uracil phosphatase [EC:3.1.3.102 3.1.3.104] | -1.472 | 1.92e-04 | 1.52e-02\* |
| K08884; serine/threonine protein kinase, bacterial [EC:2.7.11.1] | 1.776 | 1.93e-04 | 1.52e-02\* |
| TC.DCUC, dcuC, dcuD; C4-dicarboxylate transporter, DcuC family | -0.999 | 1.99e-04 | 1.55e-02\* |
| narL; two-component system, NarL family, nitrate/nitrite response regulator NarL | -2.411 | 2.06e-04 | 1.58e-02\* |
| cbiO; cobalt/nickel transport system ATP-binding protein | -0.739 | 2.08e-04 | 1.58e-02\* |
| napG; ferredoxin-type protein NapG | -1.039 | 2.24e-04 | 1.68e-02\* |
| agaF; N-acetylgalactosamine PTS system EIIA component [EC:2.7.1.-] | -2.447 | 2.26e-04 | 1.68e-02\* |
| tesA; acyl-CoA thioesterase I [EC:3.1.2.- 3.1.2.2 3.1.1.2 3.1.1.5] | -2.555 | 2.35e-04 | 1.73e-02\* |
| buk; butyrate kinase [EC:2.7.2.7] | 0.567 | 2.39e-04 | 1.74e-02\* |
| dmsB; dimethyl sulfoxide reductase iron-sulfur subunit | -1.312 | 2.45e-04 | 1.76e-02\* |
| K07018; uncharacterized protein | -2.274 | 2.54e-04 | 1.79e-02\* |
| pseF; pseudaminic acid cytidylyltransferase [EC:2.7.7.81] | 1.048 | 2.54e-04 | 1.79e-02\* |
| K07217; Mn-containing catalase | 0.592 | 2.66e-04 | 1.85e-02\* |
| gluQ; glutamyl-Q tRNA(Asp) synthetase [EC:6.1.1.-] | -2.093 | 2.69e-04 | 1.85e-02\* |
| ccdA; cytochrome c-type biogenesis protein | -1.203 | 2.80e-04 | 1.91e-02\* |
| dld; D-lactate dehydrogenase | 0.799 | 3.02e-04 | 2.04e-02\* |
| ygjK; putative isomerase | 0.774 | 3.14e-04 | 2.10e-02\* |
| aidA-I, misL; autotransporter family porin | -1.165 | 3.31e-04 | 2.19e-02\* |
| mtrA; AraC family transcriptional regulator, activator of mtrCDE | 2.395 | 3.39e-04 | 2.22e-02\* |
| ecnB; entericidin B | -4.176 | 3.47e-04 | 2.25e-02\* |
| yhgE; putative membrane protein | -1.206 | 3.51e-04 | 2.26e-02\* |
| K08961; chondroitin-sulfate-ABC endolyase/exolyase [EC:4.2.2.20 4.2.2.21] | 1.254 | 3.54e-04 | 2.26e-02\* |
| TMEM165, GDT1; Ca2+/H+ antiporter, TMEM165/GDT1 family | -3.171 | 3.58e-04 | 2.26e-02\* |
| emrB; MFS transporter, DHA2 family, multidrug resistance protein | 1.026 | 3.64e-04 | 2.28e-02\* |
| K07484; transposase | 0.509 | 3.75e-04 | 2.32e-02\* |
| E4.1.1.15, gadB, gadA, GAD; glutamate decarboxylase [EC:4.1.1.15] | -0.885 | 3.80e-04 | 2.33e-02\* |
| slyB; outer membrane lipoprotein SlyB | -2.064 | 3.85e-04 | 2.34e-02\* |
| yqgT; g-D-glutamyl-meso-diaminopimelate peptidase [EC:3.4.19.11] | 1.773 | 4.04e-04 | 2.42e-02\* |
| ybaZ; methylated-DNA-protein-cysteine methyltransferase related protein | 0.933 | 4.06e-04 | 2.42e-02\* |
| ABC.X2.A; putative ABC transport system ATP-binding protein | 1.237 | 4.10e-04 | 2.42e-02\* |
| K07078; uncharacterized protein | 0.747 | 4.15e-04 | 2.43e-02\* |
| mdtC; multidrug efflux pump | 1.494 | 4.24e-04 | 2.46e-02\* |
| K07121; uncharacterized protein | -1.668 | 4.34e-04 | 2.50e-02\* |
| traD; conjugal transfer pilus assembly protein TraD | -1.922 | 4.40e-04 | 2.50e-02\* |
| dgkA, DGK; diacylglycerol kinase (ATP) [EC:2.7.1.107] | 1.132 | 4.43e-04 | 2.50e-02\* |
| E3.2.1.4; endoglucanase [EC:3.2.1.4] | 0.746 | 4.50e-04 | 2.50e-02\* |
| aacC; aminoglycoside 3-N-acetyltransferase [EC:2.3.1.81] | -1.211 | 4.51e-04 | 2.50e-02\* |
| urdA; urocanate reductase [EC:1.3.99.33] | -0.690 | 4.52e-04 | 2.50e-02\* |
| rlmE, rrmJ, ftsJ; 23S rRNA (uridine2552-2'-O)-methyltransferase [EC:2.1.1.166] | -1.174 | 4.58e-04 | 2.50e-02\* |
| K09924; uncharacterized protein | 1.291 | 4.59e-04 | 2.50e-02\* |
| acpS; holo-[acyl-carrier protein] synthase [EC:2.7.8.7] | -2.545 | 4.70e-04 | 2.54e-02\* |
| cdhC; acetyl-CoA decarbonylase/synthase, CODH/ACS complex subunit beta [EC:2.3.1.169] | -2.962 | 4.80e-04 | 2.57e-02\* |
| nadR; HTH-type transcriptional regulator, transcriptional repressor of NAD biosynthesis genes [EC:2.7.7.1 2.7.1.22] | 3.163 | 4.92e-04 | 2.59e-02\* |
| PGD, gnd, gntZ; 6-phosphogluconate dehydrogenase [EC:1.1.1.44 1.1.1.343] | 0.850 | 5.14e-04 | 2.67e-02\* |
| NIT1, ybeM; deaminated glutathione amidase [EC:3.5.1.128] | -2.826 | 5.32e-04 | 2.74e-02\* |
| yaaU; MFS transporter, putative metabolite transport protein | -0.824 | 5.59e-04 | 2.84e-02\* |
| cysD; sulfate adenylyltransferase subunit 2 [EC:2.7.7.4] | 0.526 | 5.61e-04 | 2.84e-02\* |
| dsbC; thiol:disulfide interchange protein DsbC [EC:5.3.4.1] | -1.863 | 5.70e-04 | 2.87e-02\* |
| ebgA; evolved beta-galactosidase subunit alpha [EC:3.2.1.23] | 1.934 | 5.74e-04 | 2.87e-02\* |
| clpA; ATP-dependent Clp protease ATP-binding subunit ClpA | 0.705 | 5.99e-04 | 2.95e-02\* |
| potC; spermidine/putrescine transport system permease protein | 0.607 | 6.05e-04 | 2.96e-02\* |
| K00666; fatty-acyl-CoA synthase [EC:6.2.1.-] | 0.963 | 6.13e-04 | 2.97e-02\* |
| torZ; trimethylamine-N-oxide reductase (cytochrome c) [EC:1.7.2.3] | -0.828 | 6.33e-04 | 3.04e-02\* |
| waaC, rfaC; heptosyltransferase I [EC:2.4.-.-] | -1.906 | 6.43e-04 | 3.07e-02\* |
| MOCS2B, moaE; molybdopterin synthase catalytic subunit [EC:2.8.1.12] | -1.643 | 6.61e-04 | 3.14e-02\* |
| ssuA; sulfonate transport system substrate-binding protein | -1.042 | 6.78e-04 | 3.18e-02\* |
| fctD; glutamate formiminotransferase / 5-formyltetrahydrofolate cyclo-ligase [EC:2.1.2.5 6.3.3.2] | -1.078 | 6.79e-04 | 3.18e-02\* |
| DHODH, pyrD; dihydroorotate dehydrogenase [EC:1.3.5.2] | -1.396 | 6.93e-04 | 3.20e-02\* |
| hr; hemerythrin | 1.125 | 7.14e-04 | 3.27e-02\* |
| lacE; lactose PTS system EIICB component [EC:2.7.1.207] | -2.846 | 7.18e-04 | 3.27e-02\* |
| potB; spermidine/putrescine transport system permease protein | 0.635 | 7.46e-04 | 3.37e-02\* |
| terD; tellurium resistance protein TerD | 0.511 | 7.60e-04 | 3.40e-02\* |
| zapD; cell division protein ZapD | -2.173 | 7.77e-04 | 3.40e-02\* |
| NAMPT; nicotinamide phosphoribosyltransferase [EC:2.4.2.12] | 1.481 | 7.89e-04 | 3.43e-02\* |
| cysN; sulfate adenylyltransferase subunit 1 [EC:2.7.7.4] | 0.650 | 7.97e-04 | 3.44e-02\* |
| HGSNAT; heparan-alpha-glucosaminide N-acetyltransferase [EC:2.3.1.78] | 0.923 | 8.11e-04 | 3.46e-02\* |
| higB; mRNA interferase HigB [EC:3.1.-.-] | 1.032 | 8.15e-04 | 3.46e-02\* |
| mdoB; phosphoglycerol transferase [EC:2.7.8.20] | -3.163 | 8.23e-04 | 3.46e-02\* |
| agaR; DeoR family transcriptional regulator, aga operon transcriptional repressor | -0.920 | 8.23e-04 | 3.46e-02\* |
| blpA, lagD; ATP-binding cassette, subfamily C, bacteriocin exporter | 0.856 | 8.31e-04 | 3.47e-02\* |
| prpC, phpP; PPM family protein phosphatase [EC:3.1.3.16] | -0.883 | 8.62e-04 | 3.55e-02\* |
| argAB; amino-acid N-acetyltransferase [EC:2.3.1.1] | -1.927 | 8.65e-04 | 3.55e-02\* |
| mdcB; triphosphoribosyl-dephospho-CoA synthase [EC:2.4.2.52] | -2.062 | 8.75e-04 | 3.57e-02\* |
| speG, SAT; diamine N-acetyltransferase [EC:2.3.1.57] | 0.540 | 8.94e-04 | 3.62e-02\* |
| pyrD; dihydroorotate dehydrogenase (fumarate) [EC:1.3.98.1] | 1.029 | 9.17e-04 | 3.69e-02\* |
| ompU; outer membrane protein OmpU | -2.598 | 9.48e-04 | 3.80e-02\* |
| fruR2, fruR; DeoR family transcriptional regulator, fructose operon transcriptional repressor | -1.988 | 9.58e-04 | 3.80e-02\* |
| potD; spermidine/putrescine transport system substrate-binding protein | 0.626 | 9.63e-04 | 3.80e-02\* |
| STE24; STE24 endopeptidase [EC:3.4.24.84] | -0.820 | 9.70e-04 | 3.80e-02\* |
| aqpZ; aquaporin Z | -0.684 | 9.71e-04 | 3.80e-02\* |
| mtnE, mtnV; L-glutamine---4-(methylsulfanyl)-2-oxobutanoate aminotransferase [EC:2.6.1.117] | -2.794 | 9.81e-04 | 3.81e-02\* |
| pspA; phage shock protein A | -1.500 | 9.99e-04 | 3.86e-02\* |
| qseC; two-component system, OmpR family, sensor histidine kinase QseC [EC:2.7.13.3] | 0.947 | 1.01e-03 | 3.87e-02\* |
| K07041; uncharacterized protein | 1.440 | 1.05e-03 | 3.97e-02\* |
| rclC; reactive chlorine resistance protein C | 1.032 | 1.05e-03 | 3.97e-02\* |
| hyaA, hybO; hydrogenase small subunit [EC:1.12.99.6] | -1.128 | 1.08e-03 | 4.07e-02\* |
| chrR, NQR; chromate reductase, NAD(P)H dehydrogenase (quinone) | -2.327 | 1.10e-03 | 4.08e-02\* |
| ugtP; processive 1,2-diacylglycerol beta-glucosyltransferase [EC:2.4.1.315] | -1.985 | 1.14e-03 | 4.20e-02\* |
| nrfD; protein NrfD | -0.865 | 1.15e-03 | 4.24e-02\* |
| virB1; type IV secretion system protein VirB1 | -2.551 | 1.19e-03 | 4.36e-02\* |
| K02477; two-component system, LytTR family, response regulator | 0.663 | 1.26e-03 | 4.56e-02\* |
| tuaH; teichuronic acid biosynthesis glycosyltransferase TuaH [EC:2.4.-.-] | -2.409 | 1.29e-03 | 4.60e-02\* |
| bcrB, badE; benzoyl-CoA reductase subunit B [EC:1.3.7.8] | -1.296 | 1.29e-03 | 4.60e-02\* |
| rhaS; AraC family transcriptional regulator, L-rhamnose operon regulatory protein RhaS | 1.774 | 1.29e-03 | 4.60e-02\* |
| mutH; DNA mismatch repair protein MutH | 1.937 | 1.33e-03 | 4.69e-02\* |
| napD; periplasmic nitrate reductase NapD | -3.685 | 1.34e-03 | 4.69e-02\* |
| wbpP; UDP-N-acetylglucosamine 4-epimerase [EC:5.1.3.7] | 0.626 | 1.34e-03 | 4.69e-02\* |
| MFS.CP; MFS transporter, CP family, cyanate transporter | -2.253 | 1.40e-03 | 4.89e-02\* |
| bcsA; cellulose synthase (UDP-forming) [EC:2.4.1.12] | -1.711 | 1.43e-03 | 4.95e-02\* |
| fdtB; dTDP-3-amino-3,6-dideoxy-alpha-D-galactopyranose transaminase [EC:2.6.1.90] | -2.115 | 1.48e-03 | 5.11e-02 |
| menC; O-succinylbenzoate synthase [EC:4.2.1.113] | -1.196 | 1.51e-03 | 5.19e-02 |
| sucD; succinyl-CoA synthetase alpha subunit [EC:6.2.1.5] | -1.338 | 1.52e-03 | 5.20e-02 |
| ureE; urease accessory protein | -0.779 | 1.54e-03 | 5.22e-02 |
| K09706; uncharacterized protein | 2.193 | 1.55e-03 | 5.25e-02 |
| yfiQ; acetyltransferase | 1.073 | 1.57e-03 | 5.28e-02 |
| pat; phosphinothricin acetyltransferase [EC:2.3.1.183] | -0.982 | 1.58e-03 | 5.30e-02 |
| mscK, kefA, aefA; potassium-dependent mechanosensitive channel | -1.738 | 1.59e-03 | 5.30e-02 |
| lapE; outer membrane protein, adhesin transport system | 1.120 | 1.67e-03 | 5.45e-02 |
| hyaD, hybD; hydrogenase maturation protease [EC:3.4.23.-] | -1.849 | 1.67e-03 | 5.45e-02 |
| ydjE; MFS transporter, putative metabolite:H+ symporter | -1.749 | 1.67e-03 | 5.45e-02 |
| mexT; LysR family transcriptional regulator, mexEF-oprN operon transcriptional activator | -2.391 | 1.68e-03 | 5.46e-02 |
| frdA; fumarate reductase flavoprotein subunit [EC:1.3.5.4] | -0.626 | 1.69e-03 | 5.48e-02 |
| rsmB, sun; 16S rRNA (cytosine967-C5)-methyltransferase [EC:2.1.1.176] | -0.928 | 1.74e-03 | 5.56e-02 |
| hemA; glutamyl-tRNA reductase [EC:1.2.1.70] | -1.166 | 1.75e-03 | 5.56e-02 |
| traN; conjugal transfer mating pair stabilization protein TraN | -1.454 | 1.75e-03 | 5.56e-02 |
| K09992; uncharacterized protein | 1.051 | 1.78e-03 | 5.59e-02 |
| dinG; ATP-dependent DNA helicase DinG [EC:3.6.4.12] | -1.018 | 1.79e-03 | 5.59e-02 |
| PEO1; twinkle protein [EC:3.6.4.12] | 1.615 | 1.79e-03 | 5.59e-02 |
| pdxK, pdxY; pyridoxine kinase [EC:2.7.1.35] | 0.534 | 1.87e-03 | 5.83e-02 |
| utp; urea transporter | -0.691 | 1.89e-03 | 5.84e-02 |
| K07040; uncharacterized protein | -0.906 | 1.90e-03 | 5.84e-02 |
| dedD; DedD protein | -3.051 | 1.90e-03 | 5.84e-02 |
| K09155; uncharacterized protein | -2.291 | 2.01e-03 | 6.12e-02 |
| pseI, neuB3; pseudaminic acid synthase [EC:2.5.1.97] | 0.958 | 2.05e-03 | 6.21e-02 |
| E3.8.1.2; 2-haloacid dehalogenase [EC:3.8.1.2] | -1.553 | 2.07e-03 | 6.24e-02 |
| lldD; L-lactate dehydrogenase (cytochrome) [EC:1.1.2.3] | 2.404 | 2.14e-03 | 6.33e-02 |
| mnmC; tRNA 5-methylaminomethyl-2-thiouridine biosynthesis bifunctional protein [EC:2.1.1.61 1.5.-.-] | -1.496 | 2.16e-03 | 6.33e-02 |
| cslA; chondroitin AC lyase [EC:4.2.2.5] | 1.361 | 2.16e-03 | 6.33e-02 |
| rnhC; ribonuclease HIII [EC:3.1.26.4] | -3.772 | 2.17e-03 | 6.33e-02 |
| hscB, HSCB, HSC20; molecular chaperone HscB | -2.986 | 2.18e-03 | 6.33e-02 |
| pcaB; 3-carboxy-cis,cis-muconate cycloisomerase [EC:5.5.1.2] | -2.666 | 2.19e-03 | 6.33e-02 |
| rlmM; 23S rRNA (cytidine2498-2'-O)-methyltransferase [EC:2.1.1.186] | 3.140 | 2.19e-03 | 6.33e-02 |
| IAL; isopenicillin-N N-acyltransferase like protein | -2.533 | 2.22e-03 | 6.37e-02 |
| dadA; D-amino-acid dehydrogenase [EC:1.4.5.1] | -2.450 | 2.25e-03 | 6.43e-02 |
| rhtB; homoserine/homoserine lactone efflux protein | -2.449 | 2.28e-03 | 6.49e-02 |
| chpB, chpBK; mRNA interferase ChpB [EC:3.1.-.-] | -2.458 | 2.29e-03 | 6.50e-02 |
| cutC; copper homeostasis protein | 0.612 | 2.34e-03 | 6.60e-02 |
| rarD; chloramphenicol-sensitive protein RarD | -1.986 | 2.36e-03 | 6.64e-02 |
| nudH; putative (di)nucleoside polyphosphate hydrolase [EC:3.6.1.-] | -1.653 | 2.45e-03 | 6.80e-02 |
| traG; conjugal transfer mating pair stabilization protein TraG | -1.680 | 2.45e-03 | 6.80e-02 |
| thrB2; homoserine kinase type II [EC:2.7.1.39] | -1.876 | 2.46e-03 | 6.80e-02 |
| per, rfbE; perosamine synthetase [EC:2.6.1.102] | 0.806 | 2.48e-03 | 6.83e-02 |
| pbpB; penicillin-binding protein 2B | -1.727 | 2.55e-03 | 6.97e-02 |
| ttrR; two-component system, LuxR family, response regulator TtrR | -1.731 | 2.58e-03 | 6.99e-02 |
| iolW; scyllo-inositol 2-dehydrogenase (NADP+) [EC:1.1.1.371] | 1.095 | 2.61e-03 | 7.02e-02 |
| K17076, lysY; putative lysine transport system ATP-binding protein [EC:3.6.3.-] | -1.188 | 2.62e-03 | 7.02e-02 |
| queH; epoxyqueuosine reductase [EC:1.17.99.6] | 0.630 | 2.64e-03 | 7.03e-02 |
| RDH12; retinol dehydrogenase 12 [EC:1.1.1.300] | 1.098 | 2.65e-03 | 7.03e-02 |
| susA; neopullulanase [EC:3.2.1.135] | 0.823 | 2.68e-03 | 7.09e-02 |
| virB5, lvhB5; type IV secretion system protein VirB5 | -2.790 | 2.70e-03 | 7.10e-02 |
| ugl; unsaturated chondroitin disaccharide hydrolase [EC:3.2.1.180] | 0.782 | 2.72e-03 | 7.13e-02 |
| comFC; competence protein ComFC | -2.047 | 2.80e-03 | 7.30e-02 |
| K09974; uncharacterized protein | -3.099 | 2.83e-03 | 7.31e-02 |
| K07454; putative restriction endonuclease | 1.555 | 2.84e-03 | 7.31e-02 |
| bacC; dihydroanticapsin dehydrogenase [EC:1.1.1.385] | -1.185 | 2.84e-03 | 7.31e-02 |
| cysC; adenylylsulfate kinase [EC:2.7.1.25] | 0.760 | 2.88e-03 | 7.38e-02 |
| E3.5.2.10; creatinine amidohydrolase [EC:3.5.2.10] | 0.594 | 2.89e-03 | 7.38e-02 |
| RTCA, rtcA; RNA 3'-terminal phosphate cyclase (ATP) [EC:6.5.1.4] | -2.552 | 2.90e-03 | 7.39e-02 |
| wbiB; dTDP-L-rhamnose 4-epimerase [EC:5.1.3.25] | -1.737 | 2.93e-03 | 7.42e-02 |
| ascD, ddhD, rfbI; CDP-4-dehydro-6-deoxyglucose reductase, E3 [EC:1.17.1.1] | -2.180 | 2.95e-03 | 7.42e-02 |
| slmA, ttk; TetR/AcrR family transcriptional regulator | -1.892 | 2.97e-03 | 7.44e-02 |
| yueD; benzil reductase ((S)-benzoin forming) [EC:1.1.1.320] | -2.366 | 3.00e-03 | 7.47e-02 |
| trpGD; anthranilate synthase/phosphoribosyltransferase [EC:4.1.3.27 2.4.2.18] | -1.795 | 3.07e-03 | 7.57e-02 |
| metC; cysteine-S-conjugate beta-lyase [EC:4.4.1.13] | -0.727 | 3.08e-03 | 7.57e-02 |
| orn, REX2, REXO2; oligoribonuclease [EC:3.1.-.-] | -1.170 | 3.13e-03 | 7.62e-02 |
| aglJ; dolichol-phosphate hexosyltransferase [EC:2.4.1.-] | 1.197 | 3.14e-03 | 7.62e-02 |
| kch, trkA, mthK, pch; voltage-gated potassium channel | -0.640 | 3.15e-03 | 7.62e-02 |
| K06946; uncharacterized protein | 1.569 | 3.16e-03 | 7.62e-02 |
| flhF; flagellar biosynthesis protein FlhF | 1.210 | 3.21e-03 | 7.70e-02 |
| ABC.NGC.P; N-acetylglucosamine transport system permease protein | 0.956 | 3.27e-03 | 7.80e-02 |
| abgA; aminobenzoyl-glutamate utilization protein A | -0.975 | 3.34e-03 | 7.92e-02 |
| pldA; phospholipase A1/A2 [EC:3.1.1.32 3.1.1.4] | -0.589 | 3.35e-03 | 7.92e-02 |
| E5.2.1.8; peptidylprolyl isomerase [EC:5.2.1.8] | 0.895 | 3.41e-03 | 8.00e-02 |
| lysX1; putative lysine transport system substrate-binding protein | -1.634 | 3.41e-03 | 8.00e-02 |
| TC.DME; drug/metabolite transporter, DME family | -0.916 | 3.51e-03 | 8.18e-02 |
| lolA; outer membrane lipoprotein carrier protein | -1.556 | 3.58e-03 | 8.31e-02 |
| traE; conjugal transfer pilus assembly protein TraE | -2.594 | 3.63e-03 | 8.39e-02 |
| glcD; glycolate oxidase [EC:1.1.3.15] | -1.227 | 3.70e-03 | 8.53e-02 |
| prsA; foldase protein PrsA [EC:5.2.1.8] | -1.482 | 3.76e-03 | 8.62e-02 |
| hdhA; 7-alpha-hydroxysteroid dehydrogenase [EC:1.1.1.159] | -0.781 | 3.82e-03 | 8.72e-02 |
| rubB, alkT; rubredoxin---NAD+ reductase [EC:1.18.1.1] | 0.918 | 3.87e-03 | 8.81e-02 |
| cbiL; nickel transport protein | -2.401 | 4.14e-03 | 9.31e-02 |
| lacF; lactose PTS system EIIA component [EC:2.7.1.207] | -3.427 | 4.15e-03 | 9.31e-02 |
| K07487; transposase | 1.253 | 4.16e-03 | 9.31e-02 |
| kdpB; potassium-transporting ATPase ATP-binding subunit [EC:7.2.2.6] | -1.531 | 4.16e-03 | 9.31e-02 |
| pgl; 6-phosphogluconolactonase [EC:3.1.1.31] | 0.783 | 4.18e-03 | 9.32e-02 |
| tamB; translocation and assembly module TamB | -0.641 | 4.22e-03 | 9.38e-02 |
| E3.1.11.5; exodeoxyribonuclease V [EC:3.1.11.5] | 0.877 | 4.24e-03 | 9.39e-02 |
| ohrR; MarR family transcriptional regulator, organic hydroperoxide resistance regulator | 0.671 | 4.33e-03 | 9.56e-02 |
| MACROD, ymdB; O-acetyl-ADP-ribose deacetylase [EC:3.1.1.106] | -0.619 | 4.34e-03 | 9.56e-02 |
| TC.PST; polysaccharide transporter, PST family | -1.034 | 4.43e-03 | 9.72e-02 |
| tatB; sec-independent protein translocase protein TatB | -1.104 | 4.48e-03 | 9.80e-02 |
| ycsE, yitU, ywtE; 5-amino-6-(5-phospho-D-ribitylamino)uracil phosphatase [EC:3.1.3.104] | -0.717 | 4.53e-03 | 9.83e-02 |
| ehbD; energy-converting hydrogenase B subunit D | 1.039 | 4.53e-03 | 9.83e-02 |
| K09963; uncharacterized protein | -1.145 | 4.54e-03 | 9.83e-02 |
| mexJ; membrane fusion protein, multidrug efflux system | 0.788 | 4.56e-03 | 9.83e-02 |
| pmbA; PmbA protein | 0.821 | 4.68e-03 | 1.01e-01 |
| waaZ, rfaZ; KDO transferase III [EC:2.4.99.-] | -2.631 | 4.70e-03 | 1.01e-01 |
| E3.4.21.66; thermitase [EC:3.4.21.66] | 1.492 | 4.71e-03 | 1.01e-01 |
| oadG; oxaloacetate decarboxylase (Na+ extruding) subunit gamma | 1.135 | 4.82e-03 | 1.03e-01 |
| rsbU\_P; phosphoserine phosphatase RsbU/P [EC:3.1.3.3] | -0.771 | 4.97e-03 | 1.05e-01 |
| glnL, ntrB; two-component system, NtrC family, nitrogen regulation sensor histidine kinase GlnL [EC:2.7.13.3] | -1.558 | 4.97e-03 | 1.05e-01 |
| cobA-hemD; uroporphyrinogen III methyltransferase / synthase [EC:2.1.1.107 4.2.1.75] | -1.104 | 5.01e-03 | 1.05e-01 |
| rluA; tRNA pseudouridine32 synthase / 23S rRNA pseudouridine746 synthase [EC:5.4.99.28 5.4.99.29] | 0.944 | 5.02e-03 | 1.05e-01 |
| mupP; N-acetyl-D-muramate 6-phosphate phosphatase [EC:3.1.3.105] | -1.751 | 5.02e-03 | 1.05e-01 |
| kptA; putative RNA 2'-phosphotransferase [EC:2.7.1.-] | 0.832 | 5.02e-03 | 1.05e-01 |
| dcd; dCTP deaminase [EC:3.5.4.13] | -0.996 | 5.08e-03 | 1.05e-01 |
| higA; HTH-type transcriptional regulator / antitoxin HigA | 0.647 | 5.09e-03 | 1.05e-01 |
| trcR; two-component system, OmpR family, response regulator TrcR | 0.941 | 5.17e-03 | 1.06e-01 |
| K07497; putative transposase | 0.553 | 5.17e-03 | 1.06e-01 |
| hisE; phosphoribosyl-ATP pyrophosphohydrolase [EC:3.6.1.31] | -1.057 | 5.36e-03 | 1.09e-01 |
| fliR; flagellar biosynthetic protein FliR | 1.064 | 5.43e-03 | 1.10e-01 |
| ATPVE, ntpE, atpE; V/A-type H+/Na+-transporting ATPase subunit E | 0.636 | 5.45e-03 | 1.10e-01 |
| gfrB; fructoselysine/glucoselysine PTS system EIIB component [EC:2.7.1.-] | -1.402 | 5.53e-03 | 1.11e-01 |
| K09928; uncharacterized protein | -2.109 | 5.63e-03 | 1.13e-01 |
| tetA; MFS transporter, DHA1 family, tetracycline resistance protein | -1.808 | 5.68e-03 | 1.13e-01 |
| apaG; ApaG protein | -4.548 | 5.76e-03 | 1.14e-01 |
| cbpA; curved DNA-binding protein | 0.525 | 5.77e-03 | 1.14e-01 |
| comFA; competence protein ComFA | -2.299 | 5.77e-03 | 1.14e-01 |
| dexA; dextranase [EC:3.2.1.11] | 0.731 | 5.78e-03 | 1.14e-01 |
| oppA, mppA; oligopeptide transport system substrate-binding protein | -0.867 | 5.98e-03 | 1.18e-01 |
| hslV, clpQ; ATP-dependent HslUV protease, peptidase subunit HslV [EC:3.4.25.2] | 0.754 | 6.02e-03 | 1.18e-01 |
| nrfG; formate-dependent nitrite reductase complex subunit NrfG | -2.669 | 6.02e-03 | 1.18e-01 |
| ganQ; arabinogalactan oligomer / maltooligosaccharide transport system permease protein | 0.916 | 6.05e-03 | 1.18e-01 |
| purQ; phosphoribosylformylglycinamidine synthase subunit PurQ / glutaminase [EC:6.3.5.3 3.5.1.2] | -1.722 | 6.07e-03 | 1.18e-01 |
| caiA; crotonobetainyl-CoA dehydrogenase [EC:1.3.8.13] | 1.436 | 6.19e-03 | 1.20e-01 |
| mdcR; LysR family transcriptional regulator, malonate utilization transcriptional regulator | -1.968 | 6.20e-03 | 1.20e-01 |
| panF; sodium/pantothenate symporter | 1.349 | 6.23e-03 | 1.20e-01 |
| cobL; precorrin-6Y C5,15-methyltransferase (decarboxylating) [EC:2.1.1.132] | 0.660 | 6.37e-03 | 1.22e-01 |
| TC.DASS; divalent anion:Na+ symporter, DASS family | -0.901 | 6.38e-03 | 1.22e-01 |
| trbI; conjugal transfer pilin signal peptidase TrbI | -1.885 | 6.40e-03 | 1.22e-01 |
| nicX; 2,5-dihydroxypyridine 5,6-dioxygenase [EC:1.13.11.9] | -1.087 | 6.46e-03 | 1.22e-01 |
| glpG; GlpG protein | -2.138 | 6.59e-03 | 1.25e-01 |
| K03710; GntR family transcriptional regulator | -0.838 | 6.62e-03 | 1.25e-01 |
| amgK; N-acetylmuramate 1-kinase [EC:2.7.1.221] | -0.778 | 6.64e-03 | 1.25e-01 |
| chuW; anaerobilin synthase [EC:2.1.1.342] | -1.048 | 6.67e-03 | 1.25e-01 |
| nosF; Cu-processing system ATP-binding protein | -2.269 | 6.67e-03 | 1.25e-01 |
| ykoE; energy-coupling factor transport system substrate-specific component | -2.031 | 6.74e-03 | 1.26e-01 |
| K00243; uncharacterized protein | 0.534 | 6.80e-03 | 1.26e-01 |
| amn; AMP nucleosidase [EC:3.2.2.4] | 0.701 | 6.81e-03 | 1.26e-01 |
| raxA; membrane fusion protein | 1.216 | 6.88e-03 | 1.27e-01 |
| PGLS, pgl, devB; 6-phosphogluconolactonase [EC:3.1.1.31] | 0.974 | 6.97e-03 | 1.28e-01 |
| afr; 1,5-anhydro-D-fructose reductase (1,5-anhydro-D-mannitol-forming) [EC:1.1.1.292] | 0.888 | 7.06e-03 | 1.30e-01 |
| rsbV; anti-sigma B factor antagonist | -1.920 | 7.13e-03 | 1.31e-01 |
| plc; 1-phosphatidylinositol phosphodiesterase [EC:4.6.1.13] | 1.170 | 7.18e-03 | 1.31e-01 |
| mtiP; 5'-methylthioinosine phosphorylase [EC:2.4.2.44] | -2.319 | 7.48e-03 | 1.36e-01 |
| traF; conjugal transfer pilus assembly protein TraF | -2.395 | 7.51e-03 | 1.36e-01 |
| comGC; competence protein ComGC | -1.800 | 7.53e-03 | 1.36e-01 |
| dpe, lre; D-psicose/D-tagatose/L-ribulose 3-epimerase [EC:5.1.3.30 5.1.3.31] | 1.105 | 7.54e-03 | 1.36e-01 |
| E1.1.1.67, mtlK; mannitol 2-dehydrogenase [EC:1.1.1.67] | 1.015 | 7.78e-03 | 1.40e-01 |
| czcB, cusB, cnrB; membrane fusion protein, heavy metal efflux system | -1.035 | 7.80e-03 | 1.40e-01 |
| gmhD, rfaD; ADP-L-glycero-D-manno-heptose 6-epimerase [EC:5.1.3.20] | -1.357 | 7.84e-03 | 1.40e-01 |
| algD; GDP-mannose 6-dehydrogenase [EC:1.1.1.132] | -1.140 | 7.85e-03 | 1.40e-01 |
| K09133; uncharacterized protein | -0.805 | 7.89e-03 | 1.41e-01 |
| splB; spore photoproduct lyase [EC:4.1.99.14] | 1.116 | 7.92e-03 | 1.41e-01 |
| gumF; acyltransferase [EC:2.3.1.-] | -0.951 | 7.94e-03 | 1.41e-01 |
| PC, pyc; pyruvate carboxylase [EC:6.4.1.1] | 0.520 | 7.98e-03 | 1.41e-01 |
| phoH2; PhoH-like ATPase | 0.763 | 8.05e-03 | 1.42e-01 |
| czcD, zitB; cobalt-zinc-cadmium efflux system protein | -0.560 | 8.10e-03 | 1.42e-01 |
| ABC.ZM.S; zinc/manganese transport system substrate-binding protein | -1.754 | 8.11e-03 | 1.42e-01 |
| ftsL; cell division protein FtsL | -3.020 | 8.12e-03 | 1.42e-01 |
| ykfC; gamma-D-glutamyl-L-lysine dipeptidyl-peptidase [EC:3.4.14.13] | -0.533 | 8.21e-03 | 1.43e-01 |
| flgH; flagellar L-ring protein precursor FlgH | 1.560 | 8.22e-03 | 1.43e-01 |
| cstA; carbon starvation protein | -0.552 | 8.24e-03 | 1.43e-01 |
| yefM; antitoxin YefM | 0.886 | 8.27e-03 | 1.43e-01 |
| rtcR; transcriptional regulatory protein RtcR | -1.757 | 8.33e-03 | 1.44e-01 |
| hyaC; Ni/Fe-hydrogenase 1 B-type cytochrome subunit | -1.149 | 8.34e-03 | 1.44e-01 |
| ABCC-BAC; ATP-binding cassette, subfamily C, bacterial | 1.120 | 8.44e-03 | 1.45e-01 |
| cssR; two-component system, OmpR family, response regulator CssR | -1.906 | 8.47e-03 | 1.45e-01 |
| ygfZ; tRNA-modifying protein YgfZ | -1.984 | 8.48e-03 | 1.45e-01 |
| comGD; competence protein ComGD | -3.522 | 8.49e-03 | 1.45e-01 |
| kdpD; two-component system, OmpR family, sensor histidine kinase KdpD [EC:2.7.13.3] | -1.306 | 8.78e-03 | 1.48e-01 |
| mdlB, smdB; ATP-binding cassette, subfamily B, multidrug efflux pump | 0.869 | 8.79e-03 | 1.48e-01 |
| fdhE; FdhE protein | -1.497 | 8.99e-03 | 1.51e-01 |
| TC.CNT; concentrative nucleoside transporter, CNT family | 0.924 | 9.07e-03 | 1.52e-01 |
| CRLS; cardiolipin synthase (CMP-forming) [EC:2.7.8.41] | -1.660 | 9.19e-03 | 1.52e-01 |
| icmB, dotO; intracellular multiplication protein IcmB [EC:7.2.4.8] | 1.068 | 9.19e-03 | 1.52e-01 |
| tldD; TldD protein | 0.574 | 9.22e-03 | 1.52e-01 |
| cutF, nlpE; copper homeostasis protein (lipoprotein) | -2.909 | 9.26e-03 | 1.53e-01 |
| msrAB; peptide methionine sulfoxide reductase msrA/msrB [EC:1.8.4.11 1.8.4.12] | -0.598 | 9.31e-03 | 1.53e-01 |
| nagK; fumarylpyruvate hydrolase [EC:3.7.1.20] | 0.902 | 9.50e-03 | 1.54e-01 |
| recB; exodeoxyribonuclease V beta subunit [EC:3.1.11.5] | -1.490 | 9.52e-03 | 1.54e-01 |
| clpS; ATP-dependent Clp protease adaptor protein ClpS | 0.790 | 9.52e-03 | 1.54e-01 |
| hslU; ATP-dependent HslUV protease ATP-binding subunit HslU | 0.553 | 9.58e-03 | 1.54e-01 |
| appA; 4-phytase / acid phosphatase [EC:3.1.3.26 3.1.3.2] | -1.199 | 9.61e-03 | 1.54e-01 |
| dsbB; protein dithiol:quinone oxidoreductase [EC:1.8.5.9] | -1.909 | 9.62e-03 | 1.54e-01 |
| fk; tagatose kinase [EC:2.7.1.101] | -2.468 | 9.63e-03 | 1.54e-01 |
| prlC; oligopeptidase A [EC:3.4.24.70] | -0.718 | 9.65e-03 | 1.54e-01 |
| K07000; uncharacterized protein | -1.392 | 9.71e-03 | 1.54e-01 |
| linN; cholesterol transport system auxiliary component | -2.078 | 9.75e-03 | 1.54e-01 |
| pspE; phage shock protein E | -1.313 | 9.75e-03 | 1.54e-01 |
| mmsA, iolA, ALDH6A1; malonate-semialdehyde dehydrogenase (acetylating) / methylmalonate-semialdehyde dehydrogenase [EC:1.2.1.18 1.2.1.27] | 0.885 | 9.76e-03 | 1.54e-01 |
| modD; molybdenum transport protein [EC:2.4.2.-] | -1.722 | 9.77e-03 | 1.54e-01 |
| qmoB; quinone-modifying oxidoreductase, subunit QmoB | 1.323 | 9.98e-03 | 1.57e-01 |
| lptE, rlpB; LPS-assembly lipoprotein | -2.088 | 1.03e-02 | 1.61e-01 |
| K06993; ribonuclease H-related protein | 1.407 | 1.05e-02 | 1.63e-01 |
| bcsB; cellulose synthase operon protein B | -0.988 | 1.05e-02 | 1.63e-01 |
| hasA; hyaluronan synthase [EC:2.4.1.212] | 3.589 | 1.05e-02 | 1.63e-01 |
| K07506; AraC family transcriptional regulator | 0.923 | 1.05e-02 | 1.63e-01 |
| pycA; pyruvate carboxylase subunit A [EC:6.4.1.1] | 1.607 | 1.06e-02 | 1.63e-01 |
| dbpA; ATP-dependent RNA helicase DbpA [EC:3.6.4.13] | -0.755 | 1.06e-02 | 1.63e-01 |
| fliC; flagellin | 0.789 | 1.08e-02 | 1.64e-01 |
| cimA; (R)-citramalate synthase [EC:2.3.1.182] | 0.568 | 1.08e-02 | 1.64e-01 |
| ihfB, himD; integration host factor subunit beta | -1.771 | 1.08e-02 | 1.64e-01 |
| fmtA; teichoic acid D-alanine hydrolase [EC:3.1.1.103] | -1.346 | 1.08e-02 | 1.64e-01 |
| helY; ATP-dependent RNA helicase HelY [EC:3.6.4.-] | -4.939 | 1.08e-02 | 1.64e-01 |
| fabV, ter; enoyl-[acyl-carrier protein] reductase / trans-2-enoyl-CoA reductase (NAD+) [EC:1.3.1.9 1.3.1.44] | -1.434 | 1.09e-02 | 1.64e-01 |
| pglH; GalNAc-alpha-(1->4)-GalNAc-alpha-(1->3)-diNAcBac-PP-undecaprenol alpha-1,4-N-acetyl-D-galactosaminyltransferase [EC:2.4.1.292] | 2.533 | 1.09e-02 | 1.65e-01 |
| MET8; precorrin-2 dehydrogenase / sirohydrochlorin ferrochelatase [EC:1.3.1.76 4.99.1.4] | -3.042 | 1.11e-02 | 1.67e-01 |
| rlpA; rare lipoprotein A | -1.242 | 1.11e-02 | 1.67e-01 |
| hypF; hydrogenase maturation protein HypF | -1.167 | 1.12e-02 | 1.68e-01 |
| xylF; D-xylose transport system substrate-binding protein | 0.835 | 1.12e-02 | 1.68e-01 |
| ezrA; septation ring formation regulator | -1.669 | 1.12e-02 | 1.68e-01 |
| pbpA; penicillin-binding protein A | -1.356 | 1.13e-02 | 1.68e-01 |
| yggT; YggT family protein | -1.031 | 1.13e-02 | 1.68e-01 |
| K06884; uncharacterized protein | -1.937 | 1.14e-02 | 1.69e-01 |
| mepM; murein DD-endopeptidase [EC:3.4.24.-] | 0.716 | 1.14e-02 | 1.69e-01 |
| TREH, treA, treF; alpha,alpha-trehalase [EC:3.2.1.28] | 1.358 | 1.16e-02 | 1.70e-01 |
| MAN2C1; alpha-mannosidase [EC:3.2.1.24] | 0.684 | 1.16e-02 | 1.70e-01 |
| asp1; accessory secretory protein Asp1 | -0.961 | 1.16e-02 | 1.70e-01 |
| dmsC; dimethyl sulfoxide reductase membrane subunit | -1.113 | 1.16e-02 | 1.71e-01 |
| ACR3, arsB; arsenite transporter | -1.264 | 1.18e-02 | 1.72e-01 |
| thrH; phosphoserine / homoserine phosphotransferase [EC:3.1.3.3 2.7.1.39] | 0.721 | 1.18e-02 | 1.72e-01 |
| cusR, copR, silR; two-component system, OmpR family, copper resistance phosphate regulon response regulator CusR | 1.297 | 1.18e-02 | 1.72e-01 |
| rimI; [ribosomal protein S18]-alanine N-acetyltransferase [EC:2.3.1.266] | -0.884 | 1.19e-02 | 1.73e-01 |
| csn1, cas9; CRISPR-associated endonuclease Csn1 [EC:3.1.-.-] | -0.732 | 1.19e-02 | 1.73e-01 |
| glgE; starch synthase (maltosyl-transferring) [EC:2.4.99.16] | -1.262 | 1.21e-02 | 1.75e-01 |
| relB; RHH-type transcriptional regulator, rel operon repressor / antitoxin RelB | -1.996 | 1.23e-02 | 1.76e-01 |
| nupC; nucleoside transport protein | 0.827 | 1.23e-02 | 1.76e-01 |
| K09939; uncharacterized protein | 1.942 | 1.24e-02 | 1.77e-01 |
| ndh; NADH dehydrogenase [EC:1.6.99.3] | -0.805 | 1.25e-02 | 1.77e-01 |
| virB3, lvhB3; type IV secretion system protein VirB3 | -2.414 | 1.25e-02 | 1.78e-01 |
| rfbF, rhlC; rhamnosyltransferase [EC:2.4.1.-] | -3.806 | 1.26e-02 | 1.79e-01 |
| nodU; carbamoyltransferase [EC:2.1.3.-] | 0.852 | 1.26e-02 | 1.79e-01 |
| sbp; sulfate/thiosulfate transport system substrate-binding protein | 1.870 | 1.28e-02 | 1.80e-01 |
| K10121, msmF; fructooligosaccharide transport system permease protein | -1.000 | 1.29e-02 | 1.80e-01 |
| K06904; uncharacterized protein | 1.030 | 1.30e-02 | 1.81e-01 |
| mnhC, mrpC; multicomponent Na+:H+ antiporter subunit C | 0.837 | 1.30e-02 | 1.81e-01 |
| lytS; two-component system, LytTR family, sensor histidine kinase LytS [EC:2.7.13.3] | -1.096 | 1.31e-02 | 1.81e-01 |
| menH; 2-succinyl-6-hydroxy-2,4-cyclohexadiene-1-carboxylate synthase [EC:4.2.99.20] | -2.342 | 1.32e-02 | 1.81e-01 |
| K15667, ppsD, fenA; fengycin family lipopeptide synthetase D | 2.555 | 1.32e-02 | 1.81e-01 |
| menF; menaquinone-specific isochorismate synthase [EC:5.4.4.2] | -1.273 | 1.32e-02 | 1.81e-01 |
| tolC; outer membrane protein | 0.545 | 1.32e-02 | 1.81e-01 |
| efrA; ATP-binding cassette, subfamily B, multidrug efflux pump | 1.684 | 1.32e-02 | 1.81e-01 |
| glgM; alpha-maltose-1-phosphate synthase [EC:2.4.1.342] | -3.979 | 1.32e-02 | 1.81e-01 |
| lysX2; putative lysine transport system permease protein | -1.173 | 1.33e-02 | 1.81e-01 |
| hyaB, hybC; hydrogenase large subunit [EC:1.12.99.6] | -0.991 | 1.34e-02 | 1.82e-01 |
| araC; AraC family transcriptional regulator, arabinose operon regulatory protein | 0.743 | 1.34e-02 | 1.82e-01 |
| E2.4.1.333; 1,2-beta-oligoglucan phosphorylase [EC:2.4.1.333] | 2.200 | 1.37e-02 | 1.85e-01 |
| djlA; DnaJ like chaperone protein | 0.654 | 1.38e-02 | 1.87e-01 |
| nosD; nitrous oxidase accessory protein | -1.714 | 1.40e-02 | 1.89e-01 |
| folE2; GTP cyclohydrolase IB [EC:3.5.4.16] | -1.447 | 1.42e-02 | 1.90e-01 |
| tagT\_U\_V; polyisoprenyl-teichoic acid--peptidoglycan teichoic acid transferase [EC:2.7.8.-] | -0.835 | 1.42e-02 | 1.90e-01 |
| bshB1; N-acetylglucosamine malate deacetylase 1 [EC:3.5.1.-] | 1.248 | 1.42e-02 | 1.90e-01 |
| mdtB; multidrug efflux pump | 0.873 | 1.43e-02 | 1.91e-01 |
| cbe, mbe; cellobiose epimerase [EC:5.1.3.11] | 0.573 | 1.44e-02 | 1.92e-01 |
| araE; MFS transporter, SP family, arabinose:H+ symporter | 0.686 | 1.44e-02 | 1.92e-01 |
| hdeD; membrane protein HdeD | 0.934 | 1.45e-02 | 1.93e-01 |
| menE; O-succinylbenzoic acid---CoA ligase [EC:6.2.1.26] | -0.727 | 1.46e-02 | 1.93e-01 |
| hmp, YHB1; nitric oxide dioxygenase [EC:1.14.12.17] | -1.760 | 1.48e-02 | 1.94e-01 |
| ABC-2.LPSE.P; lipopolysaccharide transport system permease protein | 1.302 | 1.49e-02 | 1.96e-01 |
| E2.4.1.7; sucrose phosphorylase [EC:2.4.1.7] | -0.849 | 1.50e-02 | 1.97e-01 |
| nosY; Cu-processing system permease protein | -2.316 | 1.51e-02 | 1.97e-01 |
| aroD; 3-dehydroquinate dehydratase I [EC:4.2.1.10] | -0.925 | 1.51e-02 | 1.97e-01 |
| chbG; chitin disaccharide deacetylase [EC:3.5.1.105] | -2.667 | 1.52e-02 | 1.97e-01 |
| rclR; AraC family transcriptional regulator, reactive chlorine species (RCS)-specific activator of rcl operon | -1.261 | 1.52e-02 | 1.97e-01 |
| trmK; tRNA (adenine22-N1)-methyltransferase [EC:2.1.1.217] | -1.275 | 1.54e-02 | 1.99e-01 |
| K09921; uncharacterized protein | -1.612 | 1.55e-02 | 1.99e-01 |
| macB; macrolide transport system ATP-binding/permease protein [EC:3.6.3.-] | 0.867 | 1.55e-02 | 1.99e-01 |
| fliA; RNA polymerase sigma factor for flagellar operon FliA | 0.723 | 1.55e-02 | 1.99e-01 |
| speE, SRM, SPE3; spermidine synthase [EC:2.5.1.16] | 0.939 | 1.57e-02 | 2.01e-01 |
| hisI; phosphoribosyl-AMP cyclohydrolase [EC:3.5.4.19] | -1.042 | 1.57e-02 | 2.01e-01 |
| cpt; chloramphenicol 3-O phosphotransferase [EC:2.7.1.-] | -1.735 | 1.59e-02 | 2.03e-01 |
| gluA; glutamate transport system ATP-binding protein [EC:7.4.2.1] | -3.948 | 1.60e-02 | 2.05e-01 |
| ABC.NGC.S; N-acetylglucosamine transport system substrate-binding protein | 0.787 | 1.61e-02 | 2.06e-01 |
| K03791; putative chitinase | 2.488 | 1.63e-02 | 2.07e-01 |
| tet; tetrahedral aminopeptidase [EC:3.4.11.-] | -4.626 | 1.63e-02 | 2.07e-01 |
| ndk, NME; nucleoside-diphosphate kinase [EC:2.7.4.6] | -0.565 | 1.64e-02 | 2.07e-01 |
| K09940; uncharacterized protein | -5.229 | 1.65e-02 | 2.07e-01 |
| K06962; uncharacterized protein | 0.858 | 1.65e-02 | 2.07e-01 |
| K07491; putative transposase | 0.742 | 1.66e-02 | 2.08e-01 |
| murJ, mviN; putative peptidoglycan lipid II flippase | -1.258 | 1.66e-02 | 2.08e-01 |
| gfrC; fructoselysine/glucoselysine PTS system EIIC component | -1.281 | 1.67e-02 | 2.09e-01 |
| K13652; AraC family transcriptional regulator | 1.022 | 1.68e-02 | 2.09e-01 |
| agrB; accessory gene regulator B | 1.671 | 1.71e-02 | 2.12e-01 |
| triA; membrane fusion protein, multidrug efflux system | 1.420 | 1.71e-02 | 2.12e-01 |
| truC; tRNA pseudouridine65 synthase [EC:5.4.99.26] | -1.417 | 1.73e-02 | 2.13e-01 |
| NRT, narK, nrtP, nasA; MFS transporter, NNP family, nitrate/nitrite transporter | -0.518 | 1.76e-02 | 2.16e-01 |
| aphA; kanamycin kinase [EC:2.7.1.95] | -2.157 | 1.76e-02 | 2.16e-01 |
| gmhB; D-glycero-D-manno-heptose 1,7-bisphosphate phosphatase [EC:3.1.3.82 3.1.3.83] | -1.233 | 1.78e-02 | 2.18e-01 |
| inlA; internalin A | -1.638 | 1.80e-02 | 2.19e-01 |
| sstT; serine/threonine transporter | -0.753 | 1.81e-02 | 2.20e-01 |
| mhpE; 4-hydroxy 2-oxovalerate aldolase [EC:4.1.3.39] | 0.657 | 1.82e-02 | 2.21e-01 |
| ureAB; urease subunit gamma/beta [EC:3.5.1.5] | 1.188 | 1.82e-02 | 2.21e-01 |
| TRM61, GCD14; tRNA (adenine57-N1/adenine58-N1)-methyltransferase catalytic subunit [EC:2.1.1.219 2.1.1.220] | -2.259 | 1.83e-02 | 2.21e-01 |
| comGA; competence protein ComGA | -2.427 | 1.83e-02 | 2.21e-01 |
| pepD; putative serine protease PepD [EC:3.4.21.-] | -1.551 | 1.84e-02 | 2.22e-01 |
| aprX; serine protease AprX [EC:3.4.21.-] | -0.745 | 1.86e-02 | 2.22e-01 |
| argO; N-acetylglutamate synthase [EC:2.3.1.1] | 0.739 | 1.86e-02 | 2.22e-01 |
| virB6, lvhB6; type IV secretion system protein VirB6 | -2.235 | 1.87e-02 | 2.22e-01 |
| acoB; acetoin:2,6-dichlorophenolindophenol oxidoreductase subunit beta [EC:1.1.1.-] | -1.542 | 1.87e-02 | 2.22e-01 |
| tyrB; aromatic-amino-acid transaminase [EC:2.6.1.57] | -0.987 | 1.87e-02 | 2.22e-01 |
| arcD, lysl, lysP; arginine:ornithine antiporter / lysine permease | -0.844 | 1.87e-02 | 2.22e-01 |
| K15977; putative oxidoreductase | -0.670 | 1.89e-02 | 2.24e-01 |
| queG; epoxyqueuosine reductase [EC:1.17.99.6] | -0.565 | 1.89e-02 | 2.24e-01 |
| ppx-gppA; exopolyphosphatase / guanosine-5'-triphosphate,3'-diphosphate pyrophosphatase [EC:3.6.1.11 3.6.1.40] | -0.923 | 1.89e-02 | 2.24e-01 |
| ENGASE; mannosyl-glycoprotein endo-beta-N-acetylglucosaminidase [EC:3.2.1.96] | -0.751 | 1.90e-02 | 2.24e-01 |
| lgaB; L-galactono-1,5-lactonase [EC:3.1.1.-] | -0.732 | 1.92e-02 | 2.26e-01 |
| E3.2.1.197; beta-1,2-mannosidase [EC:3.2.1.197] | 0.858 | 1.93e-02 | 2.26e-01 |
| cgeB; spore maturation protein CgeB | 1.043 | 1.93e-02 | 2.26e-01 |
| pdhR; GntR family transcriptional regulator, transcriptional repressor for pyruvate dehydrogenase complex | -0.623 | 1.98e-02 | 2.31e-01 |
| moaA, CNX2; GTP 3',8-cyclase [EC:4.1.99.22] | -0.641 | 1.98e-02 | 2.31e-01 |
| HSPA1s; heat shock 70kDa protein 1/2/6/8 | -0.997 | 1.98e-02 | 2.31e-01 |
| dusA; tRNA-dihydrouridine synthase A [EC:1.-.-.-] | -0.807 | 1.99e-02 | 2.32e-01 |
| ereA\_B; erythromycin esterase [EC:3.1.1.-] | 1.454 | 2.01e-02 | 2.33e-01 |
| mdtJ; spermidine export protein MdtJ | 2.657 | 2.02e-02 | 2.34e-01 |
| cpxP, spy; periplasmic protein CpxP/Spy | -0.866 | 2.03e-02 | 2.34e-01 |
| K07017; uncharacterized protein | 0.925 | 2.04e-02 | 2.34e-01 |
| ALDO; fructose-bisphosphate aldolase, class I [EC:4.1.2.13] | -2.329 | 2.04e-02 | 2.34e-01 |
| addB; ATP-dependent helicase/nuclease subunit B [EC:3.1.-.- 3.6.4.12] | -0.526 | 2.08e-02 | 2.37e-01 |
| ispZ; intracellular septation protein | -1.230 | 2.08e-02 | 2.37e-01 |
| TST, MPST, sseA; thiosulfate/3-mercaptopyruvate sulfurtransferase [EC:2.8.1.1 2.8.1.2] | -1.708 | 2.09e-02 | 2.37e-01 |
| menJ; menaquinone-9 beta-reductase [EC:1.3.99.38] | 0.988 | 2.11e-02 | 2.39e-01 |
| lacE, araN; lactose/L-arabinose transport system substrate-binding protein | 0.940 | 2.11e-02 | 2.39e-01 |
| gatB, sgcB; galactitol PTS system EIIB component [EC:2.7.1.200] | -1.033 | 2.13e-02 | 2.41e-01 |
| aapJ, bztA; general L-amino acid transport system substrate-binding protein | -0.726 | 2.13e-02 | 2.41e-01 |
| agrA, blpR, fsrA; two-component system, LytTR family, response regulator AgrA | 0.830 | 2.14e-02 | 2.41e-01 |
| helD; DNA helicase IV [EC:3.6.4.12] | -0.548 | 2.16e-02 | 2.43e-01 |
| bapA; large repetitive protein | -0.636 | 2.17e-02 | 2.43e-01 |
| gfrF; fructoselysine-6-phosphate deglycase | -0.858 | 2.17e-02 | 2.43e-01 |
| sbcB, exoI; exodeoxyribonuclease I [EC:3.1.11.1] | -1.496 | 2.20e-02 | 2.45e-01 |
| acoA; acetoin:2,6-dichlorophenolindophenol oxidoreductase subunit alpha [EC:1.1.1.-] | -1.170 | 2.20e-02 | 2.45e-01 |
| K06934; uncharacterized protein | -2.185 | 2.21e-02 | 2.45e-01 |
| casD, cse5; CRISPR system Cascade subunit CasD | -4.258 | 2.21e-02 | 2.45e-01 |
| ntrY; two-component system, NtrC family, nitrogen regulation sensor histidine kinase NtrY [EC:2.7.13.3] | 0.896 | 2.21e-02 | 2.45e-01 |
| ALG14; beta-1,4-N-acetylglucosaminyltransferase [EC:2.4.1.141] | -0.915 | 2.22e-02 | 2.46e-01 |
| pbpG; serine-type D-Ala-D-Ala endopeptidase (penicillin-binding protein 7) [EC:3.4.21.-] | -0.998 | 2.23e-02 | 2.46e-01 |
| glnH; glutamine transport system substrate-binding protein | -0.913 | 2.23e-02 | 2.46e-01 |
| atpI; ATP synthase protein I | -1.580 | 2.24e-02 | 2.46e-01 |
| bglB; beta-glucosidase [EC:3.2.1.21] | 0.707 | 2.24e-02 | 2.46e-01 |
| rclA; probable pyridine nucleotide-disulfide oxidoreductase | 0.605 | 2.25e-02 | 2.47e-01 |
| hypD; hydrogenase expression/formation protein HypD | -0.824 | 2.26e-02 | 2.47e-01 |
| celC, chbA; cellobiose PTS system EIIA component [EC:2.7.1.196 2.7.1.205] | -0.860 | 2.26e-02 | 2.47e-01 |
| dnaB; replication initiation and membrane attachment protein | -1.713 | 2.26e-02 | 2.47e-01 |
| mntH; manganese transport protein | 0.501 | 2.27e-02 | 2.47e-01 |
| ada; AraC family transcriptional regulator, regulatory protein of adaptative response / methylated-DNA-[protein]-cysteine methyltransferase [EC:2.1.1.63] | -1.261 | 2.28e-02 | 2.48e-01 |
| gmhC, hldE, waaE, rfaE; D-beta-D-heptose 7-phosphate kinase / D-beta-D-heptose 1-phosphate adenosyltransferase [EC:2.7.1.167 2.7.7.70] | -1.665 | 2.33e-02 | 2.52e-01 |
| E1.17.4.1B, nrdB, nrdF; ribonucleoside-diphosphate reductase beta chain [EC:1.17.4.1] | -0.826 | 2.33e-02 | 2.52e-01 |
| osmB; osmotically inducible lipoprotein OsmB | 2.090 | 2.33e-02 | 2.52e-01 |
| agaD; galactosamine PTS system EIID component | -1.328 | 2.35e-02 | 2.53e-01 |
| emrA; membrane fusion protein, multidrug efflux system | 0.845 | 2.35e-02 | 2.53e-01 |
| lgaC; L-galactonate 5-dehydrogenase [EC:1.1.1.414] | -0.727 | 2.36e-02 | 2.53e-01 |
| waaL, rfaL; O-antigen ligase [EC:2.4.1.-] | -1.437 | 2.36e-02 | 2.53e-01 |
| dndB; DNA sulfur modification protein DndB | 1.475 | 2.37e-02 | 2.53e-01 |
| nosL; copper chaperone NosL | -1.240 | 2.39e-02 | 2.55e-01 |
| arnC, pmrF; undecaprenyl-phosphate 4-deoxy-4-formamido-L-arabinose transferase [EC:2.4.2.53] | 0.890 | 2.40e-02 | 2.56e-01 |
| aepZ; 2-aminoethylphosphonate-pyruvate transaminase | 1.082 | 2.40e-02 | 2.56e-01 |
| psp; phosphoserine phosphatase [EC:3.1.3.3] | 1.347 | 2.41e-02 | 2.56e-01 |
| chiG; putative chitobiose transport system permease protein | 1.492 | 2.42e-02 | 2.57e-01 |
| FLOT; flotillin | 0.631 | 2.47e-02 | 2.61e-01 |
| G6PD, zwf; glucose-6-phosphate 1-dehydrogenase [EC:1.1.1.49 1.1.1.363] | 0.631 | 2.48e-02 | 2.61e-01 |
| araP; arabinosaccharide transport system permease protein | 0.846 | 2.50e-02 | 2.62e-01 |
| betC; choline-sulfatase [EC:3.1.6.6] | 0.737 | 2.50e-02 | 2.62e-01 |
| cebF; cellobiose transport system permease protein | 0.673 | 2.50e-02 | 2.62e-01 |
| dnaI; primosomal protein DnaI | -1.203 | 2.51e-02 | 2.62e-01 |
| traB; conjugal transfer pilus assembly protein TraB | -0.860 | 2.51e-02 | 2.62e-01 |
| K11646; 3-dehydroquinate synthase II [EC:1.4.1.24] | 1.809 | 2.51e-02 | 2.62e-01 |
| pxpB; 5-oxoprolinase (ATP-hydrolysing) subunit B [EC:3.5.2.9] | 0.936 | 2.52e-02 | 2.62e-01 |
| sat, met3; sulfate adenylyltransferase [EC:2.7.7.4] | 1.050 | 2.52e-02 | 2.62e-01 |
| hepC; heparan-sulfate lyase [EC:4.2.2.8] | 1.306 | 2.53e-02 | 2.62e-01 |
| rlmF; 23S rRNA (adenine1618-N6)-methyltransferase [EC:2.1.1.181] | 1.528 | 2.57e-02 | 2.66e-01 |
| pabB; para-aminobenzoate synthetase component I [EC:2.6.1.85] | 0.660 | 2.58e-02 | 2.66e-01 |
| traU; conjugal transfer pilus assembly protein TraU | -0.792 | 2.59e-02 | 2.66e-01 |
| K06951; uncharacterized protein | 1.834 | 2.60e-02 | 2.67e-01 |
| omp31; outer membrane immunogenic protein | -2.575 | 2.60e-02 | 2.67e-01 |
| hydN; electron transport protein HydN | 3.070 | 2.62e-02 | 2.68e-01 |
| coaW; type II pantothenate kinase [EC:2.7.1.33] | 0.742 | 2.64e-02 | 2.69e-01 |
| TC.GBP; general bacterial porin, GBP family | -0.780 | 2.65e-02 | 2.69e-01 |
| cysQ, MET22, BPNT1; 3'(2'), 5'-bisphosphate nucleotidase [EC:3.1.3.7] | 0.533 | 2.65e-02 | 2.69e-01 |
| wcaI; colanic acid biosynthesis glycosyl transferase WcaI | 1.252 | 2.65e-02 | 2.69e-01 |
| E2.6.1.18; beta-alanine--pyruvate transaminase [EC:2.6.1.18] | -2.427 | 2.67e-02 | 2.70e-01 |
| pabA; para-aminobenzoate synthetase component II [EC:2.6.1.85] | -2.054 | 2.68e-02 | 2.70e-01 |
| cpoA; 1,2-diacylglycerol-3-alpha-glucose alpha-1,2-galactosyltransferase [EC:2.4.1.-] | -1.244 | 2.68e-02 | 2.70e-01 |
| mqnK, menK; menaquinone C8-methyltransferase [EC:2.1.1.350] | -0.577 | 2.70e-02 | 2.71e-01 |
| hcaT; MFS transporter, PPP family, 3-phenylpropionic acid transporter | -1.997 | 2.72e-02 | 2.73e-01 |
| pldB; lysophospholipase [EC:3.1.1.5] | -3.344 | 2.73e-02 | 2.73e-01 |
| ysxB; uncharacterized protein | -0.872 | 2.73e-02 | 2.73e-01 |
| rhtC; threonine efflux protein | -1.372 | 2.73e-02 | 2.73e-01 |
| glnB; nitrogen regulatory protein P-II 1 | 0.530 | 2.74e-02 | 2.73e-01 |
| dsrC; dissimilatory sulfite reductase related protein | 1.649 | 2.76e-02 | 2.74e-01 |
| E2.7.13.3; histidine kinase [EC:2.7.13.3] | 1.088 | 2.77e-02 | 2.74e-01 |
| aroKB; shikimate kinase / 3-dehydroquinate synthase [EC:2.7.1.71 4.2.3.4] | -4.785 | 2.78e-02 | 2.75e-01 |
| ynfG; Tat-targeted selenate reductase subunit YnfG | -1.588 | 2.79e-02 | 2.75e-01 |
| tarJ; ribitol-5-phosphate 2-dehydrogenase (NADP+) [EC:1.1.1.405] | -1.162 | 2.81e-02 | 2.77e-01 |
| rimK; ribosomal protein S6--L-glutamate ligase [EC:6.3.2.-] | -1.221 | 2.81e-02 | 2.77e-01 |
| virB4, lvhB4; type IV secretion system protein VirB4 [EC:7.4.2.8] | -0.730 | 2.82e-02 | 2.77e-01 |
| mqnD; 1,4-dihydroxy-6-naphthoate synthase [EC:1.14.-.-] | -0.780 | 2.86e-02 | 2.80e-01 |
| iolT; MFS transporter, SP family, major inositol transporter | -5.540 | 2.87e-02 | 2.81e-01 |
| opuC; osmoprotectant transport system substrate-binding protein | -0.769 | 2.88e-02 | 2.81e-01 |
| COXPD7; peptide chain release factor | -4.890 | 2.88e-02 | 2.81e-01 |
| CBS; cystathionine beta-synthase [EC:4.2.1.22] | -1.840 | 2.89e-02 | 2.81e-01 |
| K09935; uncharacterized protein | 0.618 | 2.90e-02 | 2.81e-01 |
| tamA; translocation and assembly module TamA | 1.289 | 2.90e-02 | 2.81e-01 |
| cbiQ; cobalt/nickel transport system permease protein | -0.748 | 2.91e-02 | 2.81e-01 |
| dsbG; thiol:disulfide interchange protein DsbG | -1.266 | 2.91e-02 | 2.81e-01 |
| frdC; fumarate reductase subunit C | -0.784 | 2.92e-02 | 2.81e-01 |
| treR2, treR; GntR family transcriptional regulator, trehalose operon transcriptional repressor | -0.672 | 2.93e-02 | 2.82e-01 |
| rodZ; cytoskeleton protein RodZ | -0.960 | 2.96e-02 | 2.84e-01 |
| SGSH; N-sulfoglucosamine sulfohydrolase [EC:3.10.1.1] | 1.726 | 2.96e-02 | 2.84e-01 |
| ppk2; polyphosphate kinase [EC:2.7.4.1] | -0.840 | 2.97e-02 | 2.84e-01 |
| soeA; sulfite dehydrogenase (quinone) subunit SoeA [EC:1.8.5.6] | -1.003 | 3.00e-02 | 2.85e-01 |
| cwlK; peptidoglycan LD-endopeptidase CwlK [EC:3.4.-.-] | -1.620 | 3.00e-02 | 2.85e-01 |
| K07074; uncharacterized protein | 0.677 | 3.02e-02 | 2.87e-01 |
| yxdK; two-component system, OmpR family, sensor histidine kinase YxdK [EC:2.7.13.3] | -1.259 | 3.04e-02 | 2.88e-01 |
| tqsA; AI-2 transport protein TqsA | -3.879 | 3.05e-02 | 2.88e-01 |
| catB; chloramphenicol O-acetyltransferase type B [EC:2.3.1.28] | -0.825 | 3.05e-02 | 2.88e-01 |
| K08981; putative membrane protein | -0.625 | 3.07e-02 | 2.89e-01 |
| vioA; dTDP-4-amino-4,6-dideoxy-D-glucose transaminase [EC:2.6.1.33] | -0.548 | 3.07e-02 | 2.89e-01 |
| fadA, fadI; acetyl-CoA acyltransferase [EC:2.3.1.16] | 1.344 | 3.09e-02 | 2.89e-01 |
| rluE; 23S rRNA pseudouridine2457 synthase [EC:5.4.99.20] | -2.706 | 3.09e-02 | 2.90e-01 |
| stbD; antitoxin StbD | 2.445 | 3.10e-02 | 2.90e-01 |
| VIT; vacuolar iron transporter family protein | 0.617 | 3.14e-02 | 2.93e-01 |
| gluP; rhomboid protease GluP [EC:3.4.21.105] | 1.035 | 3.15e-02 | 2.94e-01 |
| speD, AMD1; S-adenosylmethionine decarboxylase [EC:4.1.1.50] | 1.149 | 3.16e-02 | 2.94e-01 |
| accD3; acyl-CoA carboxylase subunit beta [EC:6.4.1.-] | 0.604 | 3.16e-02 | 2.94e-01 |
| otnK; 3-dehydrotetronate 4-kinase [EC:2.7.1.217] | 0.796 | 3.18e-02 | 2.94e-01 |
| ulaD, sgaH, sgbH; 3-dehydro-L-gulonate-6-phosphate decarboxylase [EC:4.1.1.85] | 0.677 | 3.19e-02 | 2.94e-01 |
| pfpI; protease I [EC:3.5.1.124] | 0.759 | 3.21e-02 | 2.94e-01 |
| rlmJ; 23S rRNA (adenine2030-N6)-methyltransferase [EC:2.1.1.266] | 2.377 | 3.21e-02 | 2.94e-01 |
| hofP; pilus assembly protein HofP | -3.675 | 3.21e-02 | 2.94e-01 |
| cobC, phpB; alpha-ribazole phosphatase [EC:3.1.3.73] | 0.587 | 3.22e-02 | 2.95e-01 |
| ywlE; protein arginine phosphatase [EC:3.9.1.2] | 1.396 | 3.23e-02 | 2.95e-01 |
| aapP, bztD; general L-amino acid transport system ATP-binding protein [EC:7.4.2.1] | 1.230 | 3.25e-02 | 2.97e-01 |
| GSR, gor; glutathione reductase (NADPH) [EC:1.8.1.7] | -1.158 | 3.28e-02 | 2.99e-01 |
| ppc; phosphoenolpyruvate carboxylase [EC:4.1.1.31] | -1.491 | 3.29e-02 | 2.99e-01 |
| gmuG; mannan endo-1,4-beta-mannosidase [EC:3.2.1.78] | 0.538 | 3.34e-02 | 3.01e-01 |
| waaO, rfaI; UDP-glucose:(glucosyl)LPS alpha-1,3-glucosyltransferase [EC:2.4.1.-] | -0.781 | 3.34e-02 | 3.01e-01 |
| hprA; glycerate dehydrogenase [EC:1.1.1.29] | 0.524 | 3.34e-02 | 3.01e-01 |
| higB-1; toxin HigB-1 | 0.506 | 3.35e-02 | 3.01e-01 |
| celB, chbC; cellobiose PTS system EIIC component | -0.594 | 3.35e-02 | 3.01e-01 |
| tesB; acyl-CoA thioesterase II [EC:3.1.2.-] | -4.522 | 3.41e-02 | 3.06e-01 |
| rne; ribonuclease E [EC:3.1.26.12] | -0.597 | 3.43e-02 | 3.07e-01 |
| ecfTA; energy-coupling factor transport system permease/ATP-binding protein [EC:3.6.3.-] | -4.427 | 3.44e-02 | 3.07e-01 |
| K07219; putative molybdopterin biosynthesis protein | -1.925 | 3.45e-02 | 3.07e-01 |
| mdtG; MFS transporter, DHA1 family, multidrug resistance protein | -0.689 | 3.46e-02 | 3.08e-01 |
| ABC.GGU.S, chvE; putative multiple sugar transport system substrate-binding protein | 0.789 | 3.49e-02 | 3.10e-01 |
| K14645; serine protease [EC:3.4.21.-] | 0.698 | 3.49e-02 | 3.10e-01 |
| UMF1; MFS transporter, UMF1 family | -0.761 | 3.51e-02 | 3.10e-01 |
| malG; maltose/maltodextrin transport system permease protein | -4.811 | 3.54e-02 | 3.12e-01 |
| cpg; glutamate carboxypeptidase [EC:3.4.17.11] | -0.545 | 3.55e-02 | 3.12e-01 |
| caiC; carnitine-CoA ligase [EC:6.2.1.48] | 1.608 | 3.59e-02 | 3.15e-01 |
| ccmH; cytochrome c-type biogenesis protein CcmH | -0.783 | 3.65e-02 | 3.19e-01 |
| yaeR; glyoxylase I family protein | 0.690 | 3.67e-02 | 3.19e-01 |
| nlpI; lipoprotein NlpI | 2.117 | 3.67e-02 | 3.19e-01 |
| dgkA; undecaprenol kinase [EC:2.7.1.66] | -2.511 | 3.68e-02 | 3.19e-01 |
| E1.1.1.30, bdh; 3-hydroxybutyrate dehydrogenase [EC:1.1.1.30] | -1.558 | 3.68e-02 | 3.19e-01 |
| mdcH; malonate decarboxylase epsilon subunit [EC:2.3.1.39] | -1.477 | 3.69e-02 | 3.19e-01 |
| tagG; teichoic acid transport system permease protein | -0.791 | 3.69e-02 | 3.20e-01 |
| tetB; MFS transporter, DHA2 family, metal-tetracycline-proton antiporter | -5.292 | 3.72e-02 | 3.21e-01 |
| norG; GntR family transcriptional regulator, regulator for abcA and norABC | -1.718 | 3.74e-02 | 3.22e-01 |
| tsr; methyl-accepting chemotaxis protein I, serine sensor receptor | 1.341 | 3.75e-02 | 3.22e-01 |
| pseB; UDP-N-acetylglucosamine 4,6-dehydratase [EC:4.2.1.115] | 0.553 | 3.75e-02 | 3.22e-01 |
| pseG; UDP-2,4-diacetamido-2,4,6-trideoxy-beta-L-altropyranose hydrolase [EC:3.6.1.57] | 0.754 | 3.76e-02 | 3.22e-01 |
| ubiB, aarF; ubiquinone biosynthesis protein | -1.121 | 3.78e-02 | 3.23e-01 |
| dgaB; D-glucosaminate PTS system EIIB component [EC:2.7.1.203] | -1.038 | 3.78e-02 | 3.23e-01 |
| tctB; putative tricarboxylic transport membrane protein | -0.884 | 3.80e-02 | 3.24e-01 |
| echA; ech hydrogenase subunit A | -0.795 | 3.83e-02 | 3.26e-01 |
| mnhB, mrpB; multicomponent Na+:H+ antiporter subunit B | 0.805 | 3.83e-02 | 3.26e-01 |
| mobA; molybdenum cofactor guanylyltransferase [EC:2.7.7.77] | -1.314 | 3.86e-02 | 3.28e-01 |
| spoVFB; dipicolinate synthase subunit B | 0.640 | 3.87e-02 | 3.29e-01 |
| tarI; D-ribitol-5-phosphate cytidylyltransferase [EC:2.7.7.40] | -1.005 | 3.88e-02 | 3.29e-01 |
| glpB; glycerol-3-phosphate dehydrogenase subunit B [EC:1.1.5.3] | -0.504 | 3.92e-02 | 3.31e-01 |
| treC; trehalose-6-phosphate hydrolase [EC:3.2.1.93] | 0.632 | 3.95e-02 | 3.33e-01 |
| K09131; uncharacterized protein | -5.233 | 3.96e-02 | 3.33e-01 |
| gshA; glutamate--cysteine ligase [EC:6.3.2.2] | -0.846 | 3.99e-02 | 3.35e-01 |
| priB; primosomal replication protein N | -1.646 | 4.02e-02 | 3.36e-01 |
| ptrB; oligopeptidase B [EC:3.4.21.83] | -2.616 | 4.02e-02 | 3.36e-01 |
| rpoE; DNA-directed RNA polymerase subunit delta | -0.627 | 4.02e-02 | 3.36e-01 |
| prpR; transcriptional regulator, propionate catabolism operon regulatory protein | 0.766 | 4.04e-02 | 3.37e-01 |
| modC; molybdate transport system ATP-binding protein [EC:7.3.2.5] | -0.511 | 4.11e-02 | 3.42e-01 |
| shc; squalene-hopene/tetraprenyl-beta-curcumene cyclase [EC:5.4.99.17 4.2.1.129] | -2.694 | 4.16e-02 | 3.44e-01 |
| prmB; ribosomal protein L3 glutamine methyltransferase [EC:2.1.1.298] | -1.220 | 4.22e-02 | 3.48e-01 |
| modE; molybdate transport system regulatory protein | -1.034 | 4.23e-02 | 3.48e-01 |
| bamB; outer membrane protein assembly factor BamB | -0.941 | 4.23e-02 | 3.48e-01 |
| cysA; sulfate/thiosulfate transport system ATP-binding protein [EC:7.3.2.3] | 1.177 | 4.24e-02 | 3.48e-01 |
| K07177; Lon-like protease | -1.578 | 4.24e-02 | 3.48e-01 |
| K16149; 1,4-alpha-glucan branching enzyme [EC:2.4.1.18] | 1.102 | 4.25e-02 | 3.48e-01 |
| GALT29A; beta-1,6-galactosyltransferase [EC:2.4.1.-] | 1.335 | 4.30e-02 | 3.52e-01 |
| fliS; flagellar protein FliS | 0.778 | 4.30e-02 | 3.52e-01 |
| rlmA1; 23S rRNA (guanine745-N1)-methyltransferase [EC:2.1.1.187] | -1.067 | 4.33e-02 | 3.52e-01 |
| E1.1.1.65; pyridoxine 4-dehydrogenase [EC:1.1.1.65] | -3.030 | 4.33e-02 | 3.52e-01 |
| asrC; anaerobic sulfite reductase subunit C | 0.608 | 4.33e-02 | 3.52e-01 |
| mleA, mleS; malolactic enzyme [EC:4.1.1.101] | 0.528 | 4.40e-02 | 3.57e-01 |
| rfbCD, rmlCD; dTDP-4-dehydrorhamnose 3,5-epimerase/reductase [EC:5.1.3.13 1.1.1.133] | -4.553 | 4.40e-02 | 3.57e-01 |
| lmrB; MFS transporter, DHA2 family, lincomycin resistance protein | -1.076 | 4.43e-02 | 3.59e-01 |
| umuD; DNA polymerase V [EC:3.4.21.-] | -1.332 | 4.47e-02 | 3.61e-01 |
| tctC; putative tricarboxylic transport membrane protein | -1.212 | 4.49e-02 | 3.61e-01 |
| manXa; mannose PTS system EIIA component [EC:2.7.1.191] | -1.390 | 4.49e-02 | 3.61e-01 |
| tpdA; cysteinylglycine-S-conjugate dipeptidase [EC:3.4.13.23] | -5.019 | 4.49e-02 | 3.61e-01 |
| AARSD1, ALAX; misacylated tRNA(Ala) deacylase [EC:3.1.1.-] | 0.692 | 4.51e-02 | 3.61e-01 |
| qor, CRYZ; NADPH:quinone reductase [EC:1.6.5.5] | -1.161 | 4.52e-02 | 3.61e-01 |
| treS; maltose alpha-D-glucosyltransferase / alpha-amylase [EC:5.4.99.16 3.2.1.1] | 0.656 | 4.53e-02 | 3.61e-01 |
| sbnB; N-[(2S)-2-amino-2-carboxyethyl]-L-glutamate dehydrogenase [EC:1.5.1.51] | 1.230 | 4.55e-02 | 3.62e-01 |
| flhB; flagellar biosynthetic protein FlhB | 0.577 | 4.57e-02 | 3.63e-01 |
| ganP; arabinogalactan oligomer / maltooligosaccharide transport system permease protein | 0.576 | 4.57e-02 | 3.63e-01 |
| ABC.X4.S; putative ABC transport system substrate-binding protein | -0.505 | 4.58e-02 | 3.63e-01 |
| gerKA; spore germination protein KA | 0.791 | 4.59e-02 | 3.63e-01 |
| bamC; outer membrane protein assembly factor BamC | -0.718 | 4.64e-02 | 3.67e-01 |
| cooA; CRP/FNR family transcriptional regulator, carbon monoxide oxidation system transcription regulator | -1.062 | 4.64e-02 | 3.67e-01 |
| tarL; CDP-ribitol ribitolphosphotransferase / teichoic acid ribitol-phosphate polymerase [EC:2.7.8.14 2.7.8.47] | 1.373 | 4.69e-02 | 3.70e-01 |
| pelG; polysaccharide biosynthesis protein PelG | 1.419 | 4.73e-02 | 3.72e-01 |
| yitJ; methionine synthase / methylenetetrahydrofolate reductase(NADPH) [EC:2.1.1.13 1.5.1.20] | -1.413 | 4.75e-02 | 3.74e-01 |
| cbiK; sirohydrochlorin cobaltochelatase [EC:4.99.1.3] | 0.579 | 4.80e-02 | 3.75e-01 |
| alpA; prophage regulatory protein | -0.813 | 4.80e-02 | 3.75e-01 |
| lytH; peptidoglycan LD-endopeptidase LytH [EC:3.4.-.-] | 0.602 | 4.81e-02 | 3.75e-01 |
| fruB; fructose PTS system EIIA component [EC:2.7.1.202] | -2.042 | 4.83e-02 | 3.76e-01 |
| PSMC1, RPT2; 26S proteasome regulatory subunit T2 | -2.043 | 4.85e-02 | 3.77e-01 |
| codY; transcriptional pleiotropic repressor | 0.560 | 4.85e-02 | 3.77e-01 |
| E3.2.1.58; glucan 1,3-beta-glucosidase [EC:3.2.1.58] | 0.619 | 4.87e-02 | 3.77e-01 |
| norB; nitric oxide reductase subunit B [EC:1.7.2.5] | -0.755 | 4.90e-02 | 3.79e-01 |
| casE, cse3; CRISPR system Cascade subunit CasE | -3.641 | 4.93e-02 | 3.81e-01 |
| K07010; putative glutamine amidotransferase | -0.583 | 4.97e-02 | 3.83e-01 |
| fmnP, ribU; riboflavin transporter | -0.562 | 4.97e-02 | 3.83e-01 |
| PGC-1α-Ex after vs. Wt-Ex after | | | |
| rpoE; RNA polymerase sigma-70 factor, ECF subfamily | 0.518 | 1.93e-38 | 1.33e-34\* |
| cimA; (R)-citramalate synthase [EC:2.3.1.182] | 0.833 | 4.06e-28 | 1.40e-24\* |
| ARSA; arylsulfatase A [EC:3.1.6.8] | 0.922 | 1.09e-20 | 2.50e-17\* |
| rluA; tRNA pseudouridine32 synthase / 23S rRNA pseudouridine746 synthase [EC:5.4.99.28 5.4.99.29] | 1.268 | 8.67e-19 | 1.49e-15\* |
| pyrD; dihydroorotate dehydrogenase (fumarate) [EC:1.3.98.1] | 1.181 | 3.79e-16 | 5.22e-13\* |
| afr; 1,5-anhydro-D-fructose reductase (1,5-anhydro-D-mannitol-forming) [EC:1.1.1.292] | 1.378 | 5.09e-16 | 5.84e-13\* |
| yfiQ; acetyltransferase | 1.279 | 6.20e-16 | 6.09e-13\* |
| trcR; two-component system, OmpR family, response regulator TrcR | 1.191 | 9.72e-16 | 8.36e-13\* |
| E3.1.3.1, phoA, phoB; alkaline phosphatase [EC:3.1.3.1] | 0.613 | 1.34e-15 | 1.02e-12\* |
| TC.APA; basic amino acid/polyamine antiporter, APA family | 0.726 | 1.01e-14 | 6.95e-12\* |
| tldD; TldD protein | 1.092 | 1.60e-14 | 1.00e-11\* |
| emrA; membrane fusion protein, multidrug efflux system | 1.154 | 2.66e-14 | 1.53e-11\* |
| lapE; outer membrane protein, adhesin transport system | 1.492 | 5.54e-14 | 2.93e-11\* |
| amn; AMP nucleosidase [EC:3.2.2.4] | 1.084 | 6.10e-14 | 3.00e-11\* |
| cbpA; curved DNA-binding protein | 0.725 | 7.90e-14 | 3.62e-11\* |
| E3.1.11.5; exodeoxyribonuclease V [EC:3.1.11.5] | 1.001 | 4.16e-13 | 1.79e-10\* |
| K13652; AraC family transcriptional regulator | 1.710 | 1.46e-12 | 5.70e-10\* |
| ohrR; MarR family transcriptional regulator, organic hydroperoxide resistance regulator | 0.975 | 1.49e-12 | 5.70e-10\* |
| K08961; chondroitin-sulfate-ABC endolyase/exolyase [EC:4.2.2.20 4.2.2.21] | 1.346 | 2.69e-12 | 9.74e-10\* |
| cysN; sulfate adenylyltransferase subunit 1 [EC:2.7.7.4] | 0.982 | 6.63e-12 | 2.28e-09\* |
| gmuG; mannan endo-1,4-beta-mannosidase [EC:3.2.1.78] | 0.898 | 3.01e-11 | 9.82e-09\* |
| thiN, TPK1, THI80; thiamine pyrophosphokinase [EC:2.7.6.2] | 1.162 | 3.14e-11 | 9.82e-09\* |
| K00666; fatty-acyl-CoA synthase [EC:6.2.1.-] | 1.034 | 3.69e-11 | 1.10e-08\* |
| AARSD1, ALAX; misacylated tRNA(Ala) deacylase [EC:3.1.1.-] | 1.289 | 6.62e-11 | 1.90e-08\* |
| ugl; unsaturated chondroitin disaccharide hydrolase [EC:3.2.1.180] | 0.724 | 7.58e-11 | 2.09e-08\* |
| clpA; ATP-dependent Clp protease ATP-binding subunit ClpA | 0.585 | 8.16e-11 | 2.16e-08\* |
| E3.2.1.4; endoglucanase [EC:3.2.1.4] | 1.113 | 2.19e-10 | 5.43e-08\* |
| cysD; sulfate adenylyltransferase subunit 2 [EC:2.7.7.4] | 0.875 | 2.21e-10 | 5.43e-08\* |
| dgkA, DGK; diacylglycerol kinase (ATP) [EC:2.7.1.107] | 1.305 | 5.79e-10 | 1.37e-07\* |
| cbe, mbe; cellobiose epimerase [EC:5.1.3.11] | 0.830 | 1.07e-09 | 2.45e-07\* |
| abgT; aminobenzoyl-glutamate transport protein | 1.114 | 1.33e-09 | 2.95e-07\* |
| ubiG; 2-polyprenyl-6-hydroxyphenyl methylase / 3-demethylubiquinone-9 3-methyltransferase [EC:2.1.1.222 2.1.1.64] | 1.088 | 1.42e-09 | 3.05e-07\* |
| emrB; MFS transporter, DHA2 family, multidrug resistance protein | 1.382 | 1.62e-09 | 3.38e-07\* |
| coaW; type II pantothenate kinase [EC:2.7.1.33] | 1.398 | 2.59e-09 | 5.24e-07\* |
| cah; cephalosporin-C deacetylase [EC:3.1.1.41] | 1.230 | 4.23e-09 | 8.31e-07\* |
| bioC; malonyl-CoA O-methyltransferase [EC:2.1.1.197] | 1.314 | 4.68e-09 | 8.94e-07\* |
| PGD, gnd, gntZ; 6-phosphogluconate dehydrogenase [EC:1.1.1.44 1.1.1.343] | 0.943 | 6.66e-09 | 1.24e-06\* |
| clpS; ATP-dependent Clp protease adaptor protein ClpS | 1.227 | 1.19e-08 | 2.15e-06\* |
| HGSNAT; heparan-alpha-glucosaminide N-acetyltransferase [EC:2.3.1.78] | 1.723 | 1.38e-08 | 2.43e-06\* |
| cutC; copper homeostasis protein | 0.954 | 1.45e-08 | 2.49e-06\* |
| rclC; reactive chlorine resistance protein C | 1.635 | 1.51e-08 | 2.53e-06\* |
| fepA, pfeA, iroN, pirA; ferric enterobactin receptor | 1.357 | 2.05e-08 | 3.28e-06\* |
| pmbA; PmbA protein | 1.154 | 2.12e-08 | 3.31e-06\* |
| rclA; probable pyridine nucleotide-disulfide oxidoreductase | 0.886 | 2.57e-08 | 3.93e-06\* |
| E3.2.1.14; chitinase [EC:3.2.1.14] | 1.508 | 3.29e-08 | 4.92e-06\* |
| phoH2; PhoH-like ATPase | 0.963 | 3.75e-08 | 5.38e-06\* |
| tolC; outer membrane protein | 0.665 | 4.44e-08 | 6.23e-06\* |
| PGLS, pgl, devB; 6-phosphogluconolactonase [EC:3.1.1.31] | 1.338 | 5.23e-08 | 7.20e-06\* |
| E3.1.3.15B; histidinol-phosphatase (PHP family) [EC:3.1.3.15] | 0.525 | 6.00e-08 | 8.09e-06\* |
| glpT; MFS transporter, OPA family, glycerol-3-phosphate transporter | -0.561 | 7.58e-08 | 1.00e-05\* |
| dexA; dextranase [EC:3.2.1.11] | 0.897 | 1.08e-07 | 1.40e-05\* |
| cobC, phpB; alpha-ribazole phosphatase [EC:3.1.3.73] | 1.360 | 1.61e-07 | 1.98e-05\* |
| anmK; anhydro-N-acetylmuramic acid kinase [EC:2.7.1.170] | 1.242 | 2.22e-07 | 2.63e-05\* |
| MAN; mannan endo-1,4-beta-mannosidase [EC:3.2.1.78] | 1.180 | 2.43e-07 | 2.83e-05\* |
| paaI; acyl-CoA thioesterase [EC:3.1.2.-] | 1.124 | 2.54e-07 | 2.91e-05\* |
| pabB; para-aminobenzoate synthetase component I [EC:2.6.1.85] | 0.785 | 2.70e-07 | 3.05e-05\* |
| cysC; adenylylsulfate kinase [EC:2.7.1.25] | 1.069 | 3.00e-07 | 3.33e-05\* |
| E3.5.2.10; creatinine amidohydrolase [EC:3.5.2.10] | 1.102 | 3.33e-07 | 3.64e-05\* |
| K07017; uncharacterized protein | 1.469 | 3.41e-07 | 3.67e-05\* |
| iolW; scyllo-inositol 2-dehydrogenase (NADP+) [EC:1.1.1.371] | 1.138 | 3.71e-07 | 3.90e-05\* |
| pyrR; pyrimidine operon attenuation protein / uracil phosphoribosyltransferase [EC:2.4.2.9] | -1.269 | 3.74e-07 | 3.90e-05\* |
| blpB; membrane fusion protein, peptide pheromone/bacteriocin exporter | 1.435 | 4.42e-07 | 4.54e-05\* |
| K09992; uncharacterized protein | 1.282 | 5.12e-07 | 5.18e-05\* |
| djlA; DnaJ like chaperone protein | 1.457 | 5.34e-07 | 5.32e-05\* |
| IGHMBP2; ATP-dependent RNA/DNA helicase IGHMBP2 [EC:3.6.4.12 3.6.4.13] | 0.664 | 6.39e-07 | 6.19e-05\* |
| K07482; transposase, IS30 family | -0.669 | 8.86e-07 | 8.47e-05\* |
| hdhA; 7-alpha-hydroxysteroid dehydrogenase [EC:1.1.1.159] | -0.865 | 1.05e-06 | 9.90e-05\* |
| agaR; DeoR family transcriptional regulator, aga operon transcriptional repressor | -0.850 | 1.82e-06 | 1.65e-04\* |
| mntH; manganese transport protein | 0.736 | 1.82e-06 | 1.65e-04\* |
| evgS, bvgS; two-component system, NarL family, sensor histidine kinase EvgS [EC:2.7.13.3] | 0.521 | 2.11e-06 | 1.86e-04\* |
| licD; lipopolysaccharide cholinephosphotransferase [EC:2.7.8.-] | 0.523 | 2.29e-06 | 1.98e-04\* |
| ltaE; threonine aldolase [EC:4.1.2.48] | 0.617 | 2.73e-06 | 2.32e-04\* |
| DNA2; DNA replication ATP-dependent helicase Dna2 [EC:3.6.4.12] | -4.352 | 3.26e-06 | 2.74e-04\* |
| lacI, galR; LacI family transcriptional regulator | 0.605 | 4.14e-06 | 3.39e-04\* |
| susA; neopullulanase [EC:3.2.1.135] | 0.667 | 4.53e-06 | 3.62e-04\* |
| E5.2.1.8; peptidylprolyl isomerase [EC:5.2.1.8] | 1.270 | 4.67e-06 | 3.69e-04\* |
| qseC; two-component system, OmpR family, sensor histidine kinase QseC [EC:2.7.13.3] | 1.257 | 4.87e-06 | 3.81e-04\* |
| mexJ; membrane fusion protein, multidrug efflux system | 0.848 | 5.06e-06 | 3.91e-04\* |
| oadG; oxaloacetate decarboxylase (Na+ extruding) subunit gamma | 1.187 | 6.63e-06 | 5.01e-04\* |
| nagK; fumarylpyruvate hydrolase [EC:3.7.1.20] | 1.210 | 6.97e-06 | 5.21e-04\* |
| queH; epoxyqueuosine reductase [EC:1.17.99.6] | 0.526 | 7.31e-06 | 5.41e-04\* |
| AUR1; inositol phosphorylceramide synthase catalytic subunit [EC:2.7.1.227] | 1.234 | 8.63e-06 | 6.32e-04\* |
| K03710; GntR family transcriptional regulator | -0.849 | 8.96e-06 | 6.49e-04\* |
| K02477; two-component system, LytTR family, response regulator | 0.781 | 9.41e-06 | 6.74e-04\* |
| yihX; glucose-1-phosphatase [EC:3.1.3.10] | 0.549 | 1.07e-05 | 7.51e-04\* |
| plc; 1-phosphatidylinositol phosphodiesterase [EC:4.6.1.13] | 1.664 | 1.11e-05 | 7.71e-04\* |
| mapA; maltose phosphorylase [EC:2.4.1.8] | 0.735 | 1.19e-05 | 8.19e-04\* |
| patA; aminotransferase [EC:2.6.1.-] | -1.795 | 1.44e-05 | 9.81e-04\* |
| G6PD, zwf; glucose-6-phosphate 1-dehydrogenase [EC:1.1.1.49 1.1.1.363] | 0.967 | 1.50e-05 | 1.01e-03\* |
| dld; D-lactate dehydrogenase | 1.015 | 1.87e-05 | 1.24e-03\* |
| K09973; uncharacterized protein | 1.202 | 1.91e-05 | 1.25e-03\* |
| tyrA; chorismate mutase / prephenate dehydrogenase [EC:5.4.99.5 1.3.1.12] | 1.136 | 2.23e-05 | 1.45e-03\* |
| buk; butyrate kinase [EC:2.7.2.7] | 0.552 | 2.25e-05 | 1.45e-03\* |
| zapA; cell division protein ZapA | 0.658 | 2.45e-05 | 1.56e-03\* |
| dacB; serine-type D-Ala-D-Ala carboxypeptidase/endopeptidase (penicillin-binding protein 4) [EC:3.4.16.4 3.4.21.-] | 0.828 | 2.53e-05 | 1.60e-03\* |
| dptH; DNA phosphorothioation-dependent restriction protein DptH | 1.252 | 2.56e-05 | 1.60e-03\* |
| hdeD; membrane protein HdeD | 1.305 | 2.61e-05 | 1.61e-03\* |
| K07089; uncharacterized protein | -0.625 | 3.41e-05 | 2.08e-03\* |
| aat; leucyl/phenylalanyl-tRNA---protein transferase [EC:2.3.2.6] | 0.608 | 3.48e-05 | 2.09e-03\* |
| K09924; uncharacterized protein | 1.651 | 3.49e-05 | 2.09e-03\* |
| comFA; competence protein ComFA | -2.261 | 4.27e-05 | 2.49e-03\* |
| vanX; zinc D-Ala-D-Ala dipeptidase [EC:3.4.13.22] | 0.690 | 4.39e-05 | 2.54e-03\* |
| potD; spermidine/putrescine transport system substrate-binding protein | 0.636 | 4.71e-05 | 2.68e-03\* |
| E1.1.1.90; aryl-alcohol dehydrogenase [EC:1.1.1.90] | -2.215 | 4.93e-05 | 2.78e-03\* |
| fmtA; teichoic acid D-alanine hydrolase [EC:3.1.1.103] | -2.355 | 5.11e-05 | 2.86e-03\* |
| ABC.X2.A; putative ABC transport system ATP-binding protein | 1.308 | 5.18e-05 | 2.87e-03\* |
| wzxC; lipopolysaccharide exporter | 1.475 | 5.99e-05 | 3.30e-03\* |
| TC.BAT1; bacterial/archaeal transporter family protein | 0.525 | 6.65e-05 | 3.63e-03\* |
| SIAE; sialate O-acetylesterase [EC:3.1.1.53] | 0.505 | 8.56e-05 | 4.50e-03\* |
| TRIP4; activating signal cointegrator 1 | 1.096 | 1.03e-04 | 5.36e-03\* |
| mntP; manganese efflux pump family protein | 0.648 | 1.08e-04 | 5.55e-03\* |
| melB; melibiose permease | 0.843 | 1.09e-04 | 5.55e-03\* |
| E2.7.3.13; glutamine kinase [EC:2.7.3.13] | 1.833 | 1.24e-04 | 6.29e-03\* |
| ccdA; cytochrome c-type biogenesis protein | -1.340 | 1.51e-04 | 7.50e-03\* |
| wcaE; putative colanic acid biosynthesis glycosyltransferase [EC:2.4.-.-] | 1.594 | 1.51e-04 | 7.50e-03\* |
| MAN2C1; alpha-mannosidase [EC:3.2.1.24] | 0.765 | 1.63e-04 | 7.93e-03\* |
| troR; DtxR family transcriptional regulator, Mn-dependent transcriptional regulator | -0.534 | 1.65e-04 | 7.93e-03\* |
| pqqE; PqqA peptide cyclase [EC:1.21.98.4] | 1.607 | 1.73e-04 | 8.20e-03\* |
| cslA; chondroitin AC lyase [EC:4.2.2.5] | 1.294 | 1.90e-04 | 8.87e-03\* |
| K17202, eryG; erythritol transport system substrate-binding protein | -2.455 | 2.06e-04 | 9.50e-03\* |
| patA, rscA, lmrC, satA; ATP-binding cassette, subfamily B, multidrug efflux pump | -1.440 | 2.21e-04 | 9.99e-03\* |
| cobL; precorrin-6Y C5,15-methyltransferase (decarboxylating) [EC:2.1.1.132] | 0.852 | 2.47e-04 | 1.11e-02\* |
| K07076; uncharacterized protein | 0.703 | 2.54e-04 | 1.13e-02\* |
| RDH12; retinol dehydrogenase 12 [EC:1.1.1.300] | 1.176 | 2.56e-04 | 1.13e-02\* |
| aroD; 3-dehydroquinate dehydratase I [EC:4.2.1.10] | -1.140 | 2.59e-04 | 1.13e-02\* |
| pgl; 6-phosphogluconolactonase [EC:3.1.1.31] | 0.826 | 2.61e-04 | 1.13e-02\* |
| purQ; phosphoribosylformylglycinamidine synthase subunit PurQ / glutaminase [EC:6.3.5.3 3.5.1.2] | -1.881 | 2.79e-04 | 1.20e-02\* |
| ldcA; muramoyltetrapeptide carboxypeptidase [EC:3.4.17.13] | 0.514 | 3.28e-04 | 1.39e-02\* |
| cshB; ATP-dependent RNA helicase CshB [EC:3.6.4.13] | -0.888 | 3.49e-04 | 1.47e-02\* |
| pseG; UDP-2,4-diacetamido-2,4,6-trideoxy-beta-L-altropyranose hydrolase [EC:3.6.1.57] | 1.374 | 3.54e-04 | 1.49e-02\* |
| ydhQ; GntR family transcriptional regulator | -1.034 | 3.61e-04 | 1.50e-02\* |
| mleA, mleS; malolactic enzyme [EC:4.1.1.101] | -0.973 | 3.63e-04 | 1.50e-02\* |
| bcrC; undecaprenyl-diphosphatase [EC:3.6.1.27] | 0.541 | 3.66e-04 | 1.50e-02\* |
| blpA, lagD; ATP-binding cassette, subfamily C, bacteriocin exporter | 0.899 | 3.67e-04 | 1.50e-02\* |
| upp; UTP pyrophosphatase [EC:3.6.1.-] | 0.607 | 3.86e-04 | 1.55e-02\* |
| raxA; membrane fusion protein | 1.800 | 4.16e-04 | 1.65e-02\* |
| pbuX; xanthine permease | -1.053 | 4.21e-04 | 1.66e-02\* |
| folB; 7,8-dihydroneopterin aldolase/epimerase/oxygenase [EC:4.1.2.25 5.1.99.8 1.13.11.81] | 0.510 | 4.30e-04 | 1.69e-02\* |
| acm; lysozyme | 0.555 | 4.51e-04 | 1.76e-02\* |
| comC; leader peptidase (prepilin peptidase) / N-methyltransferase [EC:3.4.23.43 2.1.1.-] | -3.731 | 4.60e-04 | 1.79e-02\* |
| panE, apbA; 2-dehydropantoate 2-reductase [EC:1.1.1.169] | 0.522 | 4.68e-04 | 1.81e-02\* |
| NQO1; NAD(P)H dehydrogenase (quinone) [EC:1.6.5.2] | -1.166 | 4.70e-04 | 1.81e-02\* |
| nreC; two-component system, NarL family, response regulator NreC | 0.596 | 5.00e-04 | 1.90e-02\* |
| dnaI; primosomal protein DnaI | -1.259 | 5.10e-04 | 1.93e-02\* |
| cysA; sulfate/thiosulfate transport system ATP-binding protein [EC:7.3.2.3] | 1.547 | 5.27e-04 | 1.98e-02\* |
| speG, SAT; diamine N-acetyltransferase [EC:2.3.1.57] | 0.645 | 5.42e-04 | 2.03e-02\* |
| fur, zur, furB; Fur family transcriptional regulator, ferric uptake regulator | 0.535 | 5.71e-04 | 2.12e-02\* |
| aac6-I, aacA7; aminoglycoside 6'-N-acetyltransferase I [EC:2.3.1.82] | 1.490 | 5.89e-04 | 2.18e-02\* |
| cbiD; cobalt-precorrin-5B (C1)-methyltransferase [EC:2.1.1.195] | 0.661 | 6.03e-04 | 2.21e-02\* |
| K09927; uncharacterized protein | -1.029 | 6.05e-04 | 2.21e-02\* |
| pseF; pseudaminic acid cytidylyltransferase [EC:2.7.7.81] | 0.921 | 6.13e-04 | 2.23e-02\* |
| yebQ; MFS transporter, DHA2 family, multidrug resistance protein | 0.515 | 6.80e-04 | 2.45e-02\* |
| ybaZ; methylated-DNA-protein-cysteine methyltransferase related protein | 0.700 | 7.05e-04 | 2.53e-02\* |
| cydC; ATP-binding cassette, subfamily C, bacterial CydC | -1.011 | 7.20e-04 | 2.57e-02\* |
| msrC; L-methionine (R)-S-oxide reductase [EC:1.8.4.14] | 0.600 | 7.89e-04 | 2.77e-02\* |
| E1.7.1.7, guaC; GMP reductase [EC:1.7.1.7] | -0.565 | 8.31e-04 | 2.88e-02\* |
| K06994; putative drug exporter of the RND superfamily | -1.778 | 8.34e-04 | 2.88e-02\* |
| ureE; urease accessory protein | -0.617 | 9.10e-04 | 3.09e-02\* |
| tesA; acyl-CoA thioesterase I [EC:3.1.2.- 3.1.2.2 3.1.1.2 3.1.1.5] | -1.835 | 9.62e-04 | 3.23e-02\* |
| E3.6.1.22, NUDT12, nudC; NAD+ diphosphatase [EC:3.6.1.22] | 0.769 | 1.00e-03 | 3.32e-02\* |
| TC.PST; polysaccharide transporter, PST family | -1.205 | 1.03e-03 | 3.39e-02\* |
| cobI-cbiL; precorrin-2/cobalt-factor-2 C20-methyltransferase [EC:2.1.1.130 2.1.1.151] | 0.572 | 1.06e-03 | 3.43e-02\* |
| SMARCAL1, HARP; SWI/SNF-related matrix-associated actin-dependent regulator of chromatin subfamily A-like protein 1 [EC:3.6.4.12] | -0.967 | 1.14e-03 | 3.67e-02\* |
| dmsC; dimethyl sulfoxide reductase membrane subunit | -1.181 | 1.25e-03 | 3.91e-02\* |
| ABC.X2.P; putative ABC transport system permease protein | 1.100 | 1.25e-03 | 3.91e-02\* |
| plcR; HTH-type transcriptional regulator, pleiotropic regulator of extracellular virulence genes | -3.159 | 1.32e-03 | 4.09e-02\* |
| PCYT1; choline-phosphate cytidylyltransferase [EC:2.7.7.15] | -0.849 | 1.35e-03 | 4.15e-02\* |
| atl; bifunctional autolysin [EC:3.5.1.28 3.2.1.96] | -1.999 | 1.41e-03 | 4.31e-02\* |
| ugtP; processive 1,2-diacylglycerol beta-glucosyltransferase [EC:2.4.1.315] | -1.698 | 1.42e-03 | 4.32e-02\* |
| fnr; ferredoxin/flavodoxin---NADP+ reductase [EC:1.18.1.2 1.19.1.1] | -1.514 | 1.43e-03 | 4.33e-02\* |
| mdlC; benzoylformate decarboxylase [EC:4.1.1.7] | -3.506 | 1.46e-03 | 4.40e-02\* |
| E4.1.1.17, ODC1, speC, speF; ornithine decarboxylase [EC:4.1.1.17] | -0.504 | 1.58e-03 | 4.71e-02\* |
| lgaB; L-galactono-1,5-lactonase [EC:3.1.1.-] | -0.731 | 1.60e-03 | 4.72e-02\* |
| lytS; two-component system, LytTR family, sensor histidine kinase LytS [EC:2.7.13.3] | -1.275 | 1.60e-03 | 4.72e-02\* |
| purR; purine operon repressor | -0.812 | 1.73e-03 | 5.07e-02 |
| wbpP; UDP-N-acetylglucosamine 4-epimerase [EC:5.1.3.7] | 0.526 | 1.85e-03 | 5.39e-02 |
| wbiB; dTDP-L-rhamnose 4-epimerase [EC:5.1.3.25] | -1.748 | 1.88e-03 | 5.44e-02 |
| pbp2A; penicillin-binding protein 2A [EC:2.4.1.129 3.4.16.4] | -0.948 | 1.88e-03 | 5.44e-02 |
| K22026; nucleoside kinase [EC:2.7.1.73 2.7.1.213 2.7.1.-] | 2.491 | 1.90e-03 | 5.46e-02 |
| ACR3, arsB; arsenite transporter | -1.464 | 1.92e-03 | 5.48e-02 |
| pepX; X-Pro dipeptidyl-peptidase [EC:3.4.14.11] | -0.954 | 1.93e-03 | 5.48e-02 |
| dapL; N-acetyldiaminopimelate deacetylase [EC:3.5.1.47] | -1.337 | 1.95e-03 | 5.51e-02 |
| MFS.CP; MFS transporter, CP family, cyanate transporter | -2.199 | 2.00e-03 | 5.58e-02 |
| pbpB; penicillin-binding protein 2B | -1.405 | 2.14e-03 | 5.93e-02 |
| cwlK; peptidoglycan LD-endopeptidase CwlK [EC:3.4.-.-] | -1.578 | 2.23e-03 | 6.15e-02 |
| fucT; alpha(1,3/1,4) fucosyltransferase [EC:2.4.1.65 2.4.1.152] | -3.415 | 2.25e-03 | 6.19e-02 |
| dnaB; replication initiation and membrane attachment protein | -1.959 | 2.33e-03 | 6.32e-02 |
| rhaS; AraC family transcriptional regulator, L-rhamnose operon regulatory protein RhaS | 1.256 | 2.41e-03 | 6.46e-02 |
| cobB-cbiA; cobyrinic acid a,c-diamide synthase [EC:6.3.5.9 6.3.5.11] | 0.540 | 2.42e-03 | 6.46e-02 |
| comD; two-component system, LytTR family, sensor histidine kinase ComD [EC:2.7.13.3] | -2.125 | 2.43e-03 | 6.46e-02 |
| yydK; GntR family transcriptional regulator, transcriptional regulator of bglA | -2.441 | 2.43e-03 | 6.46e-02 |
| hprA; glycerate dehydrogenase [EC:1.1.1.29] | 0.578 | 2.47e-03 | 6.50e-02 |
| waaH; heptose III glucuronosyltransferase [EC:2.4.1.-] | 2.144 | 2.47e-03 | 6.50e-02 |
| divIC, divA; cell division protein DivIC | -0.806 | 2.57e-03 | 6.73e-02 |
| ybiO; moderate conductance mechanosensitive channel | -0.945 | 2.64e-03 | 6.86e-02 |
| menF; menaquinone-specific isochorismate synthase [EC:5.4.4.2] | -1.086 | 2.67e-03 | 6.88e-02 |
| nifH; nitrogenase iron protein NifH | 2.500 | 2.72e-03 | 6.97e-02 |
| comGA; competence protein ComGA | -2.519 | 2.76e-03 | 7.03e-02 |
| zurR, zur; Fur family transcriptional regulator, zinc uptake regulator | -3.372 | 2.95e-03 | 7.49e-02 |
| yhgE; putative membrane protein | -1.155 | 2.97e-03 | 7.49e-02 |
| dltD; D-alanine transfer protein | -1.742 | 2.97e-03 | 7.49e-02 |
| mprF, fmtC; phosphatidylglycerol lysyltransferase [EC:2.3.2.3] | -0.931 | 3.10e-03 | 7.79e-02 |
| nlpD; lipoprotein NlpD | 0.543 | 3.13e-03 | 7.81e-02 |
| clfA; clumping factor A | -2.959 | 3.13e-03 | 7.81e-02 |
| higB; mRNA interferase HigB [EC:3.1.-.-] | 0.910 | 3.15e-03 | 7.82e-02 |
| ABC.GGU.S, chvE; putative multiple sugar transport system substrate-binding protein | 0.819 | 3.17e-03 | 7.82e-02 |
| arsA, ASNA1, GET3; arsenite/tail-anchored protein-transporting ATPase [EC:7.3.2.7 7.3.-.-] | 1.689 | 3.29e-03 | 8.04e-02 |
| pgpA; phosphatidylglycerophosphatase A [EC:3.1.3.27] | 0.902 | 3.32e-03 | 8.07e-02 |
| yefM; antitoxin YefM | 0.911 | 3.34e-03 | 8.08e-02 |
| ATPVE, ntpE, atpE; V/A-type H+/Na+-transporting ATPase subunit E | 0.598 | 3.51e-03 | 8.39e-02 |
| ntrY; two-component system, NtrC family, nitrogen regulation sensor histidine kinase NtrY [EC:2.7.13.3] | 0.914 | 3.55e-03 | 8.45e-02 |
| phnZ; 2-amino-1-hydroxyethylphosphonate dioxygenase (glycine-forming) [EC:1.13.11.78] | -3.135 | 3.58e-03 | 8.50e-02 |
| DLAT, aceF, pdhC; pyruvate dehydrogenase E2 component (dihydrolipoamide acetyltransferase) [EC:2.3.1.12] | -1.453 | 3.61e-03 | 8.50e-02 |
| K17203, eryF; erythritol transport system permease protein | -1.312 | 3.63e-03 | 8.52e-02 |
| ydhP; MFS transporter, DHA1 family, inner membrane transport protein | -1.021 | 3.65e-03 | 8.54e-02 |
| gltS; glutamate:Na+ symporter, ESS family | 0.551 | 3.92e-03 | 9.06e-02 |
| aadK; aminoglycoside 6-adenylyltransferase [EC:2.7.7.-] | 0.711 | 3.92e-03 | 9.06e-02 |
| cobA-hemD; uroporphyrinogen III methyltransferase / synthase [EC:2.1.1.107 4.2.1.75] | -0.879 | 4.02e-03 | 9.24e-02 |
| narH, narY, nxrB; nitrate reductase / nitrite oxidoreductase, beta subunit [EC:1.7.5.1 1.7.99.-] | -0.588 | 4.22e-03 | 9.69e-02 |
| yxdK; two-component system, OmpR family, sensor histidine kinase YxdK [EC:2.7.13.3] | -2.055 | 4.28e-03 | 9.77e-02 |
| coiA; competence protein CoiA | -2.139 | 4.29e-03 | 9.77e-02 |
| trmB; HTH-type transcriptional regulator, sugar sensing transcriptional regulator | 2.785 | 4.45e-03 | 1.01e-01 |
| bcs1; ribitol-5-phosphate 2-dehydrogenase (NADP+) / D-ribitol-5-phosphate cytidylyltransferase [EC:1.1.1.405 2.7.7.40] | 0.599 | 4.51e-03 | 1.02e-01 |
| inuJ; inulosucrase [EC:2.4.1.9] | -1.993 | 4.71e-03 | 1.05e-01 |
| kdgT; 2-keto-3-deoxygluconate permease | -1.642 | 4.74e-03 | 1.05e-01 |
| prlC; oligopeptidase A [EC:3.4.24.70] | -1.700 | 4.74e-03 | 1.05e-01 |
| metE; 5-methyltetrahydropteroyltriglutamate--homocysteine methyltransferase [EC:2.1.1.14] | 0.608 | 4.79e-03 | 1.06e-01 |
| oprO\_P; phosphate-selective porin OprO and OprP | -2.224 | 4.86e-03 | 1.07e-01 |
| dltB; membrane protein involved in D-alanine export | -0.944 | 4.90e-03 | 1.08e-01 |
| ABC.GGU.P, gguB; putative multiple sugar transport system permease protein | 0.841 | 5.14e-03 | 1.12e-01 |
| clpE; ATP-dependent Clp protease ATP-binding subunit ClpE | -0.838 | 5.29e-03 | 1.14e-01 |
| E2.7.4.2, mvaK2; phosphomevalonate kinase [EC:2.7.4.2] | -2.002 | 5.38e-03 | 1.15e-01 |
| gltC; LysR family transcriptional regulator, transcription activator of glutamate synthase operon | -1.289 | 5.38e-03 | 1.15e-01 |
| rpoE; DNA-directed RNA polymerase subunit delta | -0.738 | 5.65e-03 | 1.20e-01 |
| EBM; mannosylglycoprotein endo-beta-mannosidase [EC:3.2.1.152] | 1.293 | 5.86e-03 | 1.22e-01 |
| GSR, gor; glutathione reductase (NADPH) [EC:1.8.1.7] | -1.292 | 5.97e-03 | 1.24e-01 |
| agrC, blpH, fsrC; two-component system, LytTR family, sensor histidine kinase AgrC [EC:2.7.13.3] | 0.726 | 6.18e-03 | 1.28e-01 |
| rpfB; resuscitation-promoting factor RpfB | -2.075 | 6.47e-03 | 1.33e-01 |
| fruR2, fruR; DeoR family transcriptional regulator, fructose operon transcriptional repressor | -0.926 | 6.62e-03 | 1.35e-01 |
| asp2; accessory secretory protein Asp2 | -1.978 | 6.67e-03 | 1.36e-01 |
| dmsB; dimethyl sulfoxide reductase iron-sulfur subunit | -0.921 | 6.69e-03 | 1.36e-01 |
| metB; cystathionine gamma-synthase [EC:2.5.1.48] | 1.240 | 6.73e-03 | 1.36e-01 |
| puuE; 4-aminobutyrate aminotransferase [EC:2.6.1.19] | -0.960 | 6.77e-03 | 1.37e-01 |
| mtnE, mtnV; L-glutamine---4-(methylsulfanyl)-2-oxobutanoate aminotransferase [EC:2.6.1.117] | -1.873 | 6.84e-03 | 1.37e-01 |
| fucA; L-fuculose-phosphate aldolase [EC:4.1.2.17] | -0.544 | 6.97e-03 | 1.39e-01 |
| E2.7.1.12, gntK, idnK; gluconokinase [EC:2.7.1.12] | -1.528 | 7.26e-03 | 1.43e-01 |
| peb1B, glnP, glnM; aspartate/glutamate/glutamine transport system permease protein | -1.101 | 7.32e-03 | 1.44e-01 |
| yqeH; 30S ribosome assembly GTPase | -0.761 | 7.50e-03 | 1.47e-01 |
| lgaC; L-galactonate 5-dehydrogenase [EC:1.1.1.414] | -0.780 | 7.56e-03 | 1.47e-01 |
| pxpB; 5-oxoprolinase (ATP-hydrolysing) subunit B [EC:3.5.2.9] | 1.062 | 7.56e-03 | 1.47e-01 |
| E2.7.1.76, dak; deoxyadenosine kinase [EC:2.7.1.76] | -1.956 | 7.59e-03 | 1.47e-01 |
| idi, IDI; isopentenyl-diphosphate Delta-isomerase [EC:5.3.3.2] | -1.985 | 7.64e-03 | 1.48e-01 |
| comGB; competence protein ComGB | -1.943 | 7.70e-03 | 1.48e-01 |
| dinG; ATP-dependent DNA helicase DinG [EC:3.6.4.12] | -0.846 | 7.80e-03 | 1.49e-01 |
| cbiK; sirohydrochlorin cobaltochelatase [EC:4.99.1.3] | 0.509 | 7.83e-03 | 1.49e-01 |
| ribT; riboflavin biosynthesis RibT protein | -2.206 | 7.84e-03 | 1.49e-01 |
| yvaK; carboxylesterase [EC:3.1.1.1] | -2.729 | 7.86e-03 | 1.49e-01 |
| lplT; MFS transporter, LPLT family, lysophospholipid transporter | -1.379 | 7.99e-03 | 1.51e-01 |
| czrA; ArsR family transcriptional regulator, zinc-responsive transcriptional repressor | -1.780 | 8.28e-03 | 1.55e-01 |
| PMA1, PMA2; H+-transporting ATPase [EC:7.1.2.1] | -1.957 | 8.34e-03 | 1.56e-01 |
| cofE, fbiB; coenzyme F420-0:L-glutamate ligase / coenzyme F420-1:gamma-L-glutamate ligase [EC:6.3.2.31 6.3.2.34] | -3.019 | 8.54e-03 | 1.59e-01 |
| pxpA; 5-oxoprolinase (ATP-hydrolysing) subunit A [EC:3.5.2.9] | -0.785 | 8.61e-03 | 1.59e-01 |
| baiH; 7beta-hydroxy-3-oxochol-24-oyl-CoA 4-desaturase [EC:1.3.1.116] | 1.540 | 8.79e-03 | 1.62e-01 |
| K07318; adenine-specific DNA-methyltransferase [EC:2.1.1.72] | -0.858 | 8.83e-03 | 1.63e-01 |
| pbp1b; penicillin-binding protein 1B | -1.994 | 8.89e-03 | 1.63e-01 |
| adaB; methylated-DNA-[protein]-cysteine S-methyltransferase [EC:2.1.1.63] | -2.274 | 9.28e-03 | 1.67e-01 |
| E2.4.2.6; nucleoside deoxyribosyltransferase [EC:2.4.2.6] | -1.383 | 9.54e-03 | 1.71e-01 |
| pdaD; arginine decarboxylase [EC:4.1.1.19] | 1.673 | 9.80e-03 | 1.75e-01 |
| ihfB, himD; integration host factor subunit beta | -1.307 | 1.03e-02 | 1.81e-01 |
| tcyC, yecC; L-cystine transport system ATP-binding protein [EC:7.4.2.1] | -0.596 | 1.05e-02 | 1.85e-01 |
| ABC-2.CYL.P, cylB; multidrug/hemolysin transport system permease protein | -1.063 | 1.06e-02 | 1.86e-01 |
| pdtaR; two-component system, response regulator PdtaR | 0.628 | 1.06e-02 | 1.86e-01 |
| araN; arabinosaccharide transport system substrate-binding protein | 1.028 | 1.08e-02 | 1.88e-01 |
| eryE; erythritol transport system ATP-binding protein | -1.222 | 1.09e-02 | 1.90e-01 |
| waaZ, rfaZ; KDO transferase III [EC:2.4.99.-] | -2.240 | 1.09e-02 | 1.90e-01 |
| K09155; uncharacterized protein | -1.890 | 1.10e-02 | 1.91e-01 |
| fctD; glutamate formiminotransferase / 5-formyltetrahydrofolate cyclo-ligase [EC:2.1.2.5 6.3.3.2] | -1.019 | 1.12e-02 | 1.93e-01 |
| gmhD, rfaD; ADP-L-glycero-D-manno-heptose 6-epimerase [EC:5.1.3.20] | -0.912 | 1.18e-02 | 2.01e-01 |
| mdtG; MFS transporter, DHA1 family, multidrug resistance protein | -0.685 | 1.18e-02 | 2.02e-01 |
| dhaT; 1,3-propanediol dehydrogenase [EC:1.1.1.202] | -1.957 | 1.20e-02 | 2.03e-01 |
| PCCB, pccB; propionyl-CoA carboxylase beta chain [EC:6.4.1.3 2.1.3.15] | 0.670 | 1.24e-02 | 2.09e-01 |
| ygaC; uncharacterized protein | -0.713 | 1.25e-02 | 2.10e-01 |
| glnE; [glutamine synthetase] adenylyltransferase / [glutamine synthetase]-adenylyl-L-tyrosine phosphorylase [EC:2.7.7.42 2.7.7.89] | -1.174 | 1.25e-02 | 2.10e-01 |
| cysE; serine O-acetyltransferase [EC:2.3.1.30] | -2.171 | 1.26e-02 | 2.10e-01 |
| DPEP; membrane dipeptidase [EC:3.4.13.19] | 1.123 | 1.27e-02 | 2.11e-01 |
| lptD, imp, ostA; LPS-assembly protein | -1.628 | 1.28e-02 | 2.11e-01 |
| menH; 2-succinyl-6-hydroxy-2,4-cyclohexadiene-1-carboxylate synthase [EC:4.2.99.20] | -1.785 | 1.28e-02 | 2.11e-01 |
| racD; aspartate racemase [EC:5.1.1.13] | -0.649 | 1.28e-02 | 2.11e-01 |
| fer; ferredoxin | -0.644 | 1.30e-02 | 2.13e-01 |
| pepD; putative serine protease PepD [EC:3.4.21.-] | -1.557 | 1.30e-02 | 2.13e-01 |
| K07487; transposase | 1.409 | 1.30e-02 | 2.13e-01 |
| rsmB, sun; 16S rRNA (cytosine967-C5)-methyltransferase [EC:2.1.1.176] | -0.564 | 1.32e-02 | 2.15e-01 |
| hemC, HMBS; hydroxymethylbilane synthase [EC:2.5.1.61] | -0.608 | 1.32e-02 | 2.15e-01 |
| psp; phosphoserine phosphatase [EC:3.1.3.3] | 1.361 | 1.37e-02 | 2.21e-01 |
| pncC; nicotinamide-nucleotide amidase [EC:3.5.1.42] | 0.624 | 1.37e-02 | 2.21e-01 |
| dotB, traJ; defect in organelle trafficking protein DotB [EC:7.2.4.8] | -1.953 | 1.41e-02 | 2.25e-01 |
| mcsB; protein arginine kinase [EC:2.7.14.1] | -1.463 | 1.44e-02 | 2.30e-01 |
| yahK; alcohol dehydrogenase (NADP+) [EC:1.1.1.2] | -1.227 | 1.45e-02 | 2.30e-01 |
| lacD; tagatose 1,6-diphosphate aldolase [EC:4.1.2.40] | -1.713 | 1.45e-02 | 2.31e-01 |
| adhP; alcohol dehydrogenase, propanol-preferring [EC:1.1.1.1] | -0.662 | 1.46e-02 | 2.31e-01 |
| sbp; sulfate/thiosulfate transport system substrate-binding protein | 1.274 | 1.49e-02 | 2.35e-01 |
| mvaA; hydroxymethylglutaryl-CoA reductase [EC:1.1.1.88] | -0.750 | 1.49e-02 | 2.35e-01 |
| GATM; glycine amidinotransferase [EC:2.1.4.1] | -0.788 | 1.58e-02 | 2.46e-01 |
| purSL; phosphoribosylformylglycinamidine synthase subunit PurSL [EC:6.3.5.3] | -1.724 | 1.59e-02 | 2.47e-01 |
| cysI; sulfite reductase (NADPH) hemoprotein beta-component [EC:1.8.1.2] | -2.073 | 1.61e-02 | 2.49e-01 |
| dnr; CRP/FNR family transcriptional regulator, dissimilatory nitrate respiration regulator | 2.341 | 1.61e-02 | 2.49e-01 |
| K07219; putative molybdopterin biosynthesis protein | -1.558 | 1.62e-02 | 2.49e-01 |
| araP; arabinosaccharide transport system permease protein | 0.919 | 1.62e-02 | 2.49e-01 |
| wcaI; colanic acid biosynthesis glycosyl transferase WcaI | 1.504 | 1.63e-02 | 2.49e-01 |
| E2.3.3.10; hydroxymethylglutaryl-CoA synthase [EC:2.3.3.10] | -0.781 | 1.66e-02 | 2.53e-01 |
| betB, gbsA; betaine-aldehyde dehydrogenase [EC:1.2.1.8] | 1.205 | 1.70e-02 | 2.57e-01 |
| pseI, neuB3; pseudaminic acid synthase [EC:2.5.1.97] | 0.686 | 1.71e-02 | 2.58e-01 |
| ycbB, glnL; two-component system, response regulator YcbB | -2.007 | 1.74e-02 | 2.63e-01 |
| dbpA; ATP-dependent RNA helicase DbpA [EC:3.6.4.13] | -0.572 | 1.84e-02 | 2.78e-01 |
| ydjE; MFS transporter, putative metabolite:H+ symporter | -1.161 | 1.87e-02 | 2.81e-01 |
| CBS; cystathionine beta-synthase [EC:4.2.1.22] | -1.998 | 1.90e-02 | 2.84e-01 |
| yagU; putative membrane protein | -2.166 | 1.99e-02 | 2.94e-01 |
| cggR; central glycolytic genes regulator | -1.091 | 1.99e-02 | 2.94e-01 |
| proP; MFS transporter, MHS family, proline/betaine transporter | -0.825 | 2.01e-02 | 2.96e-01 |
| triA; membrane fusion protein, multidrug efflux system | 1.895 | 2.03e-02 | 2.97e-01 |
| qrtT; energy-coupling factor transport system substrate-specific component | -1.526 | 2.04e-02 | 2.98e-01 |
| ftsL; cell division protein FtsL | -2.185 | 2.05e-02 | 2.98e-01 |
| mscK, kefA, aefA; potassium-dependent mechanosensitive channel | -1.094 | 2.05e-02 | 2.98e-01 |
| nodU; carbamoyltransferase [EC:2.1.3.-] | 1.015 | 2.07e-02 | 3.00e-01 |
| cysU; sulfate/thiosulfate transport system permease protein | 1.348 | 2.09e-02 | 3.02e-01 |
| OGDH, sucA; 2-oxoglutarate dehydrogenase E1 component [EC:1.2.4.2] | -0.997 | 2.10e-02 | 3.02e-01 |
| mobA; molybdenum cofactor guanylyltransferase [EC:2.7.7.77] | -1.338 | 2.13e-02 | 3.04e-01 |
| ABC.GGU.A, gguA; putative multiple sugar transport system ATP-binding protein [EC:7.5.2.-] | 0.588 | 2.14e-02 | 3.04e-01 |
| phnE; phosphonate transport system permease protein | -1.103 | 2.14e-02 | 3.04e-01 |
| K07078; uncharacterized protein | 0.682 | 2.15e-02 | 3.04e-01 |
| lysP; lysine-specific permease | -0.665 | 2.17e-02 | 3.04e-01 |
| GALT29A; beta-1,6-galactosyltransferase [EC:2.4.1.-] | 1.644 | 2.17e-02 | 3.04e-01 |
| lpxJ; Kdo2-lipid IVA 3' secondary acyltransferase [EC:2.3.1.-] | -2.927 | 2.18e-02 | 3.04e-01 |
| argO; N-acetylglutamate synthase [EC:2.3.1.1] | 0.565 | 2.23e-02 | 3.09e-01 |
| gmhC, hldE, waaE, rfaE; D-beta-D-heptose 7-phosphate kinase / D-beta-D-heptose 1-phosphate adenosyltransferase [EC:2.7.1.167 2.7.7.70] | -1.166 | 2.24e-02 | 3.09e-01 |
| srlE; glucitol/sorbitol PTS system EIIB component [EC:2.7.1.198] | 0.822 | 2.26e-02 | 3.12e-01 |
| otnC; 3-dehydro-4-phosphotetronate decarboxylase [EC:4.1.1.104] | -0.540 | 2.27e-02 | 3.13e-01 |
| narJ, narW; nitrate reductase molybdenum cofactor assembly chaperone NarJ/NarW | -1.656 | 2.30e-02 | 3.16e-01 |
| mntB; manganese transport system permease protein | -2.701 | 2.31e-02 | 3.17e-01 |
| E2.7.11.1; non-specific serine/threonine protein kinase [EC:2.7.11.1] | -0.840 | 2.34e-02 | 3.19e-01 |
| alsD, budA, aldC; acetolactate decarboxylase [EC:4.1.1.5] | -0.852 | 2.35e-02 | 3.21e-01 |
| ripA; peptidoglycan DL-endopeptidase RipA [EC:3.4.-.-] | -2.019 | 2.36e-02 | 3.21e-01 |
| K09790; uncharacterized protein | 0.692 | 2.36e-02 | 3.21e-01 |
| gabD; succinate-semialdehyde dehydrogenase / glutarate-semialdehyde dehydrogenase [EC:1.2.1.16 1.2.1.79 1.2.1.20] | -1.228 | 2.40e-02 | 3.25e-01 |
| treZ, glgZ; maltooligosyltrehalose trehalohydrolase [EC:3.2.1.141] | -1.173 | 2.42e-02 | 3.27e-01 |
| DHODH, pyrD; dihydroorotate dehydrogenase [EC:1.3.5.2] | -0.751 | 2.44e-02 | 3.27e-01 |
| rlpA; rare lipoprotein A | -0.895 | 2.44e-02 | 3.27e-01 |
| hydA; sulfhydrogenase subunit alpha [EC:1.12.1.3 1.12.1.5] | -1.080 | 2.45e-02 | 3.28e-01 |
| lctO; L-lactate oxidase [EC:1.1.3.2] | -1.797 | 2.49e-02 | 3.30e-01 |
| nnrD; ADP-dependent NAD(P)H-hydrate dehydratase [EC:4.2.1.136] | -1.628 | 2.50e-02 | 3.30e-01 |
| K09133; uncharacterized protein | -0.866 | 2.50e-02 | 3.30e-01 |
| pabC; 4-amino-4-deoxychorismate lyase [EC:4.1.3.38] | 0.646 | 2.58e-02 | 3.38e-01 |
| E2.7.1.36, MVK, mvaK1; mevalonate kinase [EC:2.7.1.36] | -1.436 | 2.59e-02 | 3.38e-01 |
| yejA; microcin C transport system substrate-binding protein | -2.597 | 2.60e-02 | 3.40e-01 |
| dpe, lre; D-psicose/D-tagatose/L-ribulose 3-epimerase [EC:5.1.3.30 5.1.3.31] | 0.728 | 2.68e-02 | 3.46e-01 |
| ptrB; oligopeptidase B [EC:3.4.21.83] | -2.528 | 2.69e-02 | 3.46e-01 |
| znuA; zinc transport system substrate-binding protein | 0.562 | 2.79e-02 | 3.57e-01 |
| cysJ; sulfite reductase (NADPH) flavoprotein alpha-component [EC:1.8.1.2] | -2.089 | 2.85e-02 | 3.63e-01 |
| spxA; regulatory protein spx | -0.706 | 2.87e-02 | 3.65e-01 |
| SLC9B1\_2; solute carrier family 9B (sodium/hydrogen exchanger), member 1/2 | -0.789 | 2.93e-02 | 3.71e-01 |
| mepR; MarR family transcriptional regulator, repressor for mepA | 0.943 | 2.94e-02 | 3.72e-01 |
| biuH; biuret amidohydrolase [EC:3.5.1.84] | -1.469 | 2.98e-02 | 3.74e-01 |
| bglB; beta-glucosidase [EC:3.2.1.21] | 0.677 | 3.02e-02 | 3.79e-01 |
| ppsR; [pyruvate, water dikinase]-phosphate phosphotransferase / [pyruvate, water dikinase] kinase [EC:2.7.4.28 2.7.11.33] | -0.583 | 3.04e-02 | 3.81e-01 |
| ecnB; entericidin B | -2.086 | 3.07e-02 | 3.84e-01 |
| hepST; heptaprenyl diphosphate synthase [EC:2.5.1.30] | -0.736 | 3.12e-02 | 3.89e-01 |
| MVD, mvaD; diphosphomevalonate decarboxylase [EC:4.1.1.33] | -0.910 | 3.13e-02 | 3.89e-01 |
| ptsN; nitrogen PTS system EIIA component [EC:2.7.1.-] | -2.003 | 3.15e-02 | 3.91e-01 |
| creD; inner membrane protein | -0.535 | 3.19e-02 | 3.95e-01 |
| pilB; type IV pilus assembly protein PilB | -0.682 | 3.27e-02 | 4.03e-01 |
| hemA; glutamyl-tRNA reductase [EC:1.2.1.70] | -0.834 | 3.32e-02 | 4.08e-01 |
| tesB; acyl-CoA thioesterase II [EC:3.1.2.-] | -2.437 | 3.34e-02 | 4.09e-01 |
| RP-S2e, RPS2; small subunit ribosomal protein S2e | 1.611 | 3.34e-02 | 4.09e-01 |
| trm14; tRNA (guanine6-N2)-methyltransferase [EC:2.1.1.256] | 2.283 | 3.36e-02 | 4.10e-01 |
| mdeA; MFS transporter, DHA2 family, multidrug resistance protein | -1.858 | 3.38e-02 | 4.12e-01 |
| TC.CPA1; monovalent cation:H+ antiporter, CPA1 family | -1.238 | 3.39e-02 | 4.13e-01 |
| kdgR; LacI family transcriptional regulator, kdg operon repressor | -1.166 | 3.41e-02 | 4.14e-01 |
| srtA; sortase A [EC:3.4.22.70] | 0.693 | 3.48e-02 | 4.21e-01 |
| yxiD; toxin YxiD [EC:3.1.-.-] | -2.268 | 3.49e-02 | 4.22e-01 |
| dge1; diguanylate cyclase [EC:2.7.7.65] | -2.128 | 3.57e-02 | 4.29e-01 |
| pcaK; MFS transporter, AAHS family, 4-hydroxybenzoate transporter | -1.089 | 3.59e-02 | 4.30e-01 |
| mgsC; methylamine---glutamate N-methyltransferase subunit C [EC:2.1.1.21] | 0.525 | 3.60e-02 | 4.30e-01 |
| lapA; lipopolysaccharide assembly protein A | -2.867 | 3.63e-02 | 4.31e-01 |
| cysW; sulfate/thiosulfate transport system permease protein | 1.139 | 3.63e-02 | 4.31e-01 |
| nifA; Nif-specific regulatory protein | -0.788 | 3.65e-02 | 4.32e-01 |
| tet; tetrahedral aminopeptidase [EC:3.4.11.-] | -2.749 | 3.72e-02 | 4.39e-01 |
| ynfE; Tat-targeted selenate reductase subunit YnfE [EC:1.97.1.9] | -0.615 | 3.73e-02 | 4.40e-01 |
| pimA; phosphatidyl-myo-inositol alpha-mannosyltransferase [EC:2.4.1.345] | -2.860 | 3.77e-02 | 4.40e-01 |
| ompR; two-component system, OmpR family, phosphate regulon response regulator OmpR | 0.513 | 3.78e-02 | 4.41e-01 |
| argAB; amino-acid N-acetyltransferase [EC:2.3.1.1] | -1.212 | 3.81e-02 | 4.43e-01 |
| galP; MFS transporter, SP family, galactose:H+ symporter | 1.973 | 3.93e-02 | 4.55e-01 |
| QPCT; glutaminyl-peptide cyclotransferase [EC:2.3.2.5] | -0.723 | 3.93e-02 | 4.55e-01 |
| aphA; PadR family transcriptional regulator, regulatory protein AphA | -1.190 | 3.95e-02 | 4.56e-01 |
| K09805; uncharacterized protein | 2.064 | 3.96e-02 | 4.58e-01 |
| kamA; lysine 2,3-aminomutase [EC:5.4.3.2] | 0.905 | 3.99e-02 | 4.59e-01 |
| rnhC; ribonuclease HIII [EC:3.1.26.4] | -1.653 | 4.00e-02 | 4.61e-01 |
| adh; isopropanol dehydrogenase (NADP+) [EC:1.1.1.80] | 1.128 | 4.03e-02 | 4.63e-01 |
| echC; ech hydrogenase subunit C | -0.960 | 4.07e-02 | 4.66e-01 |
| ABC.MN.A; manganese/iron transport system ATP-binding protein | -2.811 | 4.09e-02 | 4.66e-01 |
| aroKB; shikimate kinase / 3-dehydroquinate synthase [EC:2.7.1.71 4.2.3.4] | -2.922 | 4.10e-02 | 4.66e-01 |
| fdhD; FdhD protein | -1.580 | 4.14e-02 | 4.69e-01 |
| helY; ATP-dependent RNA helicase HelY [EC:3.6.4.-] | -2.202 | 4.15e-02 | 4.69e-01 |
| CDC5L, CDC5, CEF1; pre-mRNA-splicing factor CDC5/CEF1 | 1.717 | 4.15e-02 | 4.69e-01 |
| radD; DNA repair protein RadD | 0.658 | 4.17e-02 | 4.70e-01 |
| K02482; two-component system, NtrC family, sensor kinase [EC:2.7.13.3] | 1.113 | 4.22e-02 | 4.73e-01 |
| pbpA; penicillin-binding protein A | -0.694 | 4.22e-02 | 4.73e-01 |
| K09775; uncharacterized protein | -0.657 | 4.22e-02 | 4.73e-01 |
| moaB; molybdopterin adenylyltransferase [EC:2.7.7.75] | -2.365 | 4.26e-02 | 4.76e-01 |
| pilC; type IV pilus assembly protein PilC | -0.704 | 4.27e-02 | 4.76e-01 |
| phnD; phosphonate transport system substrate-binding protein | -0.936 | 4.37e-02 | 4.82e-01 |
| glcD; glycolate oxidase [EC:1.1.3.15] | -0.678 | 4.46e-02 | 4.89e-01 |
| E1.2.3.3, poxL; pyruvate oxidase [EC:1.2.3.3] | -1.077 | 4.50e-02 | 4.93e-01 |
| cobA, btuR; cob(I)alamin adenosyltransferase [EC:2.5.1.17] | 0.544 | 4.53e-02 | 4.94e-01 |
| cpt; chloramphenicol 3-O phosphotransferase [EC:2.7.1.-] | -1.066 | 4.57e-02 | 4.97e-01 |
| NUDT2; bis(5'-nucleosidyl)-tetraphosphatase [EC:3.6.1.17] | -1.735 | 4.61e-02 | 5.00e-01 |
| sanA; SanA protein | -0.673 | 4.65e-02 | 5.02e-01 |
| trpGD; anthranilate synthase/phosphoribosyltransferase [EC:4.1.3.27 2.4.2.18] | -1.302 | 4.67e-02 | 5.04e-01 |
| bmaC; fibronectin-binding autotransporter adhesin | -2.176 | 4.68e-02 | 5.04e-01 |
| iolT; MFS transporter, SP family, major inositol transporter | -2.272 | 4.69e-02 | 5.04e-01 |
| narI, narV; nitrate reductase gamma subunit [EC:1.7.5.1 1.7.99.-] | -0.893 | 4.75e-02 | 5.09e-01 |
| araM, egsA; glycerol-1-phosphate dehydrogenase [NAD(P)+] [EC:1.1.1.261] | 0.559 | 4.79e-02 | 5.12e-01 |
| cutF, nlpE; copper homeostasis protein (lipoprotein) | -1.574 | 4.85e-02 | 5.16e-01 |
| kal; 3-aminobutyryl-CoA ammonia-lyase [EC:4.3.1.14] | -1.030 | 4.93e-02 | 5.22e-01 |
| senX3; two-component system, OmpR family, sensor histidine kinase SenX3 [EC:2.7.13.3] | 1.158 | 4.95e-02 | 5.23e-01 |
| H3; histone H3 | 0.766 | 4.95e-02 | 5.23e-01 |
| GLUD1\_2, gdhA; glutamate dehydrogenase (NAD(P)+) [EC:1.4.1.3] | 1.040 | 4.96e-02 | 5.23e-01 |
| PGC-1α-C after vs. Wt-C after | | | |
| glyQ; glycyl-tRNA synthetase alpha chain [EC:6.1.1.14] | -0.711 | 1.06e-08 | 2.44e-05\* |
| lolC\_E; lipoprotein-releasing system permease protein | 0.545 | 1.21e-07 | 1.58e-04\* |
| K07148; uncharacterized protein | 0.637 | 1.37e-07 | 1.58e-04\* |
| ARSA; arylsulfatase A [EC:3.1.6.8] | 0.687 | 2.04e-07 | 2.01e-04\* |
| ptsG, glcA, glcB; glucose PTS system EIICBA or EIICB component [EC:2.7.1.199] | -0.651 | 3.26e-07 | 2.52e-04\* |
| K07497; putative transposase | -0.711 | 4.35e-07 | 3.00e-04\* |
| PC, pyc; pyruvate carboxylase [EC:6.4.1.1] | -1.186 | 6.46e-07 | 4.05e-04\* |
| K07483; transposase | -1.004 | 1.03e-06 | 5.38e-04\* |
| fruA; fructose PTS system EIIBC or EIIC component [EC:2.7.1.202] | -0.598 | 1.09e-06 | 5.38e-04\* |
| ulaA, sgaT; ascorbate PTS system EIIC component | -1.225 | 1.17e-06 | 5.39e-04\* |
| dexA; dextranase [EC:3.2.1.11] | 0.756 | 1.58e-06 | 6.82e-04\* |
| K07496; putative transposase | -1.164 | 2.21e-06 | 8.48e-04\* |
| npr; NADH peroxidase [EC:1.11.1.1] | -1.803 | 4.09e-06 | 1.43e-03\* |
| yxjA, nupG; purine nucleoside transport protein | -1.527 | 4.52e-06 | 1.49e-03\* |
| K07097; uncharacterized protein | -1.548 | 6.86e-06 | 1.96e-03\* |
| E3.1.3.41; 4-nitrophenyl phosphatase [EC:3.1.3.41] | -0.886 | 7.08e-06 | 1.96e-03\* |
| K09704; uncharacterized protein | 0.516 | 8.19e-06 | 2.17e-03\* |
| nadE; NAD+ synthase [EC:6.3.1.5] | -0.954 | 1.01e-05 | 2.49e-03\* |
| clpL; ATP-dependent Clp protease ATP-binding subunit ClpL | -1.337 | 1.05e-05 | 2.50e-03\* |
| lysP; lysine-specific permease | -1.296 | 1.20e-05 | 2.76e-03\* |
| IGHMBP2; ATP-dependent RNA/DNA helicase IGHMBP2 [EC:3.6.4.12 3.6.4.13] | 0.732 | 1.27e-05 | 2.83e-03\* |
| recU; recombination protein U | -0.821 | 1.35e-05 | 2.91e-03\* |
| K07498; putative transposase | -1.901 | 1.93e-05 | 3.92e-03\* |
| VIT; vacuolar iron transporter family protein | -1.266 | 2.13e-05 | 4.03e-03\* |
| NAGLU; alpha-N-acetylglucosaminidase [EC:3.2.1.50] | 0.813 | 2.14e-05 | 4.03e-03\* |
| scrA, sacP, sacX, ptsS; sucrose PTS system EIIBCA or EIIBC component [EC:2.7.1.211] | -0.761 | 2.22e-05 | 4.03e-03\* |
| ppsR; [pyruvate, water dikinase]-phosphate phosphotransferase / [pyruvate, water dikinase] kinase [EC:2.7.4.28 2.7.11.33] | -0.800 | 2.29e-05 | 4.05e-03\* |
| czcA, cusA, cnrA; heavy metal efflux system protein | 0.892 | 2.35e-05 | 4.06e-03\* |
| ulaD, sgaH, sgbH; 3-dehydro-L-gulonate-6-phosphate decarboxylase [EC:4.1.1.85] | -1.903 | 2.86e-05 | 4.66e-03\* |
| tuaB; teichuronic acid exporter | 0.631 | 3.38e-05 | 5.07e-03\* |
| malT; maltose PTS system EIICB or EIICBA component [EC:2.7.1.208] | -1.009 | 3.50e-05 | 5.14e-03\* |
| fucO; lactaldehyde reductase [EC:1.1.1.77] | -0.622 | 3.91e-05 | 5.51e-03\* |
| K09955; uncharacterized protein | 0.514 | 4.15e-05 | 5.65e-03\* |
| K07450; putative resolvase | -1.064 | 4.17e-05 | 5.65e-03\* |
| casC, cse4; CRISPR system Cascade subunit CasC | -1.968 | 4.34e-05 | 5.76e-03\* |
| GBA, srfJ; glucosylceramidase [EC:3.2.1.45] | 0.613 | 4.46e-05 | 5.80e-03\* |
| yfeX; porphyrinogen peroxidase [EC:1.11.1.-] | -1.263 | 4.54e-05 | 5.80e-03\* |
| adhP; alcohol dehydrogenase, propanol-preferring [EC:1.1.1.1] | -1.033 | 4.71e-05 | 5.91e-03\* |
| hya; hyaluronoglucosaminidase [EC:3.2.1.35] | 0.585 | 5.15e-05 | 6.33e-03\* |
| K06962; uncharacterized protein | -1.425 | 5.23e-05 | 6.33e-03\* |
| glcR; DeoR family transcriptional regulator, carbon catabolite repression regulator | -1.809 | 5.61e-05 | 6.58e-03\* |
| K06937; 7,8-dihydro-6-hydroxymethylpterin dimethyltransferase [EC:2.1.1.-] | -1.760 | 5.98e-05 | 6.88e-03\* |
| spoVS; stage V sporulation protein S | -1.213 | 6.14e-05 | 6.93e-03\* |
| garD; galactarate dehydratase [EC:4.2.1.42] | -1.352 | 6.32e-05 | 6.93e-03\* |
| mleA, mleS; malolactic enzyme [EC:4.1.1.101] | -1.275 | 6.57e-05 | 7.09e-03\* |
| asl; D-aspartate ligase [EC:6.3.1.12] | -0.850 | 7.17e-05 | 7.62e-03\* |
| celA, chbB; cellobiose PTS system EIIB component [EC:2.7.1.196 2.7.1.205] | -1.072 | 7.39e-05 | 7.73e-03\* |
| vanY; zinc D-Ala-D-Ala carboxypeptidase [EC:3.4.17.14] | -1.200 | 7.82e-05 | 7.94e-03\* |
| glvC, malP, aglA; alpha-glucoside PTS system EIICB component [EC:2.7.1.208 2.7.1.-] | -1.266 | 8.15e-05 | 8.06e-03\* |
| lacR; DeoR family transcriptional regulator, lactose phosphotransferase system repressor | -4.286 | 8.19e-05 | 8.06e-03\* |
| patB, rscB, lmrC, satB; ATP-binding cassette, subfamily B, multidrug efflux pump | -1.309 | 8.58e-05 | 8.23e-03\* |
| hemE, UROD; uroporphyrinogen decarboxylase [EC:4.1.1.37] | -0.726 | 9.39e-05 | 8.88e-03\* |
| fruK; 1-phosphofructokinase [EC:2.7.1.56] | -0.648 | 1.11e-04 | 1.02e-02\* |
| aor; aldehyde:ferredoxin oxidoreductase [EC:1.2.7.5] | -0.906 | 1.14e-04 | 1.03e-02\* |
| manX; mannose PTS system EIIAB component [EC:2.7.1.191] | -0.832 | 1.18e-04 | 1.04e-02\* |
| E1.7.1.7, guaC; GMP reductase [EC:1.7.1.7] | -0.715 | 1.19e-04 | 1.04e-02\* |
| purR; purine operon repressor | -1.342 | 1.28e-04 | 1.11e-02\* |
| nupC; nucleoside transport protein | -1.610 | 1.44e-04 | 1.19e-02\* |
| fdhF; formate dehydrogenase (acceptor) [EC:1.17.99.7] | -1.257 | 1.52e-04 | 1.25e-02\* |
| E1.12.7.2L; ferredoxin hydrogenase large subunit [EC:1.12.7.2] | -1.125 | 1.54e-04 | 1.25e-02\* |
| yydH; putative peptide zinc metalloprotease protein | -1.753 | 1.60e-04 | 1.28e-02\* |
| lipB; lipoyl(octanoyl) transferase [EC:2.3.1.181] | 0.809 | 1.63e-04 | 1.30e-02\* |
| purL; phosphoribosylformylglycinamidine synthase subunit PurL [EC:6.3.5.3] | -1.151 | 1.68e-04 | 1.31e-02\* |
| echE; ech hydrogenase subunit E | -1.144 | 1.79e-04 | 1.36e-02\* |
| tagF; CDP-glycerol glycerophosphotransferase [EC:2.7.8.12] | -1.139 | 1.79e-04 | 1.36e-02\* |
| cimA; (R)-citramalate synthase [EC:2.3.1.182] | 0.521 | 1.94e-04 | 1.41e-02\* |
| iscR; Rrf2 family transcriptional regulator, iron-sulfur cluster assembly transcription factor | -0.552 | 1.97e-04 | 1.41e-02\* |
| ushA; 5'-nucleotidase / UDP-sugar diphosphatase [EC:3.1.3.5 3.6.1.45] | 0.555 | 2.33e-04 | 1.63e-02\* |
| GSP13; general stress protein 13 | -1.581 | 2.39e-04 | 1.63e-02\* |
| mutM, fpg; formamidopyrimidine-DNA glycosylase [EC:3.2.2.23 4.2.99.18] | -0.878 | 2.79e-04 | 1.80e-02\* |
| PDHB, pdhB; pyruvate dehydrogenase E1 component beta subunit [EC:1.2.4.1] | -1.275 | 2.79e-04 | 1.80e-02\* |
| celC, chbA; cellobiose PTS system EIIA component [EC:2.7.1.196 2.7.1.205] | -1.639 | 2.80e-04 | 1.80e-02\* |
| E3.1.2.20; acyl-CoA hydrolase [EC:3.1.2.20] | -2.218 | 2.82e-04 | 1.80e-02\* |
| pilB; type IV pilus assembly protein PilB | 0.755 | 2.92e-04 | 1.85e-02\* |
| abnA; arabinan endo-1,5-alpha-L-arabinosidase [EC:3.2.1.99] | 0.794 | 3.06e-04 | 1.92e-02\* |
| yraN; putative endonuclease | 0.618 | 3.13e-04 | 1.94e-02\* |
| ulaG; L-ascorbate 6-phosphate lactonase [EC:3.1.1.-] | -0.711 | 3.38e-04 | 2.01e-02\* |
| cpoA; 1,2-diacylglycerol-3-alpha-glucose alpha-1,2-galactosyltransferase [EC:2.4.1.-] | -1.242 | 3.67e-04 | 2.13e-02\* |
| aacC; aminoglycoside 3-N-acetyltransferase [EC:2.3.1.81] | -1.682 | 4.15e-04 | 2.35e-02\* |
| dltC; D-alanine--poly(phosphoribitol) ligase subunit 2 [EC:6.1.1.13] | -1.369 | 4.18e-04 | 2.35e-02\* |
| araB; L-ribulokinase [EC:2.7.1.16] | -0.661 | 4.21e-04 | 2.35e-02\* |
| PDHA, pdhA; pyruvate dehydrogenase E1 component alpha subunit [EC:1.2.4.1] | -1.065 | 4.23e-04 | 2.35e-02\* |
| DPP4, CD26; dipeptidyl-peptidase 4 [EC:3.4.14.5] | 0.647 | 4.34e-04 | 2.35e-02\* |
| K07571; S1 RNA binding domain protein | -0.677 | 4.43e-04 | 2.35e-02\* |
| chbG; chitin disaccharide deacetylase [EC:3.5.1.105] | -2.953 | 4.47e-04 | 2.35e-02\* |
| nikE, cntF; nickel transport system ATP-binding protein [EC:7.2.2.11] | -3.751 | 4.49e-04 | 2.35e-02\* |
| lpxK; tetraacyldisaccharide 4'-kinase [EC:2.7.1.130] | 0.735 | 4.51e-04 | 2.35e-02\* |
| glvA; maltose-6'-phosphate glucosidase [EC:3.2.1.122] | -0.891 | 4.52e-04 | 2.35e-02\* |
| K09963; uncharacterized protein | -1.417 | 5.21e-04 | 2.64e-02\* |
| ydhQ; GntR family transcriptional regulator | -1.111 | 5.33e-04 | 2.68e-02\* |
| tcyB, yecS; L-cystine transport system permease protein | -0.816 | 5.42e-04 | 2.69e-02\* |
| lpxB; lipid-A-disaccharide synthase [EC:2.4.1.182] | 0.634 | 5.55e-04 | 2.74e-02\* |
| E3.4.17.19; carboxypeptidase Taq [EC:3.4.17.19] | -0.834 | 5.80e-04 | 2.80e-02\* |
| proP; MFS transporter, MHS family, proline/betaine transporter | -1.064 | 6.07e-04 | 2.88e-02\* |
| mapP; maltose 6'-phosphate phosphatase [EC:3.1.3.90] | -3.177 | 6.74e-04 | 3.14e-02\* |
| K06993; ribonuclease H-related protein | -1.115 | 6.97e-04 | 3.21e-02\* |
| vicK; two-component system, OmpR family, sensor histidine kinase VicK [EC:2.7.13.3] | -0.511 | 7.09e-04 | 3.24e-02\* |
| E2.3.3.10; hydroxymethylglutaryl-CoA synthase [EC:2.3.3.10] | -1.057 | 7.20e-04 | 3.25e-02\* |
| wlbA, bplA; UDP-N-acetyl-2-amino-2-deoxyglucuronate dehydrogenase [EC:1.1.1.335] | -0.806 | 7.42e-04 | 3.31e-02\* |
| GLDC, gcvP; glycine dehydrogenase [EC:1.4.4.2] | 0.526 | 8.16e-04 | 3.59e-02\* |
| manY; mannose PTS system EIIC component | -0.691 | 8.54e-04 | 3.64e-02\* |
| croR; 3-hydroxybutyryl-CoA dehydratase [EC:4.2.1.55] | -2.013 | 8.70e-04 | 3.68e-02\* |
| urtA; urea transport system substrate-binding protein | 2.708 | 9.38e-04 | 3.87e-02\* |
| vioA; dTDP-4-amino-4,6-dideoxy-D-glucose transaminase [EC:2.6.1.33] | 0.755 | 9.49e-04 | 3.87e-02\* |
| ynfE; Tat-targeted selenate reductase subunit YnfE [EC:1.97.1.9] | -1.346 | 9.52e-04 | 3.87e-02\* |
| enr; 2-enoate reductase [EC:1.3.1.31] | -0.679 | 9.53e-04 | 3.87e-02\* |
| cshB; ATP-dependent RNA helicase CshB [EC:3.6.4.13] | -1.009 | 9.69e-04 | 3.91e-02\* |
| yxdJ; two-component system, OmpR family, response regulator YxdJ | -1.485 | 1.00e-03 | 4.02e-02\* |
| ywaD; aminopeptidase YwaD [EC:3.4.11.6 3.4.11.10] | -1.224 | 1.02e-03 | 4.04e-02\* |
| fkpA; FKBP-type peptidyl-prolyl cis-trans isomerase FkpA [EC:5.2.1.8] | 0.672 | 1.08e-03 | 4.19e-02\* |
| dhaL; phosphoenolpyruvate---glycerone phosphotransferase subunit DhaL [EC:2.7.1.121] | -0.813 | 1.12e-03 | 4.33e-02\* |
| cbiH60; cobalt-factor III methyltransferase [EC:2.1.1.272] | 0.841 | 1.14e-03 | 4.38e-02\* |
| lmrS; MFS transporter, DHA2 family, multidrug resistance protein | -1.669 | 1.16e-03 | 4.40e-02\* |
| manZ; mannose PTS system EIID component | -1.003 | 1.17e-03 | 4.40e-02\* |
| sanA; SanA protein | 0.989 | 1.17e-03 | 4.40e-02\* |
| sutR; XRE family transcriptional regulator, regulator of sulfur utilization | -0.710 | 1.20e-03 | 4.47e-02\* |
| lpxL, htrB; Kdo2-lipid IVA lauroyltransferase/acyltransferase [EC:2.3.1.241 2.3.1.-] | 0.585 | 1.21e-03 | 4.49e-02\* |
| aat; leucyl/phenylalanyl-tRNA---protein transferase [EC:2.3.2.6] | 0.685 | 1.23e-03 | 4.52e-02\* |
| lyxK; L-xylulokinase [EC:2.7.1.53] | -1.240 | 1.30e-03 | 4.67e-02\* |
| creD; inner membrane protein | 0.635 | 1.30e-03 | 4.67e-02\* |
| wbpE, wlbC; UDP-2-acetamido-2-deoxy-ribo-hexuluronate aminotransferase [EC:2.6.1.98] | -0.905 | 1.31e-03 | 4.67e-02\* |
| hicB; antitoxin HicB | -0.639 | 1.35e-03 | 4.76e-02\* |
| norQ; nitric oxide reductase NorQ protein | -0.736 | 1.38e-03 | 4.83e-02\* |
| pdp; pyrimidine-nucleoside phosphorylase [EC:2.4.2.2] | -0.658 | 1.46e-03 | 5.03e-02 |
| fdhC; formate transporter | 0.575 | 1.47e-03 | 5.03e-02 |
| codB; cytosine permease | -0.887 | 1.47e-03 | 5.03e-02 |
| cca; tRNA nucleotidyltransferase (CCA-adding enzyme) [EC:2.7.7.72 3.1.3.- 3.1.4.-] | -0.526 | 1.48e-03 | 5.03e-02 |
| trmA; tRNA (uracil-5-)-methyltransferase [EC:2.1.1.35] | -1.588 | 1.49e-03 | 5.06e-02 |
| proW; glycine betaine/proline transport system permease protein | -1.309 | 1.52e-03 | 5.13e-02 |
| ispB; octaprenyl-diphosphate synthase [EC:2.5.1.90] | 0.546 | 1.60e-03 | 5.33e-02 |
| rbsD; D-ribose pyranase [EC:5.4.99.62] | -1.029 | 1.64e-03 | 5.44e-02 |
| endA; DNA-entry nuclease | -0.593 | 1.65e-03 | 5.46e-02 |
| ybcJ; ribosome-associated protein | -0.530 | 1.81e-03 | 5.83e-02 |
| yrrT; putative AdoMet-dependent methyltransferase [EC:2.1.1.-] | -1.510 | 1.84e-03 | 5.84e-02 |
| K06971; uncharacterized protein | -1.044 | 1.90e-03 | 6.01e-02 |
| pgi1; glucose-6-phosphate isomerase, archaeal [EC:5.3.1.9] | -1.425 | 1.96e-03 | 6.15e-02 |
| salK; two-component system, NarL family, secretion system sensor histidine kinase SalK | -0.724 | 1.97e-03 | 6.16e-02 |
| dltA; D-alanine--poly(phosphoribitol) ligase subunit 1 [EC:6.1.1.13] | -0.657 | 2.00e-03 | 6.18e-02 |
| pgpC; phosphatidylglycerophosphatase C [EC:3.1.3.27] | 0.797 | 2.00e-03 | 6.18e-02 |
| lipA, LIAS, LIP1, LIP5; lipoyl synthase [EC:2.8.1.8] | 0.530 | 2.04e-03 | 6.22e-02 |
| E3.4.21.96; lactocepin [EC:3.4.21.96] | -1.382 | 2.16e-03 | 6.54e-02 |
| pabC; 4-amino-4-deoxychorismate lyase [EC:4.1.3.38] | 0.995 | 2.19e-03 | 6.59e-02 |
| araN; arabinosaccharide transport system substrate-binding protein | -0.810 | 2.20e-03 | 6.60e-02 |
| bglF, bglP; beta-glucoside PTS system EIICBA component [EC:2.7.1.-] | -0.699 | 2.21e-03 | 6.60e-02 |
| nodU; carbamoyltransferase [EC:2.1.3.-] | -1.414 | 2.24e-03 | 6.64e-02 |
| yqeH; 30S ribosome assembly GTPase | -0.958 | 2.33e-03 | 6.84e-02 |
| K06926; uncharacterized protein | 0.542 | 2.41e-03 | 7.00e-02 |
| strA; streptomycin 3""""""""-kinase [EC:2.7.1.87] | -5.238 | 2.41e-03 | 7.00e-02 |
| emrB; MFS transporter, DHA2 family, multidrug resistance protein | 0.622 | 2.52e-03 | 7.25e-02 |
| regX3; two-component system, OmpR family, response regulator RegX3 | -0.517 | 2.64e-03 | 7.42e-02 |
| mmuP; S-methylmethionine transporter | -1.254 | 2.66e-03 | 7.43e-02 |
| celB, chbC; cellobiose PTS system EIIC component | -0.857 | 2.75e-03 | 7.53e-02 |
| wbpA; UDP-N-acetyl-D-glucosamine dehydrogenase [EC:1.1.1.136] | -0.896 | 2.80e-03 | 7.63e-02 |
| kdpA; potassium-transporting ATPase potassium-binding subunit | -2.375 | 2.81e-03 | 7.63e-02 |
| flgC; flagellar basal-body rod protein FlgC | -0.530 | 2.83e-03 | 7.65e-02 |
| dnr; CRP/FNR family transcriptional regulator, dissimilatory nitrate respiration regulator | -2.498 | 2.84e-03 | 7.66e-02 |
| clpE; ATP-dependent Clp protease ATP-binding subunit ClpE | -0.882 | 2.86e-03 | 7.68e-02 |
| ftsI; cell division protein FtsI (penicillin-binding protein 3) [EC:3.4.16.4] | 0.533 | 2.87e-03 | 7.69e-02 |
| nifH; nitrogenase iron protein NifH | -1.659 | 2.90e-03 | 7.69e-02 |
| MVD, mvaD; diphosphomevalonate decarboxylase [EC:4.1.1.33] | -1.030 | 2.94e-03 | 7.76e-02 |
| cgeB; spore maturation protein CgeB | -1.135 | 3.09e-03 | 8.05e-02 |
| eutN; ethanolamine utilization protein EutN | -0.823 | 3.09e-03 | 8.05e-02 |
| cadC, smtB; ArsR family transcriptional regulator, lead/cadmium/zinc/bismuth-responsive transcriptional repressor | -0.575 | 3.10e-03 | 8.05e-02 |
| crr; sugar PTS system EIIA component [EC:2.7.1.-] | -4.122 | 3.13e-03 | 8.08e-02 |
| E4.1.1.82; phosphonopyruvate decarboxylase [EC:4.1.1.82] | -0.719 | 3.15e-03 | 8.12e-02 |
| mttB; trimethylamine---corrinoid protein Co-methyltransferase [EC:2.1.1.250] | -1.636 | 3.20e-03 | 8.17e-02 |
| rsbU\_P; phosphoserine phosphatase RsbU/P [EC:3.1.3.3] | 0.665 | 3.31e-03 | 8.24e-02 |
| prsA; foldase protein PrsA [EC:5.2.1.8] | -1.244 | 3.38e-03 | 8.35e-02 |
| K16881; mannose-1-phosphate guanylyltransferase / phosphomannomutase [EC:2.7.7.13 5.4.2.8] | -0.886 | 3.46e-03 | 8.48e-02 |
| kdpD; two-component system, OmpR family, sensor histidine kinase KdpD [EC:2.7.13.3] | -1.519 | 3.51e-03 | 8.53e-02 |
| lldG; L-lactate dehydrogenase complex protein LldG | 0.568 | 3.53e-03 | 8.54e-02 |
| efrA; ATP-binding cassette, subfamily B, multidrug efflux pump | -1.905 | 3.56e-03 | 8.56e-02 |
| E3.5.4.3, guaD; guanine deaminase [EC:3.5.4.3] | 0.597 | 3.56e-03 | 8.56e-02 |
| pheA1; chorismate mutase [EC:5.4.99.5] | -2.805 | 3.57e-03 | 8.56e-02 |
| nodJ; lipooligosaccharide transport system permease protein | -2.159 | 3.60e-03 | 8.61e-02 |
| ulaC, sgaA; ascorbate PTS system EIIA or EIIAB component [EC:2.7.1.194] | -1.188 | 3.64e-03 | 8.67e-02 |
| ureI; acid-activated urea channel | -1.174 | 3.72e-03 | 8.82e-02 |
| purS; phosphoribosylformylglycinamidine synthase subunit PurS [EC:6.3.5.3] | -1.259 | 3.80e-03 | 8.92e-02 |
| tam; trans-aconitate 2-methyltransferase [EC:2.1.1.144] | -1.845 | 3.82e-03 | 8.94e-02 |
| nikD, cntD; nickel transport system ATP-binding protein [EC:7.2.2.11] | -3.438 | 3.96e-03 | 9.13e-02 |
| rnmV; ribonuclease M5 [EC:3.1.26.8] | -0.620 | 3.99e-03 | 9.15e-02 |
| icmE, dotG; intracellular multiplication protein IcmE | -2.109 | 4.01e-03 | 9.17e-02 |
| GATM; glycine amidinotransferase [EC:2.1.4.1] | -0.864 | 4.06e-03 | 9.19e-02 |
| mglC; methyl-galactoside transport system permease protein | -0.564 | 4.30e-03 | 9.65e-02 |
| pbp2A; penicillin-binding protein 2A [EC:2.4.1.129 3.4.16.4] | -1.032 | 4.36e-03 | 9.69e-02 |
| srtB; sortase B [EC:3.4.22.71] | -0.718 | 4.43e-03 | 9.74e-02 |
| merP; periplasmic mercuric ion binding protein | 0.505 | 4.44e-03 | 9.74e-02 |
| hydA; sulfhydrogenase subunit alpha [EC:1.12.1.3 1.12.1.5] | -1.244 | 4.44e-03 | 9.74e-02 |
| mhpE; 4-hydroxy 2-oxovalerate aldolase [EC:4.1.3.39] | -0.656 | 4.56e-03 | 9.88e-02 |
| ygaC; uncharacterized protein | -0.950 | 4.58e-03 | 9.88e-02 |
| rimK; ribosomal protein S6--L-glutamate ligase [EC:6.3.2.-] | -1.777 | 4.58e-03 | 9.88e-02 |
| puuE; 4-aminobutyrate aminotransferase [EC:2.6.1.19] | -0.943 | 4.66e-03 | 1.00e-01 |
| ahbD; AdoMet-dependent heme synthase [EC:1.3.98.6] | 0.749 | 4.70e-03 | 1.00e-01 |
| casD, cse5; CRISPR system Cascade subunit CasD | -4.787 | 4.70e-03 | 1.00e-01 |
| mcsA; protein arginine kinase activator | 0.983 | 4.71e-03 | 1.00e-01 |
| mtlA, cmtA; mannitol PTS system EIICBA or EIICB component [EC:2.7.1.197] | -0.604 | 4.93e-03 | 1.03e-01 |
| K09927; uncharacterized protein | -0.777 | 4.99e-03 | 1.04e-01 |
| arlS; two-component system, OmpR family, sensor histidine kinase ArlS [EC:2.7.13.3] | -0.994 | 5.00e-03 | 1.04e-01 |
| K07032; uncharacterized protein | -1.138 | 5.04e-03 | 1.04e-01 |
| virK; uncharacterized protein | -3.447 | 5.06e-03 | 1.04e-01 |
| vanSB, vanS, vanSD; two-component system, OmpR family, sensor histidine kinase VanS [EC:2.7.13.3] | -0.536 | 5.14e-03 | 1.05e-01 |
| inlA; internalin A | -1.844 | 5.18e-03 | 1.06e-01 |
| K07041; uncharacterized protein | -1.309 | 5.33e-03 | 1.08e-01 |
| ppk2; polyphosphate kinase (ADP) [EC:2.7.4.-] | -0.597 | 5.35e-03 | 1.08e-01 |
| sgtB; monofunctional glycosyltransferase [EC:2.4.1.129] | -2.773 | 5.38e-03 | 1.08e-01 |
| recT; recombination protein RecT | -0.567 | 5.45e-03 | 1.08e-01 |
| treC; trehalose-6-phosphate hydrolase [EC:3.2.1.93] | -0.834 | 5.48e-03 | 1.09e-01 |
| hcaT; MFS transporter, PPP family, 3-phenylpropionic acid transporter | -2.345 | 5.53e-03 | 1.09e-01 |
| pbuX; xanthine permease | -0.761 | 5.54e-03 | 1.09e-01 |
| EIF1, SUI1; translation initiation factor 1 | 0.574 | 5.55e-03 | 1.09e-01 |
| msrA, vmlR; macrolide transport system ATP-binding/permease protein | -3.018 | 5.65e-03 | 1.10e-01 |
| menD; 2-succinyl-5-enolpyruvyl-6-hydroxy-3-cyclohexene-1-carboxylate synthase [EC:2.2.1.9] | 0.552 | 5.65e-03 | 1.10e-01 |
| UMF1; MFS transporter, UMF1 family | -0.678 | 5.66e-03 | 1.10e-01 |
| K06884; uncharacterized protein | -2.203 | 5.69e-03 | 1.10e-01 |
| agaR; DeoR family transcriptional regulator, aga operon transcriptional repressor | 0.511 | 5.90e-03 | 1.12e-01 |
| K07461; putative endonuclease | -0.743 | 5.93e-03 | 1.12e-01 |
| rbsU; putative ribose uptake protein | -1.442 | 5.96e-03 | 1.12e-01 |
| sspD; small acid-soluble spore protein D (minor alpha/beta-type SASP) | -0.601 | 5.97e-03 | 1.12e-01 |
| ABCC-BAC; ATP-binding cassette, subfamily C, bacterial | -0.681 | 6.07e-03 | 1.12e-01 |
| mprF, fmtC; phosphatidylglycerol lysyltransferase [EC:2.3.2.3] | -0.667 | 6.08e-03 | 1.12e-01 |
| FUK; fucokinase [EC:2.7.1.52] | -1.182 | 6.14e-03 | 1.12e-01 |
| K07045; uncharacterized protein | -0.539 | 6.18e-03 | 1.13e-01 |
| ycdX; putative hydrolase | 0.726 | 6.23e-03 | 1.13e-01 |
| mobAB; molybdopterin-guanine dinucleotide biosynthesis protein [EC:2.7.7.77] | -1.705 | 6.40e-03 | 1.16e-01 |
| K09777; uncharacterized protein | -0.610 | 6.52e-03 | 1.17e-01 |
| cas5t; CRISPR-associated protein Cas5t | -1.127 | 6.56e-03 | 1.18e-01 |
| dmsB; anaerobic dimethyl sulfoxide reductase subunit B | -0.844 | 6.69e-03 | 1.18e-01 |
| E2.7.3.13; glutamine kinase [EC:2.7.3.13] | -1.097 | 6.72e-03 | 1.18e-01 |
| folK; 2-amino-4-hydroxy-6-hydroxymethyldihydropteridine diphosphokinase [EC:2.7.6.3] | 0.566 | 6.74e-03 | 1.18e-01 |
| pepX; X-Pro dipeptidyl-peptidase [EC:3.4.14.11] | -0.718 | 6.84e-03 | 1.20e-01 |
| ydhP; MFS transporter, DHA1 family, inner membrane transport protein | -0.835 | 7.00e-03 | 1.22e-01 |
| yesN; two-component system, response regulator YesN | -0.763 | 7.13e-03 | 1.23e-01 |
| K11145; ribonuclease III family protein [EC:3.1.26.-] | -0.592 | 7.15e-03 | 1.23e-01 |
| ccdA; cytochrome c-type biogenesis protein | 0.738 | 7.21e-03 | 1.24e-01 |
| proV; glycine betaine/proline transport system ATP-binding protein [EC:7.6.2.9] | -1.057 | 7.52e-03 | 1.28e-01 |
| gltX; nondiscriminating glutamyl-tRNA synthetase [EC:6.1.1.24] | -0.826 | 7.59e-03 | 1.29e-01 |
| E1.2.1.10; acetaldehyde dehydrogenase (acetylating) [EC:1.2.1.10] | -0.836 | 7.62e-03 | 1.29e-01 |
| SOD1; superoxide dismutase, Cu-Zn family [EC:1.15.1.1] | -0.892 | 7.66e-03 | 1.29e-01 |
| spoIIAB; stage II sporulation protein AB (anti-sigma F factor) [EC:2.7.11.1] | -0.533 | 7.85e-03 | 1.32e-01 |
| pepE; dipeptidase E [EC:3.4.13.21] | 1.009 | 7.89e-03 | 1.32e-01 |
| srlD; sorbitol-6-phosphate 2-dehydrogenase [EC:1.1.1.140] | -0.734 | 8.07e-03 | 1.33e-01 |
| bcrA; bacitracin transport system ATP-binding protein | -0.551 | 8.17e-03 | 1.34e-01 |
| queH; epoxyqueuosine reductase [EC:1.17.99.6] | -0.517 | 8.33e-03 | 1.36e-01 |
| HSP20; HSP20 family protein | -0.618 | 8.47e-03 | 1.38e-01 |
| casE, cse3; CRISPR system Cascade subunit CasE | -4.011 | 8.75e-03 | 1.41e-01 |
| ttdB; L(+)-tartrate dehydratase beta subunit [EC:4.2.1.32] | -1.118 | 8.83e-03 | 1.42e-01 |
| cst2, cas7; CRISPR-associated protein Cst2 | -0.836 | 8.98e-03 | 1.44e-01 |
| tfoX; DNA transformation protein and related proteins | -2.107 | 9.05e-03 | 1.45e-01 |
| tagT\_U\_V; polyisoprenyl-teichoic acid--peptidoglycan teichoic acid transferase [EC:2.7.8.-] | -0.617 | 9.17e-03 | 1.45e-01 |
| ict-Y; itaconate CoA-transferase [EC:2.8.3.-] | -1.101 | 9.25e-03 | 1.45e-01 |
| proX; glycine betaine/proline transport system substrate-binding protein | -1.621 | 9.41e-03 | 1.47e-01 |
| pelG; polysaccharide biosynthesis protein PelG | -1.093 | 9.41e-03 | 1.47e-01 |
| AGA, aspG; N4-(beta-N-acetylglucosaminyl)-L-asparaginase [EC:3.5.1.26] | -1.125 | 9.49e-03 | 1.47e-01 |
| rihC; non-specific riboncleoside hydrolase [EC:3.2.-.-] | -0.732 | 9.52e-03 | 1.47e-01 |
| K03710; GntR family transcriptional regulator | -0.521 | 9.53e-03 | 1.47e-01 |
| OGG1; N-glycosylase/DNA lyase [EC:3.2.2.- 4.2.99.18] | -0.723 | 9.63e-03 | 1.48e-01 |
| wcaC; putative colanic acid biosynthesis glycosyltransferase [EC:2.4.-.-] | 0.554 | 9.92e-03 | 1.51e-01 |
| pbpB; penicillin-binding protein 2B | -1.176 | 1.02e-02 | 1.53e-01 |
| ulaB, sgaB; ascorbate PTS system EIIB component [EC:2.7.1.194] | -0.900 | 1.04e-02 | 1.55e-01 |
| ygjK; putative isomerase | 0.647 | 1.05e-02 | 1.56e-01 |
| flgL; flagellar hook-associated protein 3 FlgL | -0.669 | 1.05e-02 | 1.56e-01 |
| nukF, mcdF, sboF; lantibiotic transport system ATP-binding protein | -0.527 | 1.10e-02 | 1.61e-01 |
| urtE; urea transport system ATP-binding protein | 2.531 | 1.11e-02 | 1.62e-01 |
| K09707; uncharacterized protein | -0.716 | 1.13e-02 | 1.63e-01 |
| czcB, cusB, cnrB; membrane fusion protein, heavy metal efflux system | 0.888 | 1.17e-02 | 1.66e-01 |
| carC; caffeyl-CoA reductase-Etf complex subunit CarC [EC:1.3.1.108] | -1.135 | 1.17e-02 | 1.66e-01 |
| mvaA; hydroxymethylglutaryl-CoA reductase [EC:1.1.1.88] | -0.725 | 1.17e-02 | 1.66e-01 |
| yeiL; CRP/FNR family transcriptional regulator, putaive post-exponential-phase nitrogen-starvation regulator | 2.205 | 1.18e-02 | 1.67e-01 |
| HELQ; POLQ-like helicase [EC:3.6.4.12] | -3.332 | 1.20e-02 | 1.68e-01 |
| hddA; D-glycero-alpha-D-manno-heptose-7-phosphate kinase [EC:2.7.1.168] | -0.525 | 1.20e-02 | 1.68e-01 |
| argF; N-acetylornithine carbamoyltransferase [EC:2.1.3.9] | -0.603 | 1.20e-02 | 1.68e-01 |
| K07493; putative transposase | -0.593 | 1.21e-02 | 1.68e-01 |
| OGDH, sucA; 2-oxoglutarate dehydrogenase E1 component [EC:1.2.4.2] | 1.268 | 1.22e-02 | 1.69e-01 |
| cggR; central glycolytic genes regulator | -1.028 | 1.22e-02 | 1.69e-01 |
| K07004; uncharacterized protein | 1.079 | 1.25e-02 | 1.73e-01 |
| ylbA, UGHY; (S)-ureidoglycine aminohydrolase [EC:3.5.3.26] | 1.648 | 1.27e-02 | 1.75e-01 |
| MuB; ATP-dependent target DNA activator [EC:3.6.1.3] | -0.747 | 1.28e-02 | 1.76e-01 |
| mlhB, chnC; epsilon-lactone hydrolase [EC:3.1.1.83] | -0.560 | 1.31e-02 | 1.78e-01 |
| levG; fructose PTS system EIID component | -0.841 | 1.32e-02 | 1.79e-01 |
| evaA, eryBVI, tylCVI, tylX3, staJ; dTDP-4-dehydro-6-deoxy-alpha-D-glucopyranose 2,3-dehydratase [EC:4.2.1.159] | -2.118 | 1.36e-02 | 1.83e-01 |
| chr, crh; catabolite repression HPr-like protein | -0.693 | 1.37e-02 | 1.84e-01 |
| slyD; FKBP-type peptidyl-prolyl cis-trans isomerase SlyD [EC:5.2.1.8] | 0.651 | 1.39e-02 | 1.85e-01 |
| folE2; GTP cyclohydrolase IB [EC:3.5.4.16] | 1.166 | 1.40e-02 | 1.86e-01 |
| cydD; ATP-binding cassette, subfamily C, bacterial CydD | -0.671 | 1.40e-02 | 1.86e-01 |
| msrA; peptide-methionine (S)-S-oxide reductase [EC:1.8.4.11] | -0.865 | 1.45e-02 | 1.89e-01 |
| dltB; membrane protein involved in D-alanine export | -0.840 | 1.45e-02 | 1.89e-01 |
| lpqC; polyhydroxybutyrate depolymerase | 0.680 | 1.45e-02 | 1.89e-01 |
| nukG, mcdG, sboG; lantibiotic transport system permease protein | -0.528 | 1.45e-02 | 1.89e-01 |
| K09793; uncharacterized protein | 1.002 | 1.47e-02 | 1.90e-01 |
| disA; diadenylate cyclase [EC:2.7.7.85] | -2.191 | 1.47e-02 | 1.90e-01 |
| K07015; uncharacterized protein | -0.518 | 1.48e-02 | 1.91e-01 |
| hslV, clpQ; ATP-dependent HslUV protease, peptidase subunit HslV [EC:3.4.25.2] | -0.711 | 1.51e-02 | 1.93e-01 |
| K06940; uncharacterized protein | 0.541 | 1.51e-02 | 1.93e-01 |
| treZ, glgZ; maltooligosyltrehalose trehalohydrolase [EC:3.2.1.141] | 1.121 | 1.52e-02 | 1.93e-01 |
| vgrG; type VI secretion system secreted protein VgrG | 1.368 | 1.54e-02 | 1.95e-01 |
| tatA; sec-independent protein translocase protein TatA | 0.567 | 1.55e-02 | 1.95e-01 |
| czcC, cusC, cnrC; outer membrane protein, heavy metal efflux system | 1.630 | 1.55e-02 | 1.95e-01 |
| ssrB; two-component system, LuxR family, secretion system response regulator SsrB | -2.505 | 1.58e-02 | 1.97e-01 |
| gatC; galactose PTS system EIIC component | -4.420 | 1.62e-02 | 2.00e-01 |
| fctD; glutamate formiminotransferase / 5-formyltetrahydrofolate cyclo-ligase [EC:2.1.2.5 6.3.3.2] | 0.735 | 1.64e-02 | 2.03e-01 |
| E3.1.21.7, nfi; deoxyribonuclease V [EC:3.1.21.7] | -1.327 | 1.65e-02 | 2.03e-01 |
| GGTA1; N-acetyllactosaminide 3-alpha-galactosyltransferase [EC:2.4.1.87] | 0.656 | 1.69e-02 | 2.06e-01 |
| araR; GntR family transcriptional regulator, arabinose operon transcriptional repressor | -0.784 | 1.73e-02 | 2.08e-01 |
| K07270; glycosyl transferase, family 25 | -2.793 | 1.75e-02 | 2.09e-01 |
| bmrR; MerR family transcriptional regulator, activator of bmr gene | -5.192 | 1.76e-02 | 2.10e-01 |
| GMPP; mannose-1-phosphate guanylyltransferase [EC:2.7.7.13] | 1.282 | 1.78e-02 | 2.12e-01 |
| prlF, sohA; antitoxin PrlF | -0.617 | 1.83e-02 | 2.17e-01 |
| lgaC; L-galactonate 5-dehydrogenase [EC:1.1.1.414] | 0.747 | 1.84e-02 | 2.18e-01 |
| nikB, cntB; nickel transport system permease protein | -2.181 | 1.85e-02 | 2.18e-01 |
| menC; O-succinylbenzoate synthase [EC:4.2.1.113] | 0.961 | 1.86e-02 | 2.18e-01 |
| E2.4.2.6; nucleoside deoxyribosyltransferase [EC:2.4.2.6] | -0.986 | 1.86e-02 | 2.18e-01 |
| dgoT; MFS transporter, ACS family, D-galactonate transporter | 0.511 | 1.86e-02 | 2.18e-01 |
| PRDX2\_4, ahpC; peroxiredoxin 2/4 [EC:1.11.1.24] | 1.503 | 1.87e-02 | 2.18e-01 |
| K15640, phoE; uncharacterized phosphatase | -1.547 | 1.88e-02 | 2.19e-01 |
| mglB; methyl-galactoside transport system substrate-binding protein | -0.605 | 1.88e-02 | 2.19e-01 |
| cysA; sulfate/thiosulfate transport system ATP-binding protein [EC:7.3.2.3] | -0.798 | 1.89e-02 | 2.19e-01 |
| glnR; MerR family transcriptional regulator, glutamine synthetase repressor | -1.374 | 1.93e-02 | 2.22e-01 |
| malR; two-component system, CitB family, response regulator MalR | 2.698 | 1.94e-02 | 2.23e-01 |
| mtbC; dimethylamine corrinoid protein | -1.208 | 1.95e-02 | 2.23e-01 |
| hycG; formate hydrogenlyase subunit 7 | -1.967 | 1.95e-02 | 2.23e-01 |
| kptA; putative RNA 2'-phosphotransferase [EC:2.7.1.-] | -0.775 | 1.96e-02 | 2.23e-01 |
| iolU; scyllo-inositol 2-dehydrogenase (NADP+) [EC:1.1.1.-] | -0.867 | 1.97e-02 | 2.24e-01 |
| norB; nitric oxide reductase subunit B [EC:1.7.2.5] | 0.568 | 1.98e-02 | 2.24e-01 |
| K13290; serine---pyruvate transaminase [EC:2.6.1.51] | -1.191 | 1.99e-02 | 2.25e-01 |
| pelF; polysaccharide biosynthesis protein PelF | -0.680 | 2.03e-02 | 2.28e-01 |
| atzF; allophanate hydrolase [EC:3.5.1.54] | -2.127 | 2.07e-02 | 2.31e-01 |
| bacC; dihydroanticapsin dehydrogenase [EC:1.1.1.385] | 0.638 | 2.10e-02 | 2.32e-01 |
| K07039; uncharacterized protein | -0.656 | 2.10e-02 | 2.32e-01 |
| E2.4.1.5; dextransucrase [EC:2.4.1.5] | -1.033 | 2.10e-02 | 2.32e-01 |
| algD; GDP-mannose 6-dehydrogenase [EC:1.1.1.132] | 0.696 | 2.12e-02 | 2.34e-01 |
| xtmA; phage terminase small subunit | -0.712 | 2.13e-02 | 2.34e-01 |
| lytF, cwlE; peptidoglycan DL-endopeptidase LytF [EC:3.4.-.-] | -1.121 | 2.15e-02 | 2.36e-01 |
| glpR; DeoR family transcriptional regulator, glycerol-3-phosphate regulon repressor | -2.563 | 2.15e-02 | 2.36e-01 |
| hyaA, hybO; hydrogenase small subunit [EC:1.12.99.6] | 0.799 | 2.17e-02 | 2.37e-01 |
| yejB; microcin C transport system permease protein | 1.549 | 2.23e-02 | 2.40e-01 |
| rlmL, rlmK; 23S rRNA (guanine2445-N2)-methyltransferase / 23S rRNA (guanine2069-N7)-methyltransferase [EC:2.1.1.173 2.1.1.264] | -1.240 | 2.24e-02 | 2.40e-01 |
| cobH-cbiC; precorrin-8X/cobalt-precorrin-8 methylmutase [EC:5.4.99.61 5.4.99.60] | -0.523 | 2.24e-02 | 2.40e-01 |
| K09807; uncharacterized protein | 0.743 | 2.25e-02 | 2.40e-01 |
| echB; ech hydrogenase subunit B | -1.435 | 2.26e-02 | 2.40e-01 |
| gluQ; glutamyl-Q tRNA(Asp) synthetase [EC:6.1.1.-] | 1.273 | 2.28e-02 | 2.41e-01 |
| treR2, treR; GntR family transcriptional regulator, trehalose operon transcriptional repressor | -0.861 | 2.29e-02 | 2.41e-01 |
| gudP; MFS transporter, ACS family, glucarate transporter | -1.277 | 2.31e-02 | 2.41e-01 |
| ttrB; tetrathionate reductase subunit B | -2.290 | 2.32e-02 | 2.42e-01 |
| K20885; beta-1,2-mannobiose phosphorylase / 1,2-beta-oligomannan phosphorylase [EC:2.4.1.339 2.4.1.340] | 0.889 | 2.33e-02 | 2.42e-01 |
| garR, glxR; 2-hydroxy-3-oxopropionate reductase [EC:1.1.1.60] | -0.635 | 2.37e-02 | 2.45e-01 |
| eutS; ethanolamine utilization protein EutS | -0.818 | 2.39e-02 | 2.46e-01 |
| cpdA; 3',5'-cyclic-AMP phosphodiesterase [EC:3.1.4.53] | -1.625 | 2.39e-02 | 2.46e-01 |
| togB; oligogalacturonide transport system substrate-binding protein | -1.071 | 2.40e-02 | 2.46e-01 |
| wcaB; putative colanic acid biosynthesis acetyltransferase WcaB [EC:2.3.1.-] | 2.020 | 2.50e-02 | 2.52e-01 |
| DHODH, pyrD; dihydroorotate dehydrogenase [EC:1.3.5.2] | 0.970 | 2.51e-02 | 2.53e-01 |
| patA, rscA, lmrC, satA; ATP-binding cassette, subfamily B, multidrug efflux pump | -0.935 | 2.52e-02 | 2.53e-01 |
| braR, bceR; two-component system, OmpR family, response regulator protein BraR/BceR | -0.573 | 2.52e-02 | 2.53e-01 |
| aprA; adenylylsulfate reductase, subunit A [EC:1.8.99.2] | -1.110 | 2.54e-02 | 2.54e-01 |
| licT, bglG; beta-glucoside operon transcriptional antiterminator | -1.041 | 2.56e-02 | 2.55e-01 |
| insB; insertion element IS1 protein InsB | -2.330 | 2.57e-02 | 2.56e-01 |
| comB; 2-phosphosulfolactate phosphatase [EC:3.1.3.71] | 2.777 | 2.59e-02 | 2.58e-01 |
| E1.2.1.88; 1-pyrroline-5-carboxylate dehydrogenase [EC:1.2.1.88] | -0.920 | 2.60e-02 | 2.58e-01 |
| dptH; DNA phosphorothioation-dependent restriction protein DptH | 0.567 | 2.61e-02 | 2.59e-01 |
| gumF; acyltransferase [EC:2.3.1.-] | 0.670 | 2.63e-02 | 2.60e-01 |
| symE; toxic protein SymE | -0.663 | 2.64e-02 | 2.60e-01 |
| coaA; type I pantothenate kinase [EC:2.7.1.33] | -0.851 | 2.64e-02 | 2.60e-01 |
| K13819; NifU-like protein | -0.761 | 2.66e-02 | 2.62e-01 |
| AKR1A1, adh; alcohol dehydrogenase (NADP+) [EC:1.1.1.2] | -0.617 | 2.67e-02 | 2.62e-01 |
| pilD, pppA; leader peptidase (prepilin peptidase) / N-methyltransferase [EC:3.4.23.43 2.1.1.-] | 1.062 | 2.67e-02 | 2.62e-01 |
| ypeB; spore germination protein | -1.748 | 2.69e-02 | 2.63e-01 |
| pycA; pyruvate carboxylase subunit A [EC:6.4.1.1] | -1.438 | 2.70e-02 | 2.63e-01 |
| mrcB; penicillin-binding protein 1B [EC:2.4.1.129 3.4.16.4] | -1.650 | 2.72e-02 | 2.64e-01 |
| K07118; uncharacterized protein | -0.516 | 2.74e-02 | 2.65e-01 |
| ndh; NADH dehydrogenase [EC:1.6.99.3] | 0.513 | 2.77e-02 | 2.67e-01 |
| minE; cell division topological specificity factor | -0.847 | 2.79e-02 | 2.68e-01 |
| fiu; catecholate siderophore receptor | 2.064 | 2.81e-02 | 2.70e-01 |
| tarL; CDP-ribitol ribitolphosphotransferase / teichoic acid ribitol-phosphate polymerase [EC:2.7.8.14 2.7.8.47] | -1.296 | 2.83e-02 | 2.70e-01 |
| queG; epoxyqueuosine reductase [EC:1.17.99.6] | 0.624 | 2.83e-02 | 2.70e-01 |
| K06867; uncharacterized protein | 0.883 | 2.87e-02 | 2.70e-01 |
| cysI; sulfite reductase (NADPH) hemoprotein beta-component [EC:1.8.1.2] | 2.496 | 2.99e-02 | 2.76e-01 |
| mtlR; mannitol operon transcriptional antiterminator | -1.419 | 2.99e-02 | 2.76e-01 |
| lnuA\_C\_D\_E, lin; lincosamide nucleotidyltransferase A/C/D/E | 1.028 | 3.01e-02 | 2.76e-01 |
| E1.1.1.67, mtlK; mannitol 2-dehydrogenase [EC:1.1.1.67] | -0.744 | 3.02e-02 | 2.76e-01 |
| baiA; 3alpha-hydroxycholanate dehydrogenase (NADP+) [EC:1.1.1.392] | -0.558 | 3.07e-02 | 2.79e-01 |
| murM; serine/alanine adding enzyme [EC:2.3.2.10] | -5.543 | 3.10e-02 | 2.80e-01 |
| iolR; DeoR family transcriptional regulator, myo-inositol catabolism operon repressor | 2.200 | 3.17e-02 | 2.83e-01 |
| mcsB; protein arginine kinase [EC:2.7.14.1] | 1.422 | 3.17e-02 | 2.83e-01 |
| nfrA1; FMN reductase (NADPH) [EC:1.5.1.38] | 0.523 | 3.21e-02 | 2.85e-01 |
| nikC, cntC; nickel transport system permease protein | -1.392 | 3.24e-02 | 2.85e-01 |
| togN; oligogalacturonide transport system permease protein | -0.801 | 3.26e-02 | 2.85e-01 |
| K07150; uncharacterized protein | -0.701 | 3.27e-02 | 2.85e-01 |
| hyaB, hybC; hydrogenase large subunit [EC:1.12.99.6] | 0.830 | 3.28e-02 | 2.86e-01 |
| strB; streptomycin 6-kinase [EC:2.7.1.72] | -2.567 | 3.31e-02 | 2.87e-01 |
| GNE; bifunctional UDP-N-acetylglucosamine 2-epimerase / N-acetylmannosamine kinase [EC:3.2.1.183 2.7.1.60] | 2.133 | 3.32e-02 | 2.87e-01 |
| tagG; teichoic acid transport system permease protein | -0.763 | 3.36e-02 | 2.88e-01 |
| opuBD; osmoprotectant transport system permease protein | -0.675 | 3.37e-02 | 2.88e-01 |
| K09749; uncharacterized protein | -0.907 | 3.39e-02 | 2.89e-01 |
| pduD; propanediol dehydratase medium subunit [EC:4.2.1.28] | -1.105 | 3.41e-02 | 2.90e-01 |
| rluE; 23S rRNA pseudouridine2457 synthase [EC:5.4.99.20] | 1.880 | 3.41e-02 | 2.90e-01 |
| mtnK; 5-methylthioribose kinase [EC:2.7.1.100] | -1.376 | 3.49e-02 | 2.94e-01 |
| xdhD; putative selenate reductase molybdopterin-binding subunit | -0.798 | 3.50e-02 | 2.94e-01 |
| plsB; glycerol-3-phosphate O-acyltransferase [EC:2.3.1.15] | -1.668 | 3.50e-02 | 2.94e-01 |
| lnt; apolipoprotein N-acyltransferase [EC:2.3.1.269] | 1.069 | 3.54e-02 | 2.96e-01 |
| sul2; dihydropteroate synthase type 2 [EC:2.5.1.15] | -3.008 | 3.55e-02 | 2.96e-01 |
| hyaC; Ni/Fe-hydrogenase 1 B-type cytochrome subunit | 1.039 | 3.60e-02 | 2.99e-01 |
| cbiQ; cobalt/nickel transport system permease protein | 0.600 | 3.61e-02 | 2.99e-01 |
| tarI; D-ribitol-5-phosphate cytidylyltransferase [EC:2.7.7.40] | -0.892 | 3.66e-02 | 3.01e-01 |
| E2.7.7.103; L-glutamine-phosphate cytidylyltransferase [EC:2.7.7.103] | -0.763 | 3.66e-02 | 3.01e-01 |
| lytS; two-component system, LytTR family, sensor histidine kinase LytS [EC:2.7.13.3] | -0.786 | 3.66e-02 | 3.01e-01 |
| K09384; uncharacterized protein | -1.652 | 3.69e-02 | 3.02e-01 |
| terZ; tellurium resistance protein TerZ | 0.572 | 3.70e-02 | 3.02e-01 |
| dmsB; dimethyl sulfoxide reductase iron-sulfur subunit | 0.758 | 3.72e-02 | 3.04e-01 |
| araP; arabinosaccharide transport system permease protein | -0.760 | 3.73e-02 | 3.04e-01 |
| ligK, galC; 4-hydroxy-4-methyl-2-oxoglutarate aldolase [EC:4.1.3.17] | -0.881 | 3.85e-02 | 3.11e-01 |
| ddc; L-2,4-diaminobutyrate decarboxylase [EC:4.1.1.86] | -1.539 | 3.86e-02 | 3.12e-01 |
| epsG; transmembrane protein EpsG | -2.222 | 3.86e-02 | 3.12e-01 |
| MET8; precorrin-2 dehydrogenase / sirohydrochlorin ferrochelatase [EC:1.3.1.76 4.99.1.4] | -1.895 | 3.88e-02 | 3.12e-01 |
| gatA, sgcA; galactitol PTS system EIIA component [EC:2.7.1.200] | -4.562 | 3.94e-02 | 3.14e-01 |
| gmhC, hldE, waaE, rfaE; D-beta-D-heptose 7-phosphate kinase / D-beta-D-heptose 1-phosphate adenosyltransferase [EC:2.7.1.167 2.7.7.70] | 1.337 | 3.94e-02 | 3.14e-01 |
| yfhM; alpha-2-macroglobulin | 0.870 | 3.94e-02 | 3.14e-01 |
| cpxP, spy; periplasmic protein CpxP/Spy | 0.607 | 3.97e-02 | 3.15e-01 |
| NANS, SAS; sialic acid synthase [EC:2.5.1.56 2.5.1.57 2.5.1.132] | -0.903 | 3.97e-02 | 3.15e-01 |
| ribT; riboflavin biosynthesis RibT protein | -1.010 | 4.00e-02 | 3.17e-01 |
| ATPVG, ahaH, atpH; V/A-type H+/Na+-transporting ATPase subunit G/H | -2.435 | 4.02e-02 | 3.18e-01 |
| putA; RHH-type transcriptional regulator, proline utilization regulon repressor / proline dehydrogenase / delta 1-pyrroline-5-carboxylate dehydrogenase [EC:1.5.5.2 1.2.1.88] | 2.021 | 4.03e-02 | 3.18e-01 |
| argAB; amino-acid N-acetyltransferase [EC:2.3.1.1] | 1.039 | 4.05e-02 | 3.18e-01 |
| eexD; ATP-binding cassette, subfamily C, bacterial EexD | -1.280 | 4.05e-02 | 3.18e-01 |
| mepA; multidrug efflux pump | -0.503 | 4.05e-02 | 3.18e-01 |
| dapD; 2,3,4,5-tetrahydropyridine-2,6-dicarboxylate N-succinyltransferase [EC:2.3.1.117] | 0.725 | 4.10e-02 | 3.20e-01 |
| sigB; RNA polymerase sigma-B factor | -2.989 | 4.10e-02 | 3.20e-01 |
| nikA, cntA; nickel transport system substrate-binding protein | -1.721 | 4.11e-02 | 3.20e-01 |
| mscK, kefA, aefA; potassium-dependent mechanosensitive channel | 1.124 | 4.12e-02 | 3.20e-01 |
| THG1; tRNA(His) guanylyltransferase [EC:2.7.7.79] | 1.157 | 4.13e-02 | 3.20e-01 |
| gatB, sgcB; galactitol PTS system EIIB component [EC:2.7.1.200] | -0.951 | 4.17e-02 | 3.21e-01 |
| uctC; CoA:oxalate CoA-transferase [EC:2.8.3.19] | -0.548 | 4.18e-02 | 3.21e-01 |
| prkA; serine protein kinase | -1.972 | 4.20e-02 | 3.23e-01 |
| acuB; acetoin utilization protein AcuB | -1.715 | 4.24e-02 | 3.24e-01 |
| ectB, dat; diaminobutyrate-2-oxoglutarate transaminase [EC:2.6.1.76] | -0.988 | 4.26e-02 | 3.25e-01 |
| nisG, spaG, cprC; lantibiotic transport system permease protein | -0.612 | 4.29e-02 | 3.27e-01 |
| torY; trimethylamine-N-oxide reductase (cytochrome c), cytochrome c-type subunit TorY | -2.564 | 4.29e-02 | 3.27e-01 |
| wcaL, amsK; colanic acid/amylovoran biosynthesis glycosyltransferase [EC:2.4.-.-] | -1.133 | 4.32e-02 | 3.28e-01 |
| sspA, mglA; stringent starvation protein A | 0.850 | 4.34e-02 | 3.28e-01 |
| arnC, pmrF; undecaprenyl-phosphate 4-deoxy-4-formamido-L-arabinose transferase [EC:2.4.2.53] | -0.594 | 4.35e-02 | 3.29e-01 |
| K16153; glycogen phosphorylase/synthase [EC:2.4.1.1 2.4.1.11] | 0.519 | 4.35e-02 | 3.29e-01 |
| gabT; 4-aminobutyrate aminotransferase / (S)-3-amino-2-methylpropionate transaminase / 5-aminovalerate transaminase [EC:2.6.1.19 2.6.1.22 2.6.1.48] | -0.739 | 4.36e-02 | 3.29e-01 |
| togM; oligogalacturonide transport system permease protein | -0.673 | 4.39e-02 | 3.30e-01 |
| hndB; NADP-reducing hydrogenase subunit HndB [EC:1.12.1.3] | -0.796 | 4.41e-02 | 3.31e-01 |
| ptcA; putrescine carbamoyltransferase [EC:2.1.3.6] | -1.404 | 4.42e-02 | 3.31e-01 |
| K09939; uncharacterized protein | 1.049 | 4.47e-02 | 3.33e-01 |
| oprO\_P; phosphate-selective porin OprO and OprP | 1.597 | 4.47e-02 | 3.33e-01 |
| K06907; uncharacterized protein | -0.739 | 4.53e-02 | 3.36e-01 |
| K17076, lysY; putative lysine transport system ATP-binding protein [EC:3.6.3.-] | -0.669 | 4.59e-02 | 3.37e-01 |
| K07494; putative transposase | -0.967 | 4.61e-02 | 3.37e-01 |
| astA; arginine N-succinyltransferase [EC:2.3.1.109] | 2.531 | 4.62e-02 | 3.37e-01 |
| E3.1.2.1, ACH1; acetyl-CoA hydrolase [EC:3.1.2.1] | 1.143 | 4.62e-02 | 3.37e-01 |
| tyrP; tyrosine-specific transport protein | -1.665 | 4.64e-02 | 3.37e-01 |
| relE, stbE; mRNA interferase RelE/StbE | -0.783 | 4.65e-02 | 3.37e-01 |
| DLST, sucB; 2-oxoglutarate dehydrogenase E2 component (dihydrolipoamide succinyltransferase) [EC:2.3.1.61] | 0.926 | 4.67e-02 | 3.37e-01 |
| srlB; glucitol/sorbitol PTS system EIIA component [EC:2.7.1.198] | -0.885 | 4.72e-02 | 3.40e-01 |
| fruB; fructose PTS system EIIA component [EC:2.7.1.202] | -1.897 | 4.73e-02 | 3.40e-01 |
| K07063; uncharacterized protein | -0.599 | 4.77e-02 | 3.41e-01 |
| pflD; trans-4-hydroxy-L-proline dehydratase [EC:4.2.1.172] | -0.553 | 4.77e-02 | 3.41e-01 |
| speB; streptopain [EC:3.4.22.10] | 2.937 | 4.78e-02 | 3.41e-01 |
| rbbA; ribosome-dependent ATPase | -1.550 | 4.80e-02 | 3.42e-01 |
| malT; LuxR family transcriptional regulator, maltose regulon positive regulatory protein | -0.686 | 4.80e-02 | 3.42e-01 |
| macB; macrolide transport system ATP-binding/permease protein [EC:3.6.3.-] | -0.871 | 4.82e-02 | 3.42e-01 |
| repA; regulatory protein RepA | -0.820 | 4.85e-02 | 3.44e-01 |
| panC-cmk; pantoate ligase / CMP/dCMP kinase [EC:6.3.2.1 2.7.4.25] | -2.140 | 4.88e-02 | 3.44e-01 |
| arcD, lysl, lysP; arginine:ornithine antiporter / lysine permease | -0.510 | 4.89e-02 | 3.44e-01 |
| rfbG; CDP-glucose 4,6-dehydratase [EC:4.2.1.45] | -0.516 | 4.90e-02 | 3.44e-01 |
| rep; ATP-dependent DNA helicase Rep [EC:3.6.4.12] | -0.706 | 4.92e-02 | 3.44e-01 |
| rusA; crossover junction endodeoxyribonuclease RusA [EC:3.1.22.4] | 4.199 | 4.93e-02 | 3.44e-01 |
| nfeD; membrane-bound serine protease (ClpP class) | 1.038 | 4.95e-02 | 3.45e-01 |
| K09974; uncharacterized protein | 1.372 | 4.96e-02 | 3.45e-01 |
